# Epigenetic insights into extreme longevity in the world’s oldest terrestrial animal, Jonathan

**DOI:** 10.1126/sciadv.adw8887

**Published:** 2026-10-07

**Authors:** Benjamin Vaisvil, Daniel P. Schmitt, Angela Jones, Vinayak Kapatral, James M. Ford, Madison L. Taylor, Mathia Colwell, Jonathan Hollins, Sam Pascucci, Konstantin Weissenow, Burkhard Rost, Pascal Notin, Justin Gerlach, Thomas C. Terwilliger, Li-Wei Hung, Lars Juhl Jensen, Kathlyn Reed, Todd R. Robeck, Steve Horvath, Christopher Faulk, Yanjun Ma, Stephen W. Clark

**Affiliations:** ^1^ Kallel Foundation, Nashville, TN, USA.; ^2^ Igenbio, Inc., Chicago, IL, USA.; ^3^ VANTAGE, Vanderbilt University Medical Center, Nashville, TN, USA.; ^4^ Vanderbilt University, Nashville, TN, USA.; ^5^ Oregon Institute of Occupational Health Sciences, Oregon Health and Science University, Portland, OR, USA.; ^6^ Agriculture and Natural Resources Division, St. Helena Government, Jamestown, St. Helena.; ^7^ Florida Iguana & Tortoise Breeders, Southwest Ranches, FL, USA.; ^8^ Department of Bioinformatics & Computational Biology, Technical University of Munich, Munich, Germany.; ^9^ Systems Biology Department, Harvard Medical School, Boston, MA, USA.; ^10^ Peterhouse, University of Cambridge, Cambridge, UK.; ^11^ New Mexico Consortium, Los Alamos, NM, USA.; ^12^ Los Alamos National Laboratory, Los Alamos, NM, USA.; ^13^ NNF Center for Protein Research, University of Copenhagen, Copenhagen, Denmark.; ^14^ SeaWorld of Texas, San Antonio, TX, USA.; ^15^ United Parks and Resorts, Orlando, FL, USA.; ^16^ The University of California, Los Angeles, Los Angeles, CA, USA.; ^17^ Department of Animal Science, University of Minnesota, Minneapolis, MN, USA.; ^18^ Tennessee Oncology, Nashville, TN, USA.; ^19^ Epiphany Biosciences, San Francisco, CA, USA.; ^20^ Vanderbilt-Ingram Cancer Center, Nashville, TN, USA.

## Abstract

Aldabra giant tortoises (*Aldabrachelys gigantea*) are exceptionally long-lived. We sequenced the genome and methylome of Jonathan, a 194-year-old Aldabra, to explore the molecular basis of his longevity. Relative to other giant tortoises (*A. gigantea* and *Chelonoidis abingdonii*), Jonathan has unique gene variants in most aging pathways. Moreover, Jonathan has substantial DNA methylation and methylation entropy changes compared to four other Aldabras ranging in age from a 5-year-old juvenile to older adults. Notably, we found that lower-entropy regions in Jonathan’s methylome were enriched for the promoters of genes involved in the mitochondrial electron transport chain, and RNA metabolism. This suggests that high-fidelity transcription of the genes in these pathways may be crucial for long-lived species. Our findings support a model for aging wherein the maintenance of low methylation entropy in gene promoters is coupled to efficient mitochondrial energy production, efficient RNA processing, and efficient genomic repair.

## INTRODUCTION

Recent studies on longevity have used comparative genomic analysis, focusing on long-lived animals like the naked mole rat, bowhead whale, Brandt’s bat, immortal jellyfish, and long-lived variants of the African turquoise killifish (*1*–*5*). These investigations have deepened our understanding of gene markers that potentially slow aging and inhibit cancer in these species. For instance, the bowhead whale genome revealed nonsynonymous single-nucleotide variants (nsSNVs) in DNA repair gene *ERCC1*, epigenetic modifiers *HDAC1/2*, duplications in cell cycle regulator *PCNA*, and the mechanistic target of rapamycin (mTOR) pathway gene *LAMTOR1* (*2*). In naked mole rats, genes involved in alternative telomere lengthening, *TOP2A*, *TEP1*, and *TERF1*, were positively selected, while Brandt’s bat showed nsSNVs within the growth hormone and insulin-like growth factor (GH/IGF1) axis (*1*, *3*).

To further explore longevity pathways, we evaluated the genome of Jonathan, an Aldabra giant tortoise (*Aldabrachelys gigantea*) with an estimated age of 194 years, considered the world’s oldest known land animal (see the Supplementary Materials, section S1.1 and figs. S1 to S5). Giant tortoises are known for their long life spans, and some species harbor longevity-associated genetic features (*6*). Prior genomic analyses of two Aldabra giant tortoises and a Galapagos giant tortoise, Lonesome George (*Chelonoidis abingdonii*), identified longevity-associated nsSNVs in genes like *IGF1R*, *DCLRE1B*, and *XRCC6* (*6*, *7*). However, no existing study has assessed whether giant tortoises delay or avoid the epigenetic changes that typically accrue with aging in other vertebrate lineages or whether exceptionally long-lived individuals like Jonathan have unique epigenetic features.

Here, we report the genome sequences and comparative analysis of Jonathan and Tank, a 36-year-old Aldabra giant tortoise living in captivity. Jonathan lives on the Island of Saint Helena in the South Atlantic, a notably different environment than Tank’s captive setting (fig. S6). A recent study using the Species360 Zoological Information Management System and Bayesian survival trajectory analysis estimated the “adult” life expectancy (starting after the age of first reproduction, defined as 25 years for Aldabra tortoises) and aging rates of male Aldabra and Galapagos tortoises (*8*). This study estimated the mean and upper limit of total life expectancy for Aldabra tortoises to be 80.47 and 93.68 years, respectively, aligning with previous reports (*6*, *9*, *10*). This suggests that Jonathan is ∼100 years older than the typical upper limit for his species. Here, we coupled genomic analysis of Jonathan’s genome with examination of his epigenome to identify potential genetic and epigenetic underpinnings of his extraordinary longevity.

## RESULTS

### Genome sequence, assembly, and annotation

Because of concerns for Jonathan’s health and his iconic status on St. Helena, a blood draw for DNA sequencing was not permitted. Instead, we collected buccal scrapes and saliva samples for genomic DNA (gDNA) isolation. Initial attempts to isolate high-molecular-weight (HMW) gDNA from Jonathan’s samples failed, so we used short-read 2 × 150–base pair (bp) Illumina sequencing, obtaining ∼1.1 billion reads totaling 165 Gb (gigabases). A blood draw from Tank, a younger Aldabra tortoise, yielded HMW gDNA, and we generated a reference genome using long-read PacBio HiFi and Omni-C Hi-C reads. Tank’s assembled genome consists of 211 scaffolds totaling ∼2.41 Gb, similar to previous assemblies of the Aldabra and *Chelonoidis* genomes (fig. S7, A and B, and table S1). Jonathan’s Illumina reads achieved a 93% alignment rate against Tank’s genome, with 60× average coverage depth. From this alignment, 6.9 million high-quality variants were called and used to create a consensus reference genome for Jonathan, also ∼2.41 Gb. Using this consensus assembly, 25,738 protein-coding genes were predicted, of which 16,466 were functionally annotated. The mitochondrial genome assembled to a single contig and was 100% identical to the Aldabra reference mitochondrial genome (NC_028438.1) (table S1).

### Jonathan’s biogeography

Current theory suggests that Aldabra tortoises dispersed “out of Africa,” first from the eastern African coast to Madagascar, then to Granitic Seychelles, and lastly to the Aldabra atoll, a UNESCO World Heritage Site now harboring around 100,000 tortoises (Fig. 1A) (*11*, *12*). The Aldabra atoll comprises four islands: Malabar, Grande Terre, Polymnie, and Picard (fig. S6B). While Picard’s tortoise population was eradicated in the 1800s and later repopulated from Malabar and Grande Terre, Polymnie is devoid of tortoises despite a similar environment (*13*). Recent studies using low-coverage sequencing of 30 Aldabra tortoises identified two distinct genetic groups within the Aldabra population (*7*, *14*).

**Fig. 1.**
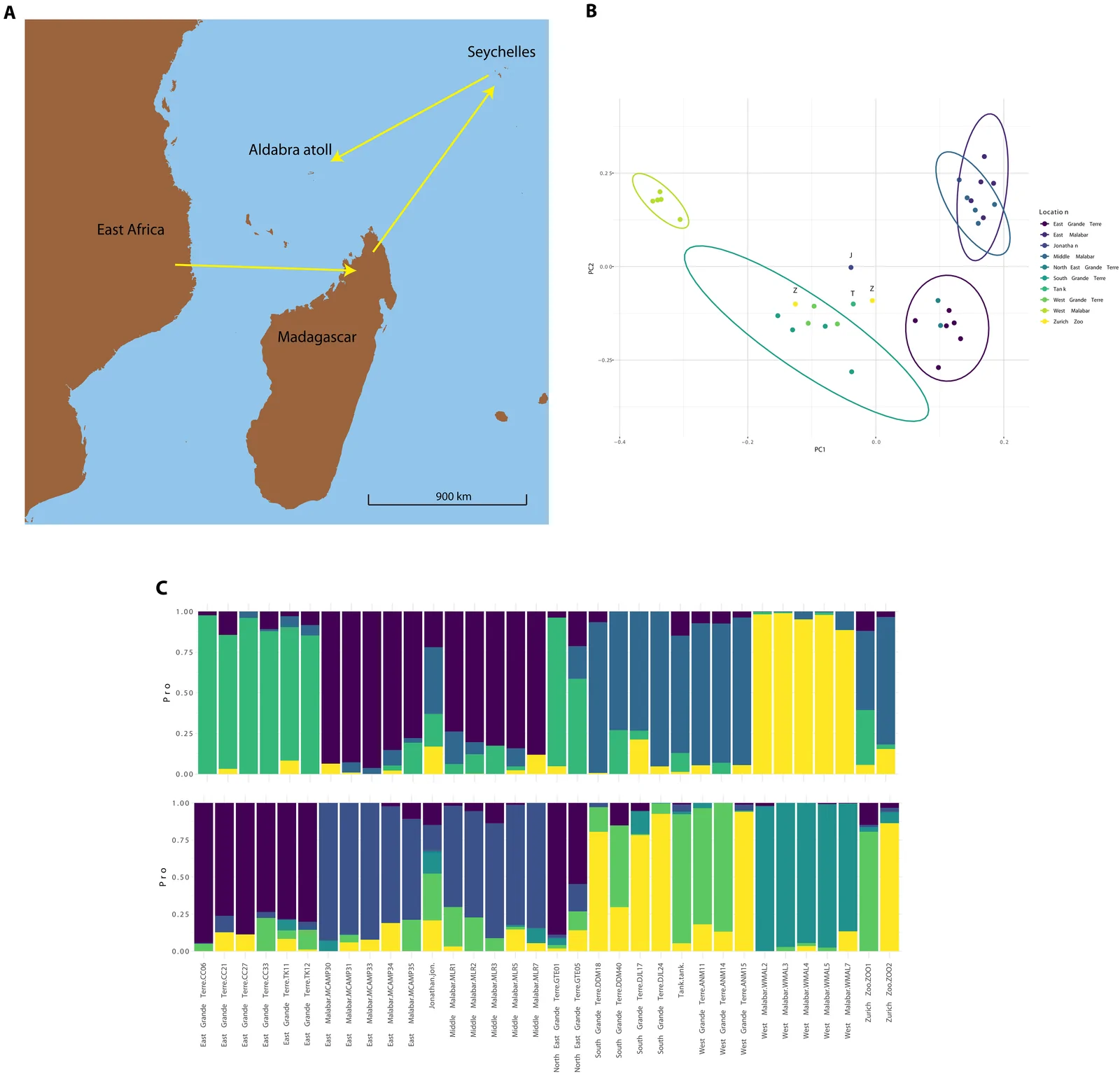
Jonathan’s potential island of origin. (**A**) Current theory on the dispersal route of Aldabras out of Africa (*11*). (**B**) Here, we show a PCA plot of 30 *A. gigantea* genomes from a previous study from the Islands of Malabar and Grande Terre of the Aldabra atoll (table S1). Included are the two Zurich Zoo Aldabras (Z) (*14*). This study adds Tank (T) and Jonathan (J) to the PCA. Principal components 1 and 2 represent 14.2 and 3.64% of the overall genetic variability, respectively. (**C**) Ancestral population *k*-mer plot for four (top panel) or five *k*-mers (bottom panel) for each individual.

Given Jonathan’s exceptional age and the known Aldabra dispersal route, we used these data to investigate his and Tank’s origins. We generated genomic variants for Jonathan, Tank, Lonesome George, and other tortoises using Hermania (a previously sequenced tortoise from the Zurich Zoo) as a reference (*7*, *14*). Principal components analysis (PCA) revealed that Tank, like Hermania and Maleika (another referenced tortoise from the Zurich Zoo), is genetically similar to tortoises from western Grande Terre (Fig. 1B and table S1) (*7*, *14*). Jonathan, however, was positioned closer to Grande Terre but more distantly than Tank, Hermania, or Maleika. This could be because Jonathan was removed from the Aldabra Atoll much earlier or originated from Granitic Seychelles. Given that sailors likely extirpated tortoises from Granitic Seychelles by the early 19th century, and assuming Jonathan’s age of 194, the former scenario is more probable (*15*). In comparison, we analyzed Lonesome George, and as expected, he falls outside the island chain (fig. S8).

Further analysis using unsupervised Bayesian clustering and pairwise linkage analysis, with *K* = 4 in an admixture plot, placed Jonathan closest to Hermania, Maleika, and a South Grande Terre individual. However, at *K* = 5, Jonathan’s admixture was unique (Fig. 1C). These data suggest that Jonathan has a genetic contribution from an unknown source. Whether this is due to population changes over time or his removal from a different island remains unclear.

### Positively selected genes

To understand Jonathan’s unique adaptations and longevity, we analyzed his predicted gene set for signs of positive selection, indicated by a nonsynonymous-to-synonymous mutation ratio (dN/dS) greater than 1. Using other long-lived and short-lived species as a background, we identified 770 significantly (*P* < 0.05) positively selected genes (PSGs) in Jonathan’s genome (Fig. 2A and table S2). We evaluated the functional impact of amino acid changes within these PSGs using the SIFT and PROVEAN algorithms, an approach previously used in studying other long-lived species (*4*, *5*).

**Fig. 2.**
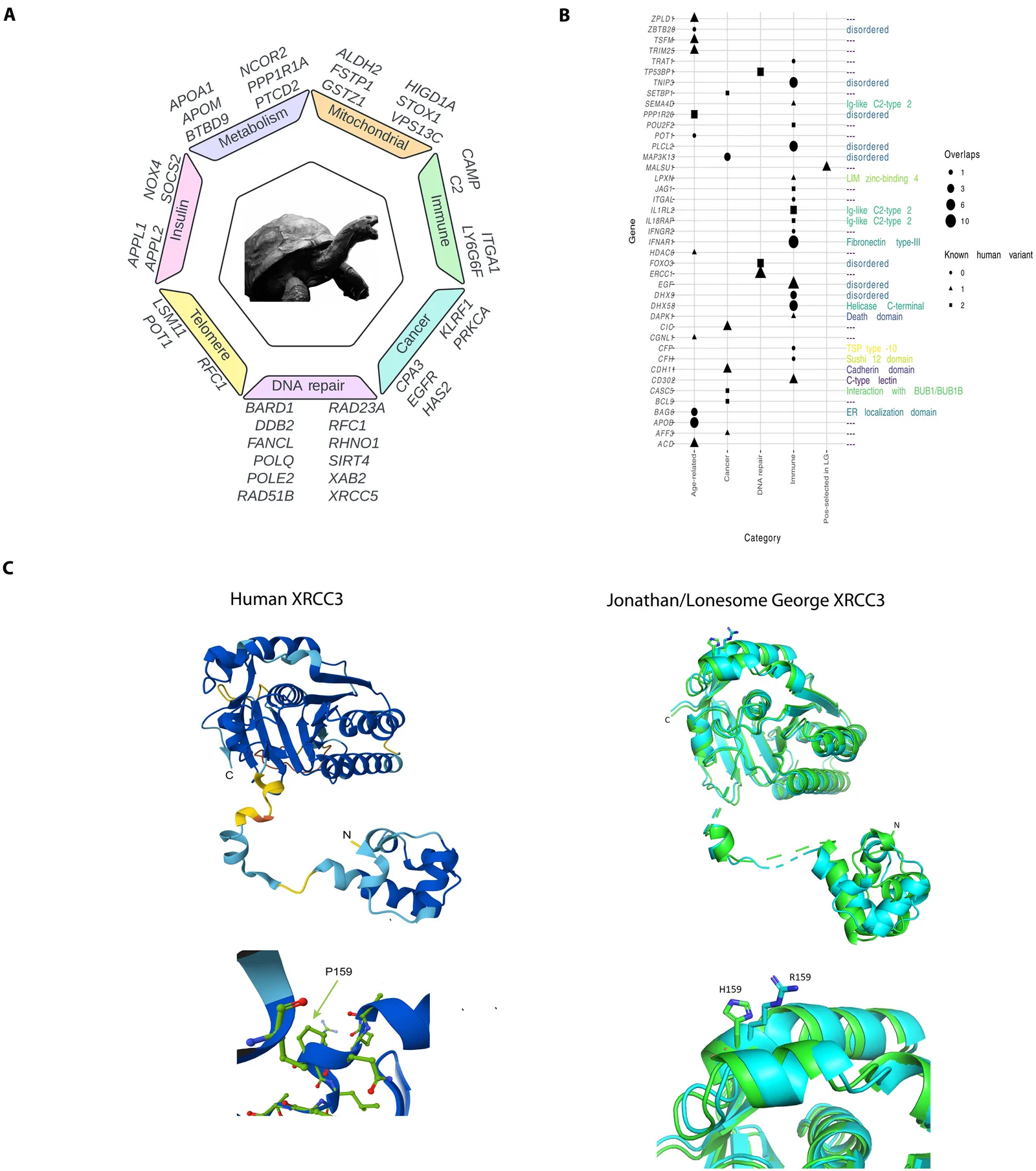
Evolutionary analysis of Jonathan’s genome. (**A**) Subset of genes under positive selection in Jonathan and the categories of function they fall into. (**B**) Jonathan’s unique nsSNVs predicted to be significant by protein language models. (**C**) AlphaFold2 (AF2) predictions. Left panel: AlphaFold prediction found in AlphaFold Protein Structure Database of human *XRCC3* and model confidence (pLDDT). Blue, very high (pLDDT >90); cyan, high (90 > pLDDT > 70); yellow, low (70 > pLDDT > 50); red, very low (pLDDT <50); Right panel: AF2 predictions for Jonathan’s (cyan) and Lonesome George’s (green) *XRCC3*. The N and C termini are labeled. Amino acids are labeled using single-letter codes: proline (P), histidine (H), and arginine (R).

In Jonathan’s genome, we found PSGs involved in DNA repair (*POLE2*, *BARD1*, *RHNO1*, *XAB2*, *RAD23A*, *RAD51B*, *XRCC5*, *DDB2*, *RFC1*, *SIRT4*, *POLQ*, and *FANCL*), insulin regulation (*SOCS2*, *NOX4*, *APPL1*, and *APPL2*), mitochondrial function (*ALDH2*, *HIGD1A*, *VPS13C*, *STOX1*, *GSTZ1*, and *FSTP1*), and telomere function (*POT1*, *RFC1*, and *LSM11*) (*16*). Notably, *LSM11*, part of the U7 snRNP (small nuclear ribonucleoprotein)-telomerase holoenzyme complex, is implicated in the rare human disorder Aicardi-Goutieres syndrome, which can cause premature aging (*17*–*19*). Similarly, *HAS2* (hyaluronic synthase 2), associated with soft tissue sarcoma and mucopolysaccharidosis in humans, is also positively selected in Jonathan (*20*). *HAS2* is involved in excess hyaluronic secretion in the naked mole rat, and transgenic mice overexpressing *HAS2* show cancer resistance and extended life spans (*21*, *22*).

Gene Ontology (GO) enrichment analysis of the PSGs revealed genes involved in development, metabolism, and immunity (fig. S9). Pathways related to aging, including telomerase RNA localization, DNA repair, insulin regulation, and NAD (nicotinamide adenine dinucleotide) biosynthesis, were identified. While NAD levels are known to decrease with age in humans, this relationship has not been investigated in giant tortoises (*23*).

Using the STRING database, we constructed a functional association network of the PSGs and identified clusters related to DNA repair, mitochondrial function, insulin regulation, oxidative stress response, and heart muscle contraction (fig. S10) (*24*). The identification of a cardiovascular function gene cluster is noteworthy, as limited research exists on the Aldabra tortoise’s cardiovascular system, aside from a study indicating a substantially low resting heart rate of 21 beats per minute (*25*).

### nsSNVs shared among giant tortoises

Prior work on Lonesome George manually annotated 2629 longevity-related genes selected a priori from a hypothesis-driven study (*6*). The authors identified 13 nsSNVs affecting known gene motifs or human counterparts related to genetic diseases. Jonathan shares these nsSNVs, including genes involved in DNA repair (*ATM*, *XRCC5*, *XRCC6*, and *TP53*), telomere function (*DCLRE1B*), glucose/insulin regulation (*MIF*, *IGF1R*, *IGF2R*, and *GSK3A*), mitochondrial function (*ALDH2*), and others (fig. S11, A to G) (*16*). Two shared nsSNVs (*ALDH2* and *XRCC5*) are positively selected in Jonathan.

### Unique Jonathan nsSNVs

Given Jonathan’s exceptional age, we compared his genome to Tank, Lonesome George, and humans using the gene set from the Lonesome George study (*6*). We identified 287 genes with unique nsSNVs found only in Jonathan (table S3). Of these genes, 22 are positively selected in Jonathan, including the DNA repair genes *POLQ* and *POLE2* and the telomerase gene *POT1* (table S3).

To evaluate these nsSNVs, we used four protein language models (Tranception, ESM1v, Progen2, and RITA) and one variant effect predictor (VESPA), chosen for their performance and sequence alignment independence (*26*–*31*). We calculated the log-likelihood ratio of mutated human sequences (with Jonathan’s amino acid inserted) versus the reference human sequence and the ratio of mutated Jonathan sequences (with human amino acid inserted) versus Jonathan’s sequence (table S4). This identified 41 genes with potentially functionally significant nsSNVs, 12 of which are in the aging databases LongevityMap and GenAge (Fig. 2B and table S5) (*32*). These include two genes in the telomerase shelterin complex (*ACD* and *POT1*), two in DNA repair (*ERCC1* and *TP53BP1*), one in autophagy (*FOXO3*), and one tumor suppressor (*CIC*) (table S5) (*16*, *33*, *34*). Notably, *POT1*, *FOXO3*, and *ERCC1* variants have been linked to longevity in the immortal jellyfish, Okinawans, and bowhead whales, respectively (*2*, *5*, *35*).

Using AlphaFold2, we generated protein structure predictions for all 287 genes with unique nsSNVs in Jonathan and their Lonesome George orthologs (table S3) (*36*). We primarily observed side-chain differences. We highlight XRCC3 as a key example because its predicted structure achieved the highest quality score [predicted local distance difference test (pLDDT)] among all evaluated proteins, and its substitution offers a clear biophysical contrast. At this position in XRCC3, both Jonathan and Lonesome George have hydrophilic residues [arginine, Pro^159^ → Arg (P159R) and histidine (P159H), respectively], whereas humans have a hydrophobic proline (Fig. 2C). This difference is particularly relevant given *XRCC3*’s role in DNA repair. Although a human *XRCC3* P159R variant exists, it is predicted to be benign (*37*).

### Gene expansion

Previous work identified 11 tortoise-specific gene families with increased copy numbers (*6*). Jonathan has gene expansion in 9 of these 11 genes (table S6). *LAMTOR4*, a member of the Ragulator complex regulating mTOR complex 1 (mTORC1), a key pathway in human longevity, is among the expanded genes (*38*). Lonesome George and all three sequenced Aldabra genomes (Jonathan, Tank, and Hermania) have two copies of *LAMTOR4* (fig. S12, A to C). The second copy (*LAMTOR4JON2*) has five human variants (S41R, R54Q, P62L, H73N, and P96S), two predicted as damaging (*37*). The bowhead whale also has a duplicated *LAMTOR1* with unique nsSNVs, and the killifish shows positive selection for *LAMTOR5* (*2*, *4*).

*VCP* was duplicated in both Lonesome George and Jonathan. *VCP* is involved in DNA damage response, specifically in recruiting TP53BP1 (tumor protein p53 binding protein 1) to double-strand breaks (*39*). Expansion in non–tortoise-specific genes was identified in 16 families in both tortoises, including four tumor suppressor genes, nine immune genes, two cancer genes, and one DNA repair gene (table S6) (*6*, *16*). Notably, Jonathan had 10 copies of the perforin gene (*Prf1*), while Lonesome George had 7. Deletion of this pore-forming protein in mice leads to senescent cell accumulation with age (*40*). It is thought that both natural killer cells and cytotoxic cells rely on the perforin expression for their cytotoxic function, and thus, extra copies of *Prf1* could potentially enhance their immunosurveillance of both senescent and cancer cells.

### Methylome analysis

We performed whole-genome bisulfite sequencing on buccal DNA from Jonathan and four other Aldabras: a 5-year-old juvenile (named “Hatchling” or “Hatch” in the figures), a 12-year-old (Smalls), and two older adults (Toka and Poka, both males). The exact ages of Toka and Poka are not known, but both were adults in 1969. Methylation sequencing yielded 208 to 362 million raw reads per tortoise (61 to 109 Gb of paired-end data). After trimming, filtering, and mapping to our reference genome (Jonathan), all samples had mean coverages between 10- and 17-fold. Bisulfite conversion rates were above 99% for all samples.

Jonathan has decreased methylation globally when compared to the 5-year-old juvenile (Hatch) (Fig. 3, A and B). Lower methylation levels were observed in promoter regions for all tortoises after sites were binned by genomic region type (Fig. 3C). We identified differentially methylated regions (DMRs) between Jonathan and Hatchling (Fig. 3D). In total, 67,091 DMRs were identified, with 60,969 (90.9%) being hypomethylated in Jonathan. These hypomethylated DMRs were primarily located in intergenic (76%) and intronic (19%) regions, with only 5% in promoters or exons. Similarly, hypermethylated DMRs were found in intergenic (60%) and intronic (35%) regions, with 5% in promoters or exons. Jonathan’s hypermethylated DMRs encompassed 218 genes (88 promoter regions and 130 coding regions). GO enrichment of the 218 genes revealed involvement in numerous developmental pathways, including urinary and cardiovascular development (fig. S13, A and B, and table S7).

**Fig. 3.**
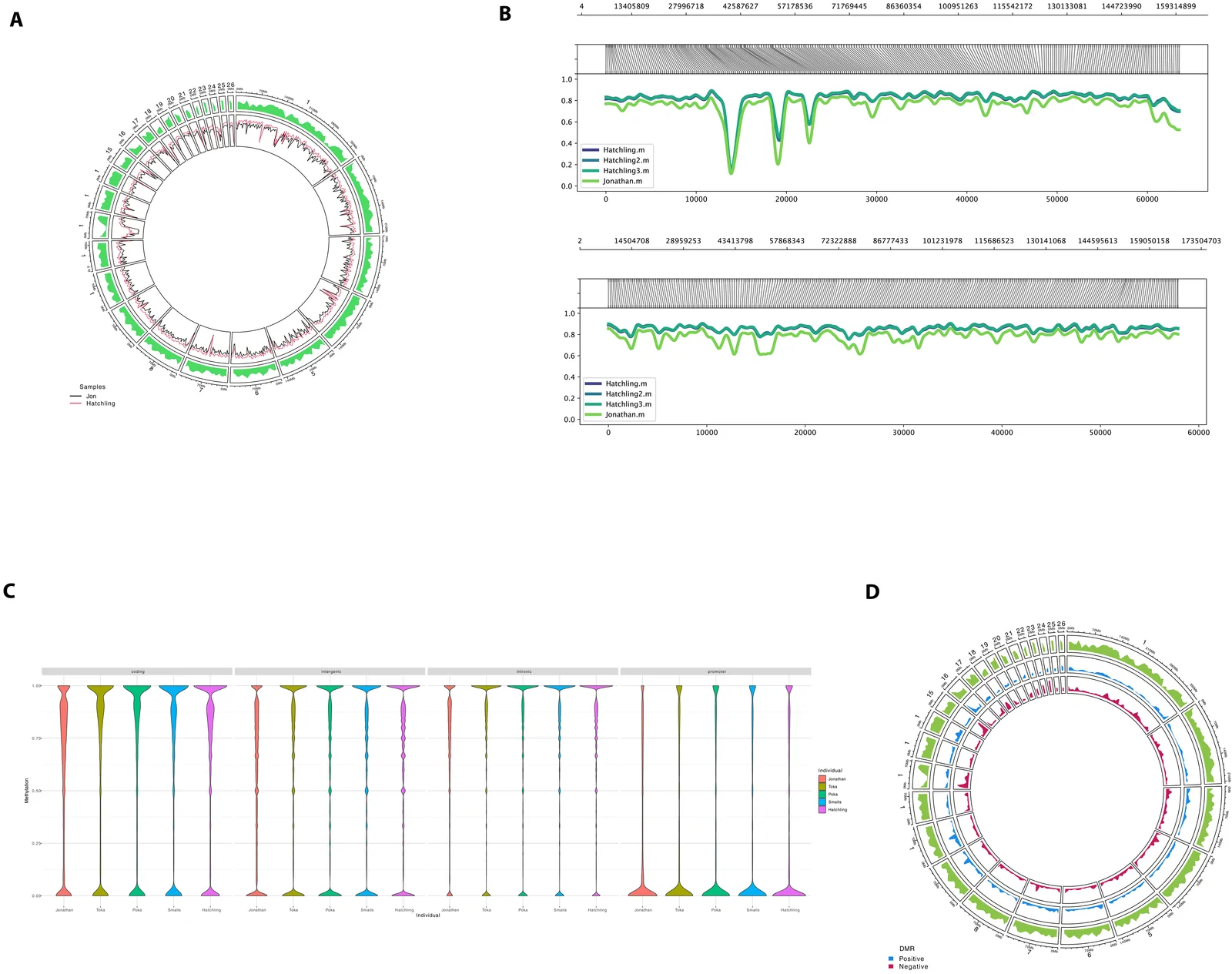
Aldabra tortoise DNA methylome analysis. (**A**) Circos plot of global DNA methylation of Jonathan (black), Hatchling (crimson), and gene density (green). (**B**) Global DNA methylation of Jonathan and Hatchling for contigs 4 (top panel) and 2 (bottom panel). (**C**) Violin plot of DNA methylation across genomic regions for all five tortoises. (**D**) Circos plot of DNA-methylated regions (DMRs) in Jonathan with crimson (negative DMR) and blue (positive DMR) and green (gene density) colors across all contigs. Note that “Hatch” or “Hatchling” denote the same 5-year-old juvenile tortoise.

In mammalian embryonic stem cells, age-related increases in DNA methylation are often seen at polycomb repressive complex 2 (PRC2) target sites (*41*). We identified several PRC2 target genes within Jonathan’s hypermethylated DMRs, including homeobox (*LHX8*, *IRX6*, *MKX*, and *NKX1-2*) and master transcription factor genes (*GATA4*, *RUNX1*, *FOXC2*, *TBX22*, and *TCF21*) (table S7) (*16*).

Previous work has shown that DNA methylation patterns encode crucial biological information, guiding gene expression and regulation (*42*). Over time, stochastic (random) changes can accumulate within these patterns, leading to an increase in what is termed methylation entropy (ME) (*42*, *43*). ME is effectively a measure of the disorder or randomness within the DNA methylation landscape, and an increase in ME reflects a loss of organized methylation patterns characteristic of youthful, healthy cells. Studies have shown that ME increases with age, and if this increase occurs in the promoter region, it correlates with increasing gene expression variance (i.e., transcriptional noise) (*43*–*48*). Consistent with findings in humans, Jonathan had increased ME compared to the younger tortoises (Fig. 4, A and B). This was true across all genomic regions, except promoters, where all five Aldabras had low ME. Moreover, the whole-genome mean ME of each tortoise was significantly predicted by the age of the animal (*P* = 0.01787; *R*^2^ = 0.9821) (Fig. 4C) (*48*).

**Fig. 4.**
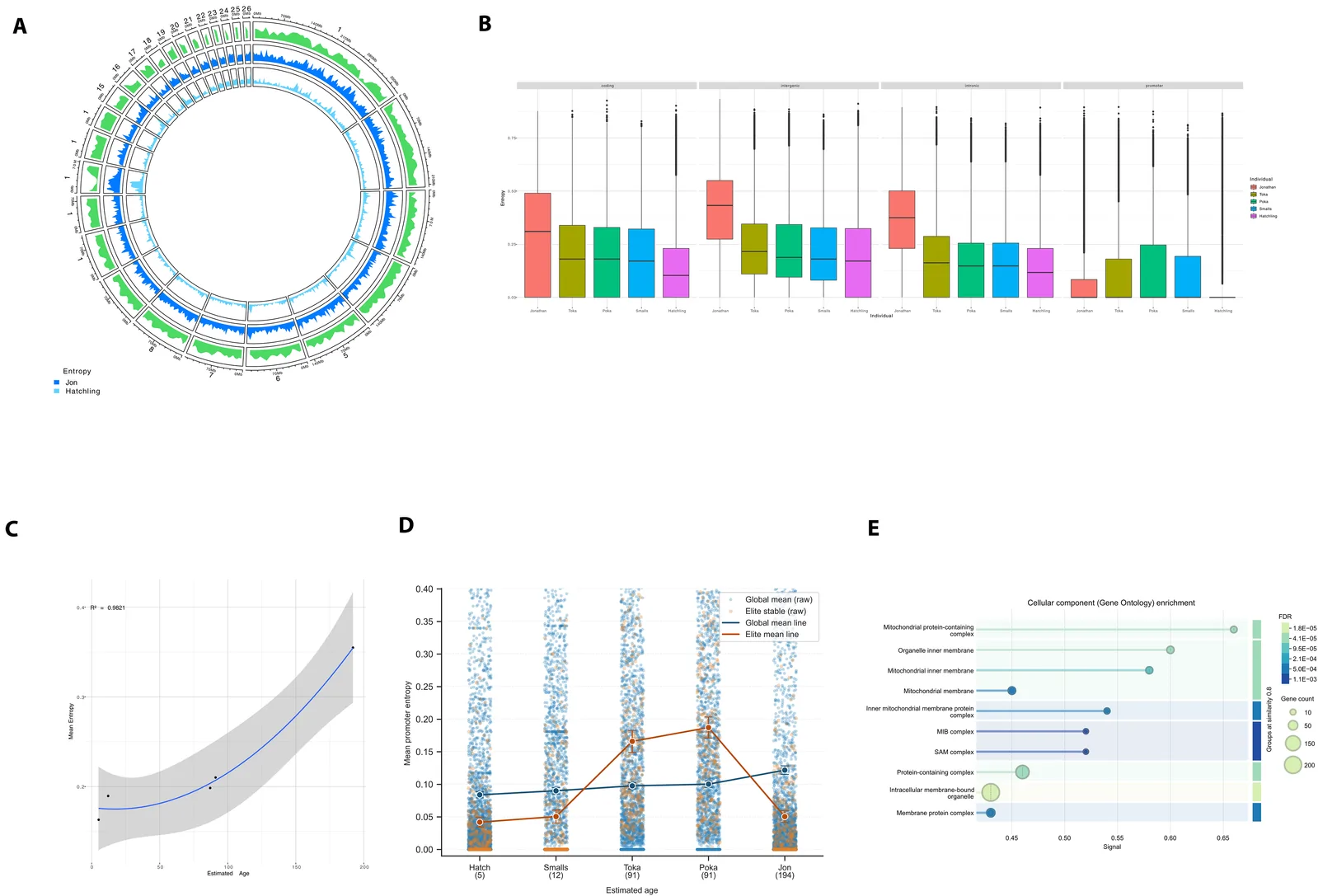
Aldabra tortoise ME analysis. (**A**) Circos plot of ME, comparing Jonathan’s ME (blue) with the hatchling (cyan) and associated plot of gene density (green). (**B**) ME across genomic regions in all five tortoises. (**C**) Global mean entropy in all five tortoises (in order along the *x* axis as hatch, smalls, Toka, Poka, and Jonathan). (**D**) Jitter plot of mean promoter entropy in all promoters evaluated (blue global mean line) and mean promoter entropy in the 272 gene promoters (red elite mean line). (**E**) GO enrichment using the STRING database. Cellular component (GO) enrichment. Note that “Hatch” or “Hatchling” denote the same 5-year-old juvenile tortoise.

Given that ME increases with age, it is hypothesized that lower-entropy regions (LERs), especially in promoters, are more likely to harbor important genes, as maintaining lower entropy requires energy input (*43*, *48*). We examined the methylomes of the tortoises for genes with available promoter ME data and identified 9537 gene promoters with ME data. Of these promoters, 2895 had ME data with a minimum read depth of 10× across all five tortoises (Hatchling, Smalls, Toka, Poka, and Jonathan) (table S8). We used these 2895 promoters to compare promoter ME among the five tortoises.

Mean promoter ME was also highly correlated with tortoise age (Fig. 4D, global mean line) (*P* = 0.003; *R*^2^ = 0.9604). Given that increased promoter ME leads to transcriptional noise, we speculated that genes with low ME in Jonathan and the younger animals (Hatchling and Smalls), but higher ME in the two older animals (Toka and Poka), might be candidates for Jonathan’s longevity. We identified 272 gene promoters that satisfied these conditions (Fig. 4D, elite mean line, and table S8).

Using the STRING protein-protein interaction database, we evaluated the functional network of these 272 genes (*24*). While the gene set was too small for GO “biological process” enrichments, analysis of the “cellular component” category revealed a frequent representation of mitochondrial pathways, including crista formation complexes (MIB and SAM) (Fig. 4E). Next, we examined broader ontological categories containing larger gene counts, specifically “protein-containing complex” and “intracellular membrane-bounded organelle.” Within these categories, mitochondrial organization genes remained represented, alongside genes involved in other distinct processes, including “RNA processing” and “chromosome organization” (fig. S13, C and D).

Upon closer examination of the 272 genes, almost 30% (79 genes) were represented by genes involved in the “hallmarks of aging,” with 13% (35 genes) involved in mitochondrial processes, including complex IV (*COX16*, *COX7A2*, *COX7A2L*, and *COQ10A*), complex I (NDUFB11, *NDUFS7*, and *NDUFA11*) of the electron transport system, and crista formation (*CHCHD3*, *APOO*, and *MICOS13*) and mitophagy (*BECN1* and *PTPN2*) (table S8). Other notable hallmarks were RNA processing/splicing and translation, with 25 genes identified, and genomic/telomere repair, with 15 genes identified (table S8).

Next, we searched the 272 genes against the Open-Genes human longevity database, identifying 11 overlapping genes (table S8) (*49*). Of these, six were mitochondrially related, including two with high confidence (*DGAT1* and *BECN1*).

## DISCUSSION

Here, we identified unique variants in Jonathan involved in aging pathways, including DNA repair, telomere maintenance, autophagy, insulin regulation, mitochondrial function, and cancer. We confirmed the expansion of *LAMTOR4* in Jonathan and showed that the extra copy contains predicted damaging variants that could lead to partial inhibition of mTORC1, an important pathway in longevity (*6*).

We present the whole-genome bisulfite sequencing data of Jonathan, revealing, as expected, that he has an age-related decline in global DNA methylation. This aligns with findings in human centenarians, who exhibit decreased methylation compared to newborns (*50*). Moreover, Jonathan’s hypermethylated DMRs are enriched for developmental genes and targets of PRC2, consistent with data showing age-related hypermethylation in small, CpG-rich regions that encompass PRC2 target genes (*41*, *51*–*54*). It has been proposed that hypermethylation of PRC2 targets leads to loss of stem cell plasticity, resulting in age-related tissue decline (*53*).

Jonathan’s methylome exhibited greater ME than Hatchling’s across most genomic regions, except for the promoters, where ME was comparable. This pattern mirrors findings in humans, where long-lived individuals have increased ME with age but have reduced ME in the promoter regions of centenarians when compared to the elderly (*48*).

Prior work suggests that increased ME, particularly in promoters and regions proximal to the transcriptional start site, is associated with increased transcriptional noise or mean adjusted expression variability (*47*). Consequently, genes whose promoters reside within actively preserved lower-entropy regions in long-lived individuals may be critical for longevity, as their expression is maintained at stable levels. Given the potential importance of LERs within promoters, we analyzed and compared the ME of promoters across the five Aldabra tortoises. We identified 272 gene promoters where Jonathan, Hatchling, and Smalls all had low ME, whereas Toka and Poka had higher ME. We postulated that these genes might explain Jonathan’s exceptional longevity compared to the older tortoises Toka and Poka. Upon analysis, this set included genes involved in mitochondrial organization and RNA metabolism. The latter finding, RNA metabolism, has also been identified in low-ME promoters of long-lived individuals (*48*).

In addition, enrichment was found in genes located within the inner mitochondrial membrane, especially genes involved in oxidative phosphorylation (complexes I and IV). Mitochondria are central to both adenosine 5′-triphosphate (ATP) and reactive oxygen species (ROS) generation, and mitochondrial dysfunction is a hallmark of aging (*55*, *56*). Previous work has shown that longer-lived species have decreased mitochondrial reactive oxygen species production and decreased free radical leak within the electron transport system compared to shorter-lived species (*57*–*59*). Moreover, a recent multiomics study of a 117-year-old woman (the longest-lived verified human) showed that she had exceptionally efficient mitochondrial function compared with an age-matched elderly cohort (*60*). In total, these findings imply that high-fidelity transcription of genes involved in mitochondrial energy production and RNA processing is critical for exceptionally long-lived species.

On the basis of Jonathan’s genetic and epigenetic data, we propose a hypothetical model for aging. This model includes a positive feedback loop, proposed in light of recent work identifying somatic CpG mutations, which arise when methylated cytosine spontaneously deaminates to thymine, as a primary driver of age-related decline in global methylation (Fig. 5) (*61*). This model links efficient DNA repair, efficient ATP production, and efficient RNA processing to the maintenance of LERs in the promoters of genes involved in these processes. In this loop, efficient ATP production supports the genomic repair and restoration of CpG sites and maintenance of LERs within promoters. While maintenance of LERs in this model is consistent with longevity mechanisms/hallmarks, our data are insufficient to support a direct causal inference, and we were only able to evaluate ∼10% of the total tortoise promoter set. Thus, additional studies using blood for deeper methylation coverage will be needed to validate our results.

**Fig. 5.**
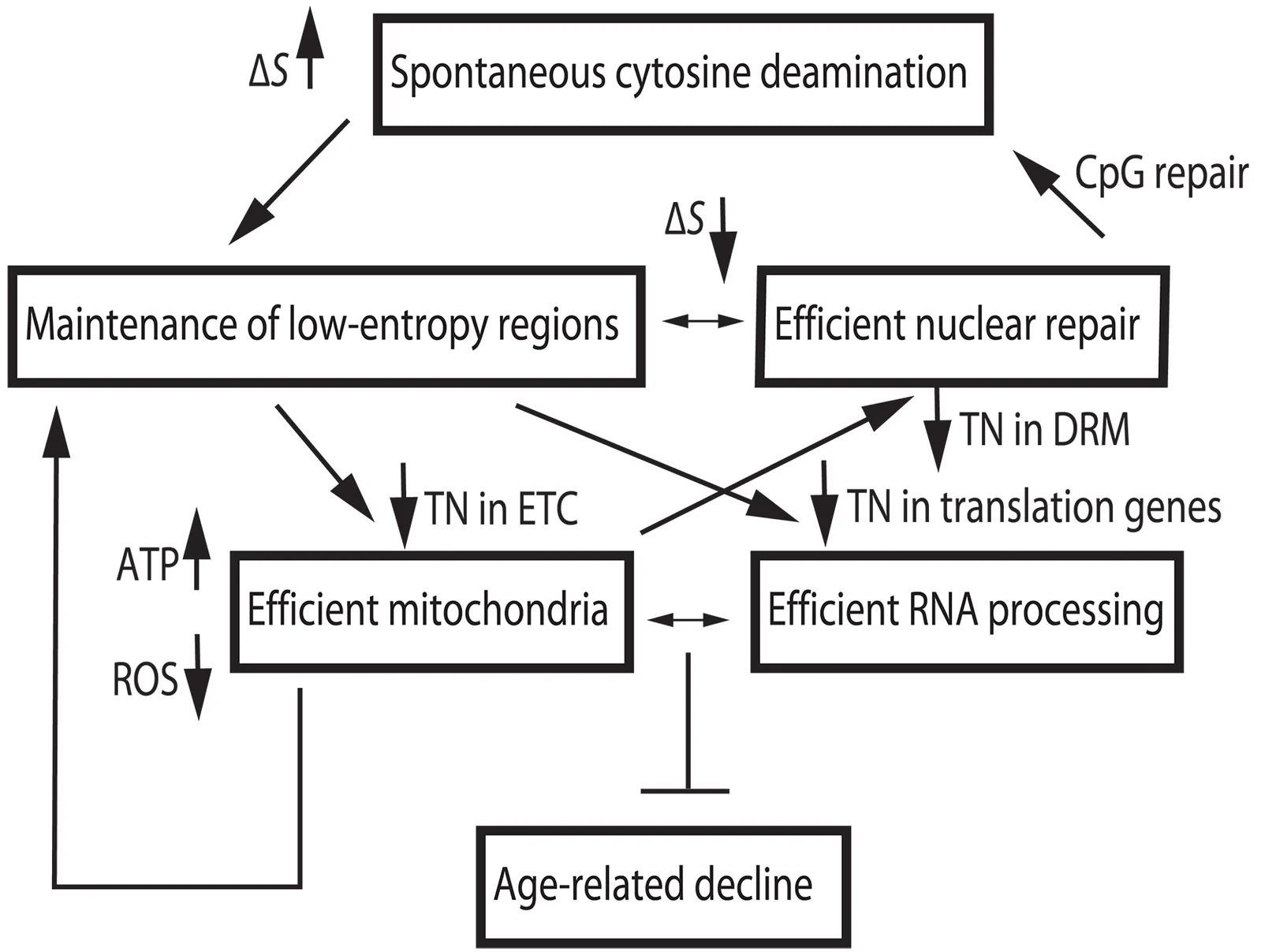
Model for extreme longevity in Jonathan. Spontaneous deamination of cytosine to thymine is a major driver of global demethylation and increased entropy in the genome. In long-lived species, the maintenance of low entropy in the promoter regions of genes involved in mitochondrial function, nuclear repair (DNA, epigenome, and telomers), and RNA processing leads to a reduction in transcriptional noise (TN) in these pathways and their efficient function. A feedback loop is established where efficient mitochondria provide stable production of ATP to maintain an efficient nuclear repair system, whereby repair of TpG to CpG occurs in the promoters of these genes. Moreover, Jonathan’s unique gene variants and PSGs contribute to his efficient nuclear repair. ETC, electron transport chain; DRM, DNA repair mechanism; ROS, reactive oxygen species; Δ*S*, change in entropy.

In summary, while we identified many genetic variants in Jonathan associated with known aging pathways, our most notable finding is the low entropy maintained within specific gene promoters. This stability is exceptionally pronounced in promoters of genes involved in mitochondrial function and RNA processing. We hypothesize that maintaining this low-entropy state ensures high-fidelity expression in these critical pathways, partially protecting him from age-related decline.

### Limitations

Our biggest limitation was the inability to isolate DNA from Jonathan’s blood, as St. Helena officials restricted blood draws to prevent any risk of infection. This prohibited us from using long-read sequencing technologies, isolating RNA, using RNA-seq to verify Jonathan’s unique nsSNVs, and assessing the relationship between Jonathan’s ME and transcriptional variance. To partially overcome this limitation, we generated a reference genome from Tank and used state-of-the-art protein language models to screen for potentially impactful nsSNVs in Jonathan. Moreover, when we compared ME between Jonathan, Hatch, Smalls, Toka, and Poka, we could only use DNA isolated from buccal scrapes, whereas ideally, we would have used blood to be consistent with the reference genome method and achieve higher coverage of more gene promoters. Last, more animal data are needed to determine whether the patterns in ME we identified are specific only to Aldabra tortoises or are a general aspect of aging.

## MATERIALS AND METHODS

### Oral mucosal scrape

Scrapes were taken during the early morning to avoid plant DNA contamination. While pretending to feed Jonathan, he would begin biting the air, and at that point, his mouth was wedged open with a gloved hand while scraping (using the Isohelix buccal scrap kit) under his tongue with the other hand.

### DNA extraction from Jonathan’s oral mucosal scrapes

DNA was extracted from the buccal scrapes using the Buccal-Prep Plus DNA Isolation Kit (Isohelix) with minor modifications. Briefly, 20 μl of proteinase K solution was added to the tube and incubated in a 60°C bath for 1 hour. The liquid in the tube was transferred into a centrifuge using a sterile pipette tip. The tube was centrifuged for 2 min at 18,000*g*. The supernatant from the sample was recovered and added to 400 μl of the kit solution for a total volume of 500 μl. The sample was vortexed briefly. Next, the tube was placed in a microcentrifuge and spun at 12,000*g* for 10 min. The supernatant was poured off, and the tube was respun. Fifty microliters of TE solution was added to the tube. The pellet was resuspended in TE and left at room temperature for 10 min. The sample was then assessed by a NanoDrop.

### DNA extraction from tank’s blood

HMW gDNA was extracted from blood using the MagAttract HMW DNA kit (Qiagen) with minor modifications. Briefly, 10 μl of blood was incubated with 490 μl buffer AL, and incubated overnight at room temperature with 20 μl of proteinase K and 4ul RNase A, followed by incubation at 65°C for 10 minutes. The lysate was brought to room temperature, and 10 μl MagAttract suspension G (beads) and 350 μl buffer MB were added to the lysate for DNA binding. Bead washings were done twice with 700 μl each of buffer MW1, buffer PE, and 80% EtOH. DNA was eluted four times in 100 μl of buffer AE each time.

### Illumina libraries and sequencing

#### Jonathan and Hatchling’s whole-genome bisulfite sequencing

A quality control analysis was performed on the DNA samples using a DNA Qubit assay to determine quantity. The samples were normalized, and libraries were prepared using the NEBNext Enzymatic Methyl-seq kit (P/N: E7120L) following a modified version of the manufacturer’s protocol. This modified version includes the following: We used the following protocol for shearing to try to achieve a larger insert size: sheared to 500 bp (15% duty factor), 450 peak incident power, 200 cycles per burst, 46 s. We used NaOH for the denaturation step instead of formamide. We used H_2_O for all dilutions and resuspensions; no “elution buffer” was used, and we used 200 ng of input material and seven cycles of polymerase chain reaction (PCR). The library quality was assessed using a Bioanalyzer and quantified using a quantitative PCR (qPCR)–based method with the KAPA Library Quantification Kit (P/N: KK4873) and the QuantStudio 12K instrument.

Prepared libraries were pooled in equimolar ratios, and the resulting pool was subjected to cluster generation using the NovaSeq 6000 System, following the manufacturer’s protocols. Paired-end sequencing (150 bp) was performed on the NovaSeq 6000 platform targeting 400 million reads per sample. Raw sequencing data (FASTQ files) obtained from the NovaSeq 6000 were subjected to quality control analysis, including read quality assessment. Real-Time Analysis Software (RTA) and NovaSeq Control Software (NCS) (1.8.0; Illumina) were used for base calling. MultiQC (version 1.7; Illumina) was used for data quality assessments.

#### Whole-genome sequencing

A quality control analysis was performed on the DNA sample using a DNA Qubit assay to determine quantity. The sample was normalized, and the library was prepared using the Twist Biosciences kit (P/N: 104207) following the manufacturer’s protocol. The quality was assessed post–library preparation using the Agilent Bioanalyzer and quantified using a qPCR-based method with the KAPA Library Quantification Kit (P/N: KK4873) and the QuantStudio 12K instrument.

Prepared library pools were pooled in equimolar ratios, and the resulting pool was subjected to cluster generation using the NovaSeq 6000 System, following the manufacturer’s protocols. Paired-end sequencing (150 bp) was performed on the NovaSeq 6000 platform targeting 400 million reads per sample. Raw sequencing data (FASTQ files) obtained from the NovaSeq 6000 were subjected to quality control analysis, including read quality assessment. RTA and NCS (version 1.8.0; Illumina) were used for base calling. MultiQC (version 1.7; Illumina) was used for data quality assessments.

#### Tank’s RNA sequencing

A quality control analysis was performed on the RNA sample using the Agilent Bioanalyzer to assess quality, and an RNA Qubit assay was used to determine quantity. Poly(A) (polyadenylate) RNA enrichment was conducted using the NEBNext Poly(A) mRNA Magnetic Isolation Module (NEB), and the sequencing library was constructed by using the NEBNext Ultra II RNA Library Prep Kit (NEB) (P/N: E7765L, NEB, Ipswich, MA) following the manufacturer’s instructions. End repair, A-tailing, and adapter ligation were performed to generate the final cDNA library. The library quality was assessed using a Bioanalyzer and quantified using a qPCR-based method with the KAPA Library Quantification Kit (P/N: KK4873) and the QuantStudio 12K instrument.

Prepared libraries were pooled in equimolar ratios, and the resulting pool was subjected to cluster generation using the NovaSeq 6000 System, following the manufacturer’s protocols. Paired-end sequencing (150 bp) was performed on the NovaSeq 6000 platform targeting 50 million reads per sample. Raw sequencing data (FASTQ files) obtained from the NovaSeq 6000 were subjected to quality control analysis, including read quality assessment. RTA and NCS (1.8.0; Illumina) were used for base calling. MultiQC (version 1.7; Illumina) was used for data quality assessments.

#### PacBio libraries and sequencing of Tank

HMW gDNA (8 μg) was sheared on a Megaruptor 3 (Diagenode, NJ). Sheared DNAs were size selected for fragments 10 to 20 kb in a BluePippin (Sage Science, MA) using a 0.75% agarose gel Cassette. The PacBio HiFi library was prepared from 2 μg of size-selected DNA with the SMRTBell Express Template Prep kit 2.0. Sequencing was performed on three SMRT cells on a Pacbio Sequel IIe (Pacific Biosciences, CA) using 30-hour movie times. The circular consensus bam and fastq.gz files were generated on an instrument with the software SMRT Link version 10.2 from Pacific Biosciences, which generates consensus sequences from a minimum of three passes around the template with a minimum predicted accuracy of 99%.

#### Omni-C libraries and sequencing of Tank

Proximity ligation Omni-C libraries were constructed with the Omni-C Library Preparation kit from Dovetail Genomics, CA. Ten microliters of blood was used to prepare two libraries. The libraries were constructed exactly as described in the manufacturer’s protocol. The individually barcoded libraries were quantitated with Qubit, run on a fragment analyzer (Agilent, CA) to determine library fragment lengths, and further quantitated by qPCR on a CFX Connect Real-Time qPCR system (Bio-Rad, CA) for accurate pooling of barcoded libraries and maximization of number of clusters in the flow cell. The libraries were sequenced on an SP lane with 2 × 150–nt (nucleotide) reads in a NovaSeq 6000 (Illumina, CA). FASTQ files were generated and demultiplexed with the bcl2fastq version 2.20 conversion software (Illumina).

#### Genome assembly and annotation

PacBio HiFi sequencing yielded a total of 108 Gb in ∼6.8 million reads with the longest read reaching 64,815 bp. Illumina sequencing of the Omni-C reads resulted in a total of 1 billion reads in pairs of 150 nt. HiFi sequences were assembled using Hifiasm (version 0.16.1 r375) with default parameters (*62*). This created an assembly with a length of 2.4 Gb in 193 contigs. The assembly has an N50 of 113 Mb, and the largest contig is 377 Mb. The Omni-C reads were used to scaffold the assembly using SALSA version 2.3 (*63*). SALSA was run with three iterations, enzyme set to DNASE, and “attempt to fix mis-assemblies” set to “yes.”

#### Assembly quality assessment

BUSCO (Benchmarking Universal Single-Copy Orthologs) version 5.3.2 was used to measure the quality of the assembly using the sauropsida dataset from OrthoDB (version 10), which contained 7480 orthologs (*64*, *65*). Of those orthologs, 97.4% were complete, 1.4% were complete but duplicated, 0.4% were fragmented, and 2.2% were missing (Fig. 2A).

#### Jonathan’s variation and consensus creation

The Illumina reads from Jonathan were aligned against the Tank reference assembly using BWA-MEM (*66*). The alignment rate was 92.54%. Following GATK version 4.2.6.1 best practices for non–model organism variant calling, PCR and optical duplicates were marked using the “MarkDuplicates” command in GATK (*67*). Next variants were called using “HaplotypeCaller” and genotyped using “GenotypeGVCFs.” This resulted in 7,678,401 variants, which were then filtered using “VariantFiltration,” leaving 6,911,581 high-quality variants. These variants were then used to create a consensus genome using GATK’s “FastaAlternateReferenceMaker.”

#### Population genetic structure analysis

We performed the population genetic structure analysis, as described by Çilingir *et al.* (*7*). Briefly, we subsampled Illumina reads from Tank and Jonathan to match the sequencing level of the previously published data. We aligned those reads against the “AldGig_1.0.” assembly using BWA using BWA-MEM version 0.7.17. We filtered the alignments for quality greater or equal to 20. Next, we used ANGSD version 0.941-24 to create a beagle file containing genotype likelihoods (baq 1, skipTriallelic 1 minQ 20 minInd 16 doMajorMinor 1 doMaf 1 GL 2 only_proper_pairs 1 uniqueOnly doGlf 2 SNP_pval 0.001) (*68*). PCANGSD version 1.2 was used to generate PCA and admixture analysis at various eigenvalues (*69*). The results were plotted using ggplot2.

#### Gene prediction for Tank and Jonathan

First, a custom repeat model was created for Tank’s genome using Dfam TE Tools (https://github.com/Dfam-consortium/TETools) using RepeatModeler (version 2.0.3) (*70*). Next, both genomes were soft masked using RepeatMasker (version 4.1.2-p1) (*71*). RNA-seq from Lonesome George, a member of *Chelonoidis niger abingdonii*, *Gopherus evgoodei*, and Tank was aligned against the reference genomes using STAR (*7*, *72*, *73*). RNA-seq for Lonesome George mapped at rates of 81.57% for Tank and 81.71% for Jonathan. The mapping rates for *G. evgoodei* were 63.44% for Tank and 63.45% for Jonathan. The RNA-seq alignments were used by BRAKER to generate a file of evidence (*74*). Next, GenomeThreader via BRAKER was used to generate protein evidence from both Lonesome George and *G. evgoodei* (*75*). The protein and RNA-seq evidence was then used as input into Augustus, along with the “chicken model,” to generate gene predictions (*76*).

#### Genome annotation and whole-genome alignment

The ERGO Annotation Pipeline was used to annotate gene functions using other eukaryotic genomes (*Homo sapiens*, *Mus musculus*, *Chelonoidis abingodonii*, and others) as references (*77*, *78*).

#### Identification of PSGs

First, the longest transcript was from each gene of the *H. sapiens* genome. Protein similarity between all coding sequences between all genomes was computed using DIAMOND (version 2.0.8) using the “--ultra-sensitive” option (*79*). Next, using custom scripts, coding sequences with corresponding, bidirectional best hits were selected. Incomplete transcripts or transcripts that included embedded stops were removed. The resulting groups of sequences were aligned with PRANK (version 170427) using the codon model (*80*). The alignments were then analyzed using codeml from the PAML package (version 4.10.6) using the nested models of M1 and M2a (*81*). The M1 model tests for neutral evolution, while the M2A model tests for positive selection of the foreground branch. The foreground branch was set as Jonathan, and the background contained *H. sapiens*, *Heterocephalus glaber*, *Anolis carolinensis*, *Pelodiscus sinensis*, *M. musculus*, *Nothobranchius furzeri*, *Danio rerio*, *Chrysemys picta bellii*, *Gopherus agassizii*, and *Balaena mysticetus*. Next, data were imported into R (version 3.6.3) (*82*). The likelihood ratio test was performed between the nested models with two degrees of freedom, with the resulting values converted to *P* values using R’s pchisq function. PSGs were tested for overrepresentation in GeneAge and LongevityMap using Fisher’s exact test (fisher.test) in R. Results were considered significant if *P* < 0.05.

#### GO term enrichment analysis of PSGs

GO term enrichment analysis was performed using topGO with genes with a ω > 1 for each genome (*83*). For each set, three statistical tests were run: Fisher’s exact test with the classic algorithm, Kolmogorov-Smirnov test with a classic algorithm, and Kolmogorv-Smirnov with the elimination algorithm using the complete human gene set as the background. The resulting tables were then ordered by the Kolmogorv-Smirnov test with the elimination algorithm *P* scores.

### DNA methylation

#### Differentially methylated regions

We generated hypomethylated regions using the “hmr” command in DNM tools and identified DMRs using the “dmr” command. Briefly, a proportion table was generated as described in the DNM tool documentation for differential methylation detection using the “radmeth” command (*84*). Next, we created a design matrix, which was used along with the proportion table to create a bed file of methylated read counts. Next, “radadjust” was used to adjust the *P* values of individual CpGs, and then “radmerge” was run with a *P* value of 0.01, which joins neighboring differentially methylated sites with a *P* value below 0.01. Custom scripts were used to detect whether a DMR overlapped a gene or promoter region.

#### Methylation entropy

Total genome ME was computed using Metheor’s “me” command (*85*). Custom scripts were used to detect whether an entropy region overlapped a gene or promoter region.

#### AlphaFold2 structure prediction

AlphaFold structure prediction was carried out with the Phenix PredictModel tool, which uses AlphaFold2 and the mmseqs2 MSA server and scripts derived from ColabFold (*36*, *86*–*89*). The PredictModel tool uses AlphaFold2 directly for sequences of up to 1500 residues. Longer sequences are split into overlapping chunks of 600 in length with an overlap of 200. These chunks are run individually and then reassembled at the end. To improve the contacts between domains in different chunks, a contact map is created, and domains that are in contact but in different chunks are repredicted together. During assembly, the domain pairs are used to adjust the positions of domains, and also, positions of domains are adjusted to minimize overlaps. The repositioned domains are then used as a template to morph the fully reassembled prediction. The result of this procedure is a full-length prediction from overlapping chunk predictions, with distortions (largely in low-confidence regions) that allow the assembled prediction to minimize overlaps and make domain-domain contacts predicted to be present between chunks.

#### Network analysis

The FASTA file with all protein sequences from Jonathan was uploaded to the STRING web interface as a custom proteome (*24*). We subsequently retrieved the functional association network for the PSGs at a 0.4 confidence cutoff using Cytoscape stringApp and clustered it using the clusterMaker2 implementation of the Markov cluster algorithm (*90*, *91*). We lastly highlighted genes belonging to the set of conserved PSGs, GeneAge, or LongevityMap using Omics Visualizer and manually annotated selected clusters with descriptive labels (*92*).

#### Structural analyses

The AlphaFold2-predicted models were processed with the Phenix Process–predicted model tool to remove the low-confidence regions (LDDT <0.7). Pairs of the processed models of the Lonesome George and Jonathan proteins are superposed and analyzed with the ProSMART program to identify backbone and side-chain differences between the two protein pairs (*93*). Residues with their main-chain or side-chain deviations greater than 3 standard deviations of all the corresponding values of the analyzed residues are flagged for visual inspection, especially for those corresponding to key mutations of proteins related to Jonathan’s extended life span.

#### Gene family expansion

To detect gene family expansion, we created protein clusters using protein similarities computed by DIAMOND, as previously described using “grouping.py” and “gen_table.py” available on GitHub (*79*). The clusters were created with a protein coverage cutoff of 20%, a match length cutoff of 20%, and an expected value threshold of 1 × 10^−100^, with at least 50% identity, ensuring that proteins in the cluster were not coded by DNA sequences whose genomic locations overlapped. These clusters were then reduced to a table of non-overlapping genes, indicating possible duplication. A specific subset of candidate gene family expansions of interest was then manually curated.

### Export permit: EMDEP020/17

We received an export permit for Jonathan’s samples from the St. Helena government (Department of Environment & Natural Resources Directorate) on 8 April 2017.

### Institutional Animal Care and Use Committee approval

We received approval for buccal scrap collections from Aldabras from the Vanderbilt University Medical Center and Vanderbilt University Institutional Animal Care and Use Committee.

### Gemini Advanced 2.0

Using Google’s Gemini Advanced 2.0, sections of the original manuscript with a length of 4393 words were placed into Gemini, and we asked it to reduce the word count by ∼30%. The paired-down version was then edited to maintain the essential information and ideas and to avoid hallucinations.

## Supplementary Materials

### The PDF file includes:

Supplementary Text Figs. S1 to S13 Legends for tables S1 to S9 References
