# Supplementary material for: Epigenetic insights into extreme longevity in the world’s oldest terrestrial animal, Jonathan
Source: Sci Adv. 2026 Oct 7;12(41):eadw8887. doi: 10.1126/sciadv.adw8887 (PMC13644754; doi:10.1126/sciadv.adw8887)
Supplement: Supplementary file 1 [file sciadv.adw8887_sm.pdf]

Science Advances
AAAS

Supplementary Materials for

Epigenetic insights into extreme longevity in the world's oldest terrestrial animal, Jonathan

Benjamin Vaisvil *et al.*

Corresponding author: Stephen W. Clark, sclark@kallel.org

Sci. Adv. **12**, eadw8887 (2026)
DOI: 10.1126/sciadv.adw8887

The PDF file includes:

Supplementary Text
Figs. S1 to S13
Legends for tables S1 to S9
References

Other Supplementary Material for this manuscript includes the following:

Tables S1 to S9

Supplementary Text

Jonathan's estimated age

Although Jonathan's exact age is unknown, we estimate his age to be at least 192 based on the following work.

Information about the existence of giant tortoises on St. Helena comes from the work of Dr. Arthur Loveridge. Dr. Loveridge was a Professor of Biology at Harvard University and joined its Museum of Comparative Zoology in 1924 as Curator of Herpetology until 1957 (94). Upon his retirement, he moved to St. Helena and contributed several articles on the wildlife on the island. He cites two bodies of work in his published articles in the island's *Wirebird* magazine: Work by Dr. Albert Gunther, who states the Island had a persistent occupation of giant tortoises preceding Napoleon's exile (1815-1821), with the first arriving via the Mauritius island in 1776 (95). He cites "Plantation Notes" from St. Helena, where a letter was found from St. Helena's governor Grey Wilson (1890-1897) addressed to Captain P. Oliver and dated 10/28/1894, stating that a fully grown male giant tortoise was imported to the island in 1882 (95). After this male arrived, only it and a female existed on the island until the female died in 1918. Her body was examined and was found to contain eggs. The lone existing male, in 1918, would later be named Jonathan.

An old photograph of Jonathan and another tortoise has been circulating in the news for at least ten years, with a publication in 2014 by the BBC and again in 2016 by the Daily Mail (**fig. S1A**) (96,97). The BBC article claimed the photograph was from an 1882 celebration of Jonathan's arrival. Jonathan can be identified in this picture because he has a unique marking on one of his scutes, which is apparent in recent photographs of Jonathan and his veterinarian Joe Hollins (**fig. S1B**). This photograph was examined by Geoffrey Batchen, professor of the History of Art at Oxford University, with special expertise in the history of photography. Dr. Batchen believes this photograph is an albumen print from a glass collodion negative and was likely taken between 1850 and 1900. The earliest record of this photograph identified was a postcard dated December 1907 (**fig. S1C**) (98). If we assume this photograph was taken in 1907, then Jonathan is at least 167 years old; however, we believe, as detailed below, he is much older.

After the Daily Mail published this photograph in 2016, an English lady named Rosemary Rees contacted the island to inform them that her paternal grandfather had the same photograph in his possession at the time of his death in 1886 (**fig. S2A**). According to Mrs. Rees, her grandfather lived at Bedford in the Cape Colony of South Africa, where he was a medical officer aboard the Walmer and Dublin Castle ship, which frequently called upon St. Helena. On the back of her photograph, there is a stamp that reads: "A.L. Innes, Jamestown, St. Helena Studio" (**fig. S2B**). In addition, there was a description of Jonathan's dimensions that matched his current measurements, indicating that at the time of the photograph he was fully grown. In addition, we found a collection of photographs at Christie's auction house, auctioned

on April 8, 2004 (**fig. S2C**) (99). The collection is labeled "St. Helena, 1889-1890 – An album of 76 photographs of St. Helena by B. Wood, B. Grant, and others, printed by A.L. Innes, St. Helena Studio, Jamestown…". We have not determined if the photograph of Jonathan is part of this collection, as Christie's reached out to the buyer in 2024 without a reply, but this establishes that "A.L. Innes" was printing photographs at least as early as 1889. Regardless, we believe the above evidence is sufficient to estimate that the photograph was taken, likely in celebration of Jonathan's arrival on the island, between 1882 and 1886.

A paucity of data limits our understanding of Aldabra shell growth rates. Dr. P. Grubb, of the Department of Zoology, University of Ghana, mostly completed data relating carapace length to age. Dr. Grubb took measurements from the Aldabra atoll at three locations: Cinq and Takamaka locations on Grande Terre island, and one location on Malabar island (9). The range of ages in this cohort was from one to 31 years. In this study, a carapace length (**fig. S3A**) was measured on each tortoise, and a presumed age was determined by counting the number of annuli present in one scute (**fig. S3B**). The scutes are bony units that cover and comprise the tortoise shell, and the annuli are growth rings within the scutes (100). Data from Dr. Gubb's paper used the annuli to determine the age of the tortoises. However, he noted that beyond 25 annuli, it becomes difficult to count as the annuli get compressed together. Thus, for this reason, the ages of the Aldabras were all less than 32. A total of 368 Aldabras were measured from Grand Terre with ages ranging from one to 24, and 58 Aldabras were measured from Malabar with ages 12 to 31 (**fig. S4**) (9). The longest carapace length found was 95 cm from a 30-year-old Malabar Aldabra, and the largest standard deviation in carapace length was 10 cm, found within a 12-year-old group from Grande-Terre.

Next, from the photograph containing Jonathan (**Fig. S5)**, we estimated his carapace length. Based on historical data, we assumed the gentleman standing closest to Jonathan was 66.8 inches, or approximately 170.18 cm (101). From the photograph of Jonathan and the use of a photo measuring technique, we determined Jonathan's carapace length is 0.65 that of the gentleman's height (**fig. S5**) (102). Thus, we calculate Jonathan's carapace length to be approximately 110 cm. Jonathan's current carapace length is also 110 cm, implying he was fully grown in the photograph. Based on our work regarding Jonathan's biogeography (**Fig. 1B-D**), we believe Jonathan likely arose from the Grand Terre island of the Aldabra atoll, as he is more proximal to this island than Malabar. Using linear regression of Dr. Grubb's data, we produced three plots (size vs age) for all sites of measurements (**fig. S4**). We extrapolated from these plots and, based on the size of 110 cm, Jonathan is predicted to be 45 with the Takamaka data, 62.2 from the Cinq data, and 46 from the Malabar data. If we average the two Grand Terre sites (Takamaka and Cinq) we get an average age of 53.6, and if we average all three sites (Takamaka, Cinq, and Malabar), we get an average age of 51.1 years. From the residual plots, we can tell that growth is not continuous and thus, a linear regression plot does not faithfully represent the data and likely underestimates his age when he is 110 cm, as growth rates are declining in the longer animals. We think the differences in the three sites are likely due to population density and access to food. This data is limited in three ways: first, annuli were used to estimate age,

even though Dr. Grubb states that beyond 25 annuli it becomes difficult to count the annuli (four animals from Malabar are estimated to be older than 25), secondly, several of the "longer" specimens only had a measurement of one individual and finally, these measurements were made in 1967 and 1968. Since Jonathan was likely present on one of these islands 136 years earlier, the population density and food access at that time were likely different. Thus, we conservatively estimate it took him 50 years to reach 110 cm, and we conservatively estimate he was 50 years old in the Jonathan photograph. If he is 50 in the Jonathan photograph and it was taken in 1882, Jonathan's birthdate would be approximately 1832, and his current age would be 192. However, Jonathan could be older than 50 in the photograph, and his current age could be older than 192.

In conclusion, Jonathan's exact age cannot be determined with absolute certainty using these data. Recent work in epigenetics, especially DNA methylation, has produced epigenetic clocks that closely estimate chronological age. For instance, DNA methylation data in bats has been used to construct an epigenetic clock that can accurately predict their chronological age (103). If an accurate epigenetic clock can be generated for Aldabras, it could be used to obtain a more reliable estimate of Jonathan's age.

Insertions and deletions

Lastly, we examined our gene set for insertions and deletions. Ten genes had one amino-acid change (*ATRX*, *FOSL2*, *FUS*, *HLA-DRB1*, *HNRNPD*, *IFNL3*, *IGF1R*, *LY9*, *PPP1R26*, and *TDG*), seven with insertions, and three with deletions (*HNRNPD*, *LY9*, and *TDG*)(**table S9**). Interestingly, previous work on Lonesome George found that in both him and the Aldabra, a single amino acid deletion was identified in IGF2R and a variant was found in IGF1R, both of which were verified (6). Thus, Jonathan has alterations in both *IGF1R* and *IGF2R*.

Fig. S1

A

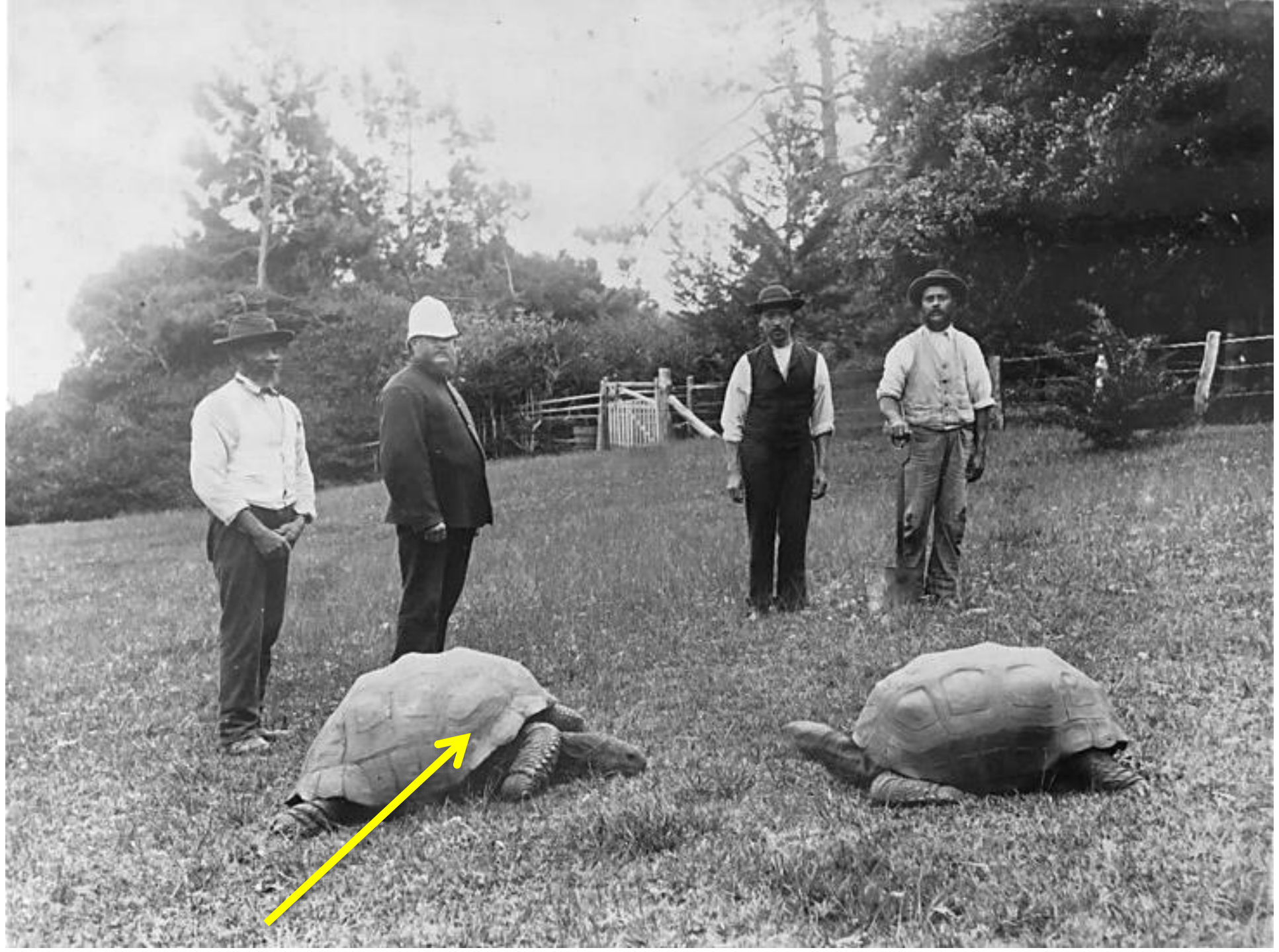

B

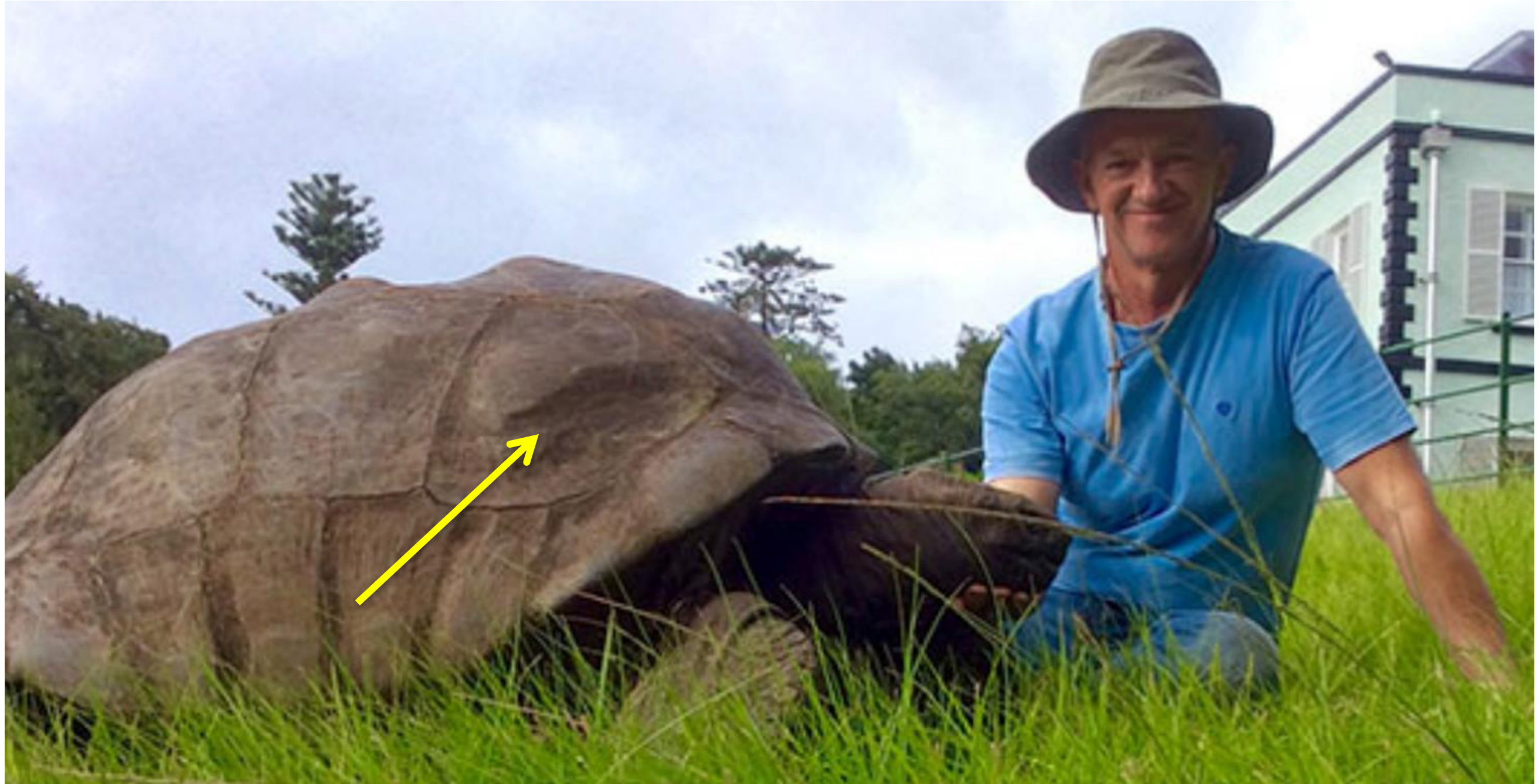

C

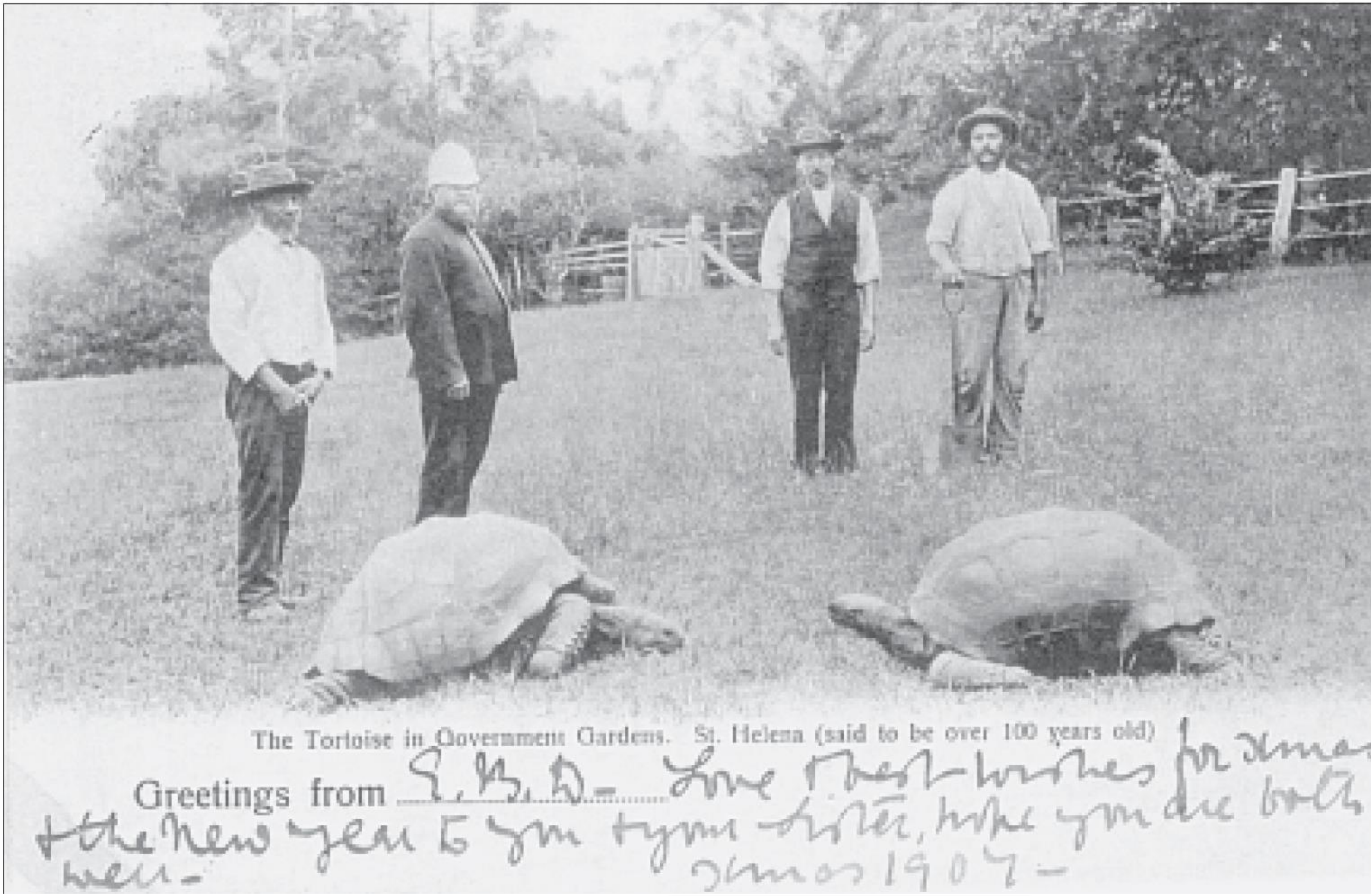
The Tortoise in Government Gardens. St. Helena (said to be over 100 years old)
Greetings from E. B. D. Love & best wishes for Xmas & the New Year to you & your sister, hope you are both well -
Xmas 1907 -

Fig. S1. Jonathan's photograph. A. The photograph shows two giant tortoises, with Jonathan being on the left. The yellow arrow points to a unique scute on Jonathan's shell. The gentleman behind Jonathan is considered Helenian based on the writing on the back of Rosemary Rees's copy. The location of the photograph is at the corner of Plantation Paddock, near the public gate to the tortoise corridor on the island of St. Helena. We believe this photograph was taken between 1882 and 1886 and likely in 1882 (Photo Credit: Photographer A.L. Innes. Image in the public domain). **B.** A more recent photograph of Jonathan and his vet, Joe Hollins. The yellow arrow points to a unique scute on Jonathan's shell as in 1a. The location of the photograph is the grounds of the Plantation House, the official residence of the governor of St. Helena. **C.** A postcard reproducing the Jonathan photograph. The date on the postcard is "Xmas 1907", which would be sometime in December of 1907[7] (Photo Credit: Photographer A.L. Innes (1882). Image courtesy of [www.tpa-project.info]. Image in the public domain.).

Fig. S2

A

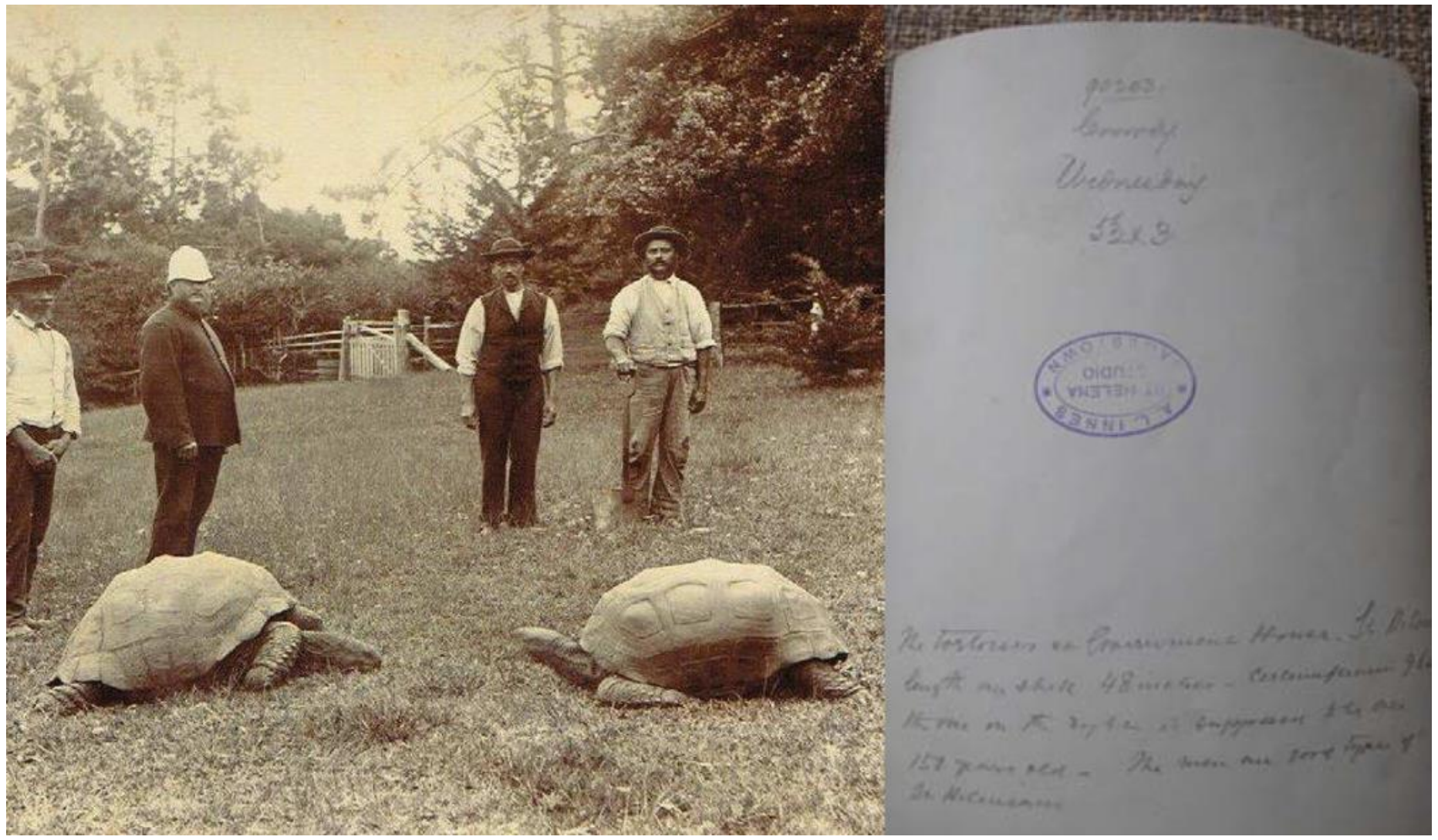

B

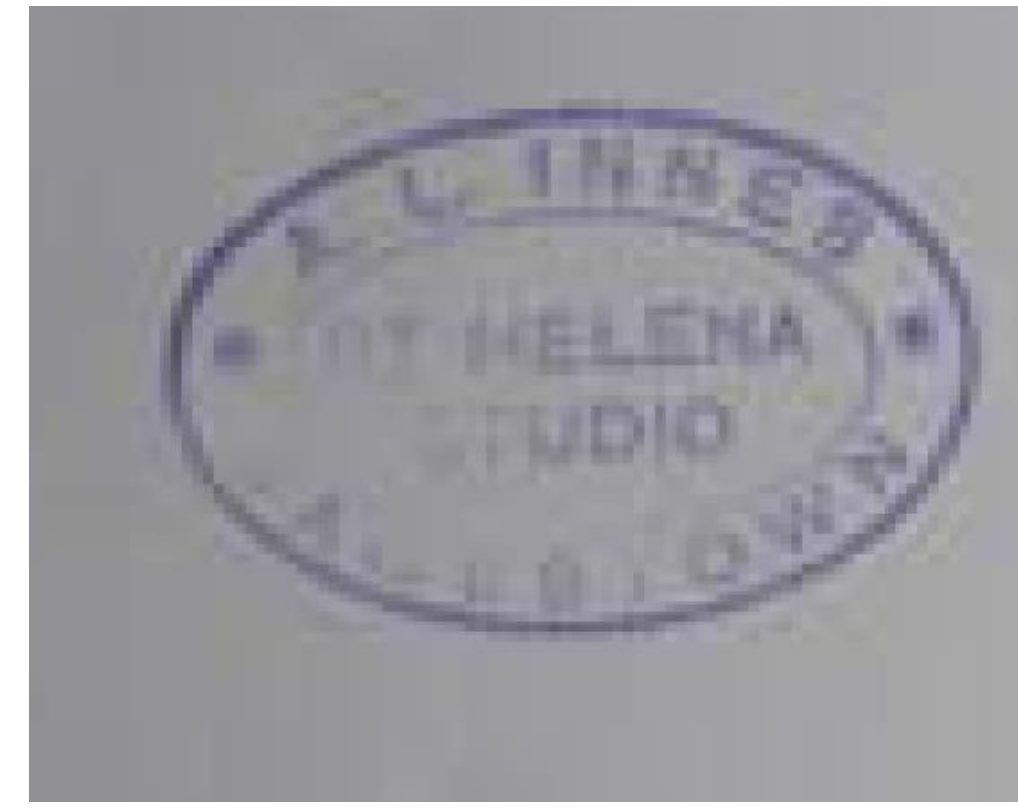
A.L. INNES
ST HELENA
STUDIO

C

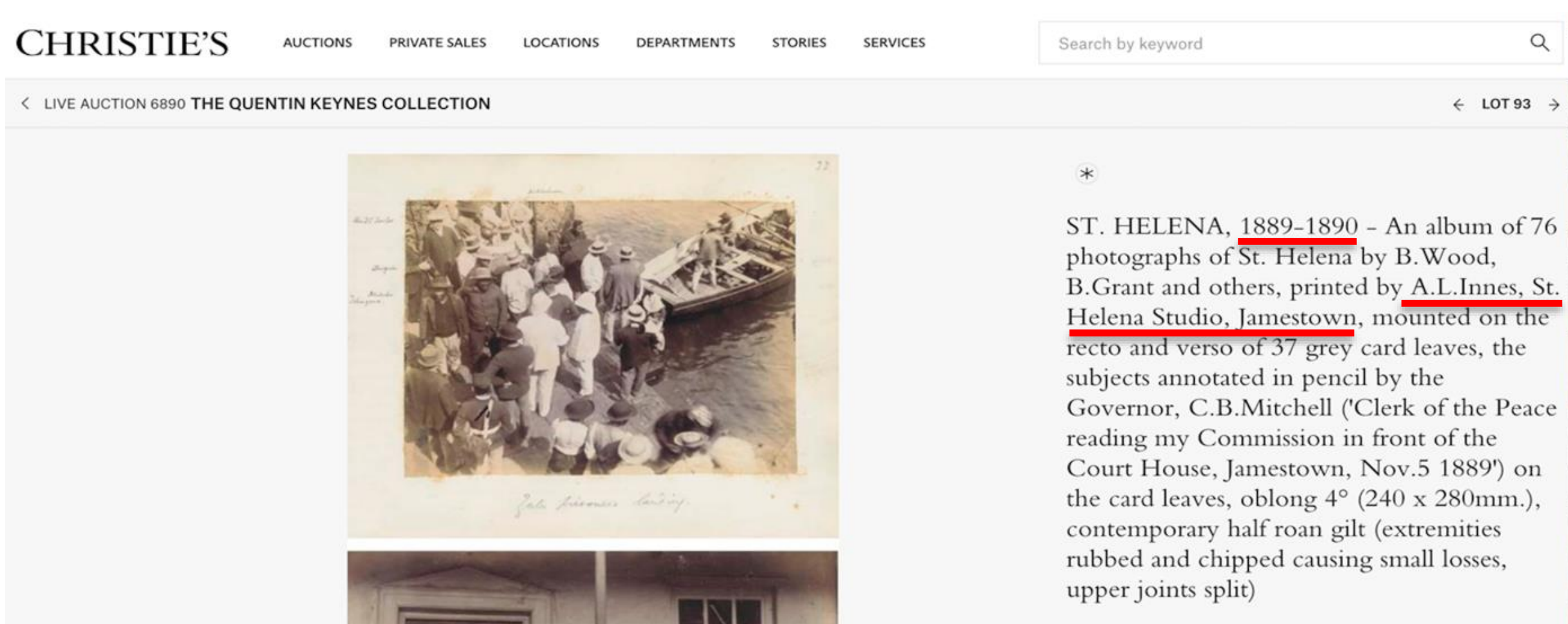
CHRISTIE'S
AUCTIONS
PRIVATE SALES
LOCATIONS
DEPARTMENTS
STORIES
SERVICES
Search by keyword
LIVE AUCTION 6890 THE QUENTIN KEYNES COLLECTION
LOT 93
ST. HELENA, 1889-1890 - An album of 76 photographs of St. Helena by B.Wood, B.Grant and others, printed by A.L.Innes, St. Helena Studio, Jamestown, mounted on the recto and verso of 37 grey card leaves, the subjects annotated in pencil by the Governor, C.B.Mitchell ('Clerk of the Peace reading my Commission in front of the Court House, Jamestown, Nov.5 1889') on the card leaves, oblong 4° (240 x 280mm.), contemporary half roan gilt (extremities rubbed and chipped causing small losses, upper joints split)

Fig. S2. Determining the age of Jonathan's photograph. A. The copy of the Jonathan photograph, presented by Rosemary Rees. The back of the photograph is shown as well with the printer's stamp and the writing on her copy. **B.** A close-up of the printer's stamp is shown as in "a". The top reads "A.L. INNES", the bottom reads "JAMESTOWN", and in the center of the stamp reads "ST. HELENA STUDIOS". **C.** The Christie's auction web page depicts "THE QUENTIN KEYNES COLLECTION, Lot 93". The red underlined portion of the collection description highlights the date "1889-1890" of the collection and that it was printed by "A.L. Innes, St. Helena Studio, Jamestown"[8].

Fig. S3

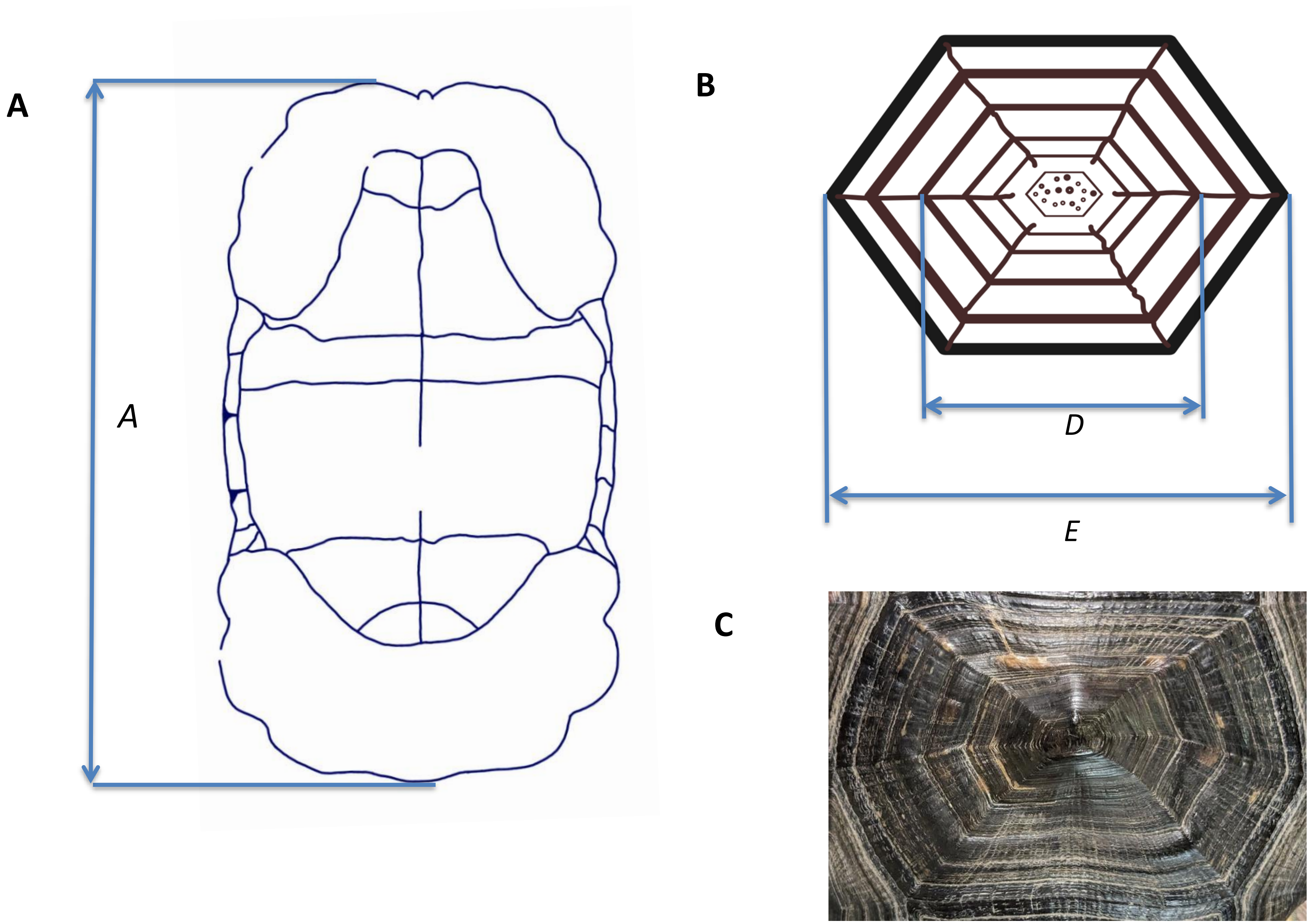

Fig. S3. Carapace length and scute size with annulus. A. A reproduction of “Figure 1” in Dr. Grubbs's paper[9]. The left figure depicts a ventral view of the Aldabra shell, with “A” being the carapace length. **b.** A dorsal view of a scute, with “D” being the fourth annulus (4th year) of the scute and “E” being the width of the scute. **c.** Photograph of the 3rd dorsal scute of an Aldabra.

Fig. S4

Grande Terre

Takamaka | Cinq | **Malabar**

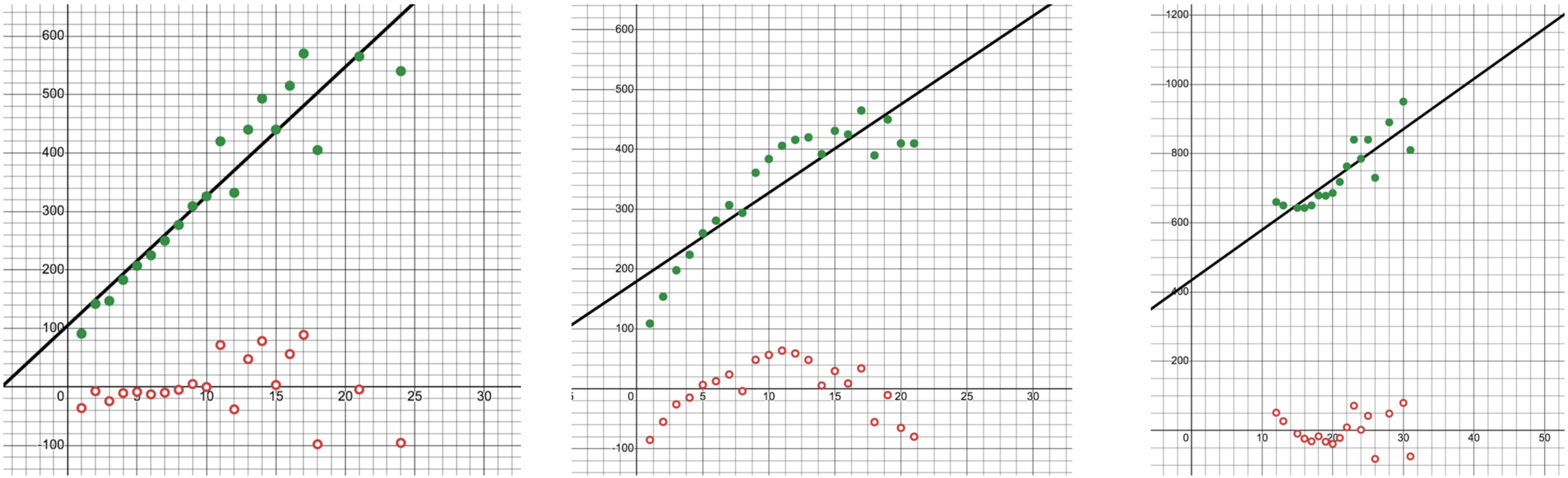

[y-axis: mean carapace length (mm); x-axis: age (years)]

Takamaka

Predicted age
for 110 cm = 45y

EQUATION

$y = 22.1122x + 105.0382$

STATISTICS

$R^2 = 0.8912$

$r = 0.944$

RESIDUALS

e_1

Cinq

Predicted age
for 110 cm = 62.2y

EQUATION

$y = 14.78182x + 179.6381$

STATISTICS

$R^2 = 0.7947$

$r = 0.8915$

RESIDUALS

e_1

Malabar

Predicted age
for 110 cm = 46y

EQUATION

$y = 14.54419x + 434.0642$

STATISTICS

$R^2 = 0.7517$

$r = 0.867$

RESIDUALS

e_1

Average of the three sites and two islands is 51.1 years

Fig. S4. Replotting of Dr. Grubb's growth data for Aldabra on the Aldabra atoll. A replot of Dr. Grubb's data, with the x-axis representing the carapace length in millimeters (mm) and the y-axis representing the "presumed age" in years of the tortoises[9]. The age was calculated by counting the number of annuli of a scute. Three separate populations are plotted, one population from Malabar and two populations from Grande Terre (Takamaka and Cinq). Based on linear regression, a 110 cm carapace length tortoise would have an age of 45 years old with the Takamaka data, 62.2 years old with the Cinq data, and 46 years old with the data from Malabar. The average of these populations gives a predicted age of 51.3 years. Open red dots = residuals. A residual in linear regression is the difference in the actual value and the value predicted by the regression line at that point and equals the observed value minus the predicted value.

Fig. S5

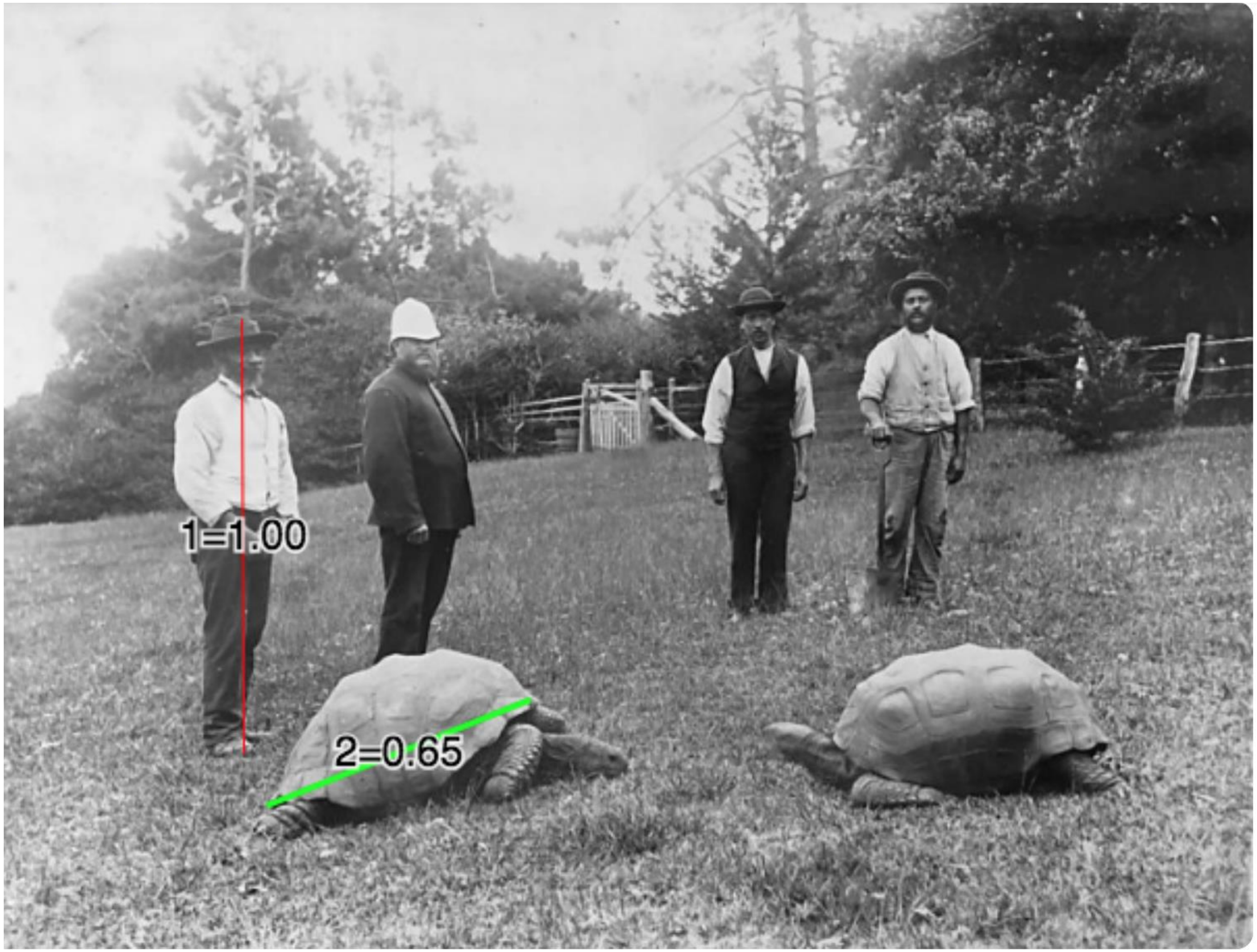

Fig. S5. Carapace length of Jonathan in Jonathan's photograph. The Jonathan photograph is shown with markings used to measure his carapace length. The closest person to Jonathan is set as 1.0 (red markings) and then Jonathan's carapace measurement is determined by a photo measuring technique to be 0.65 in relation to the human measurement[12]. Based on historical data, we assumed the gentleman standing closest to Jonathan was 66.8 inches or approximately 170.18 cm[11], and based on this, we calculated Jonathan's carapace length to be 110 cm.

Fig. S6

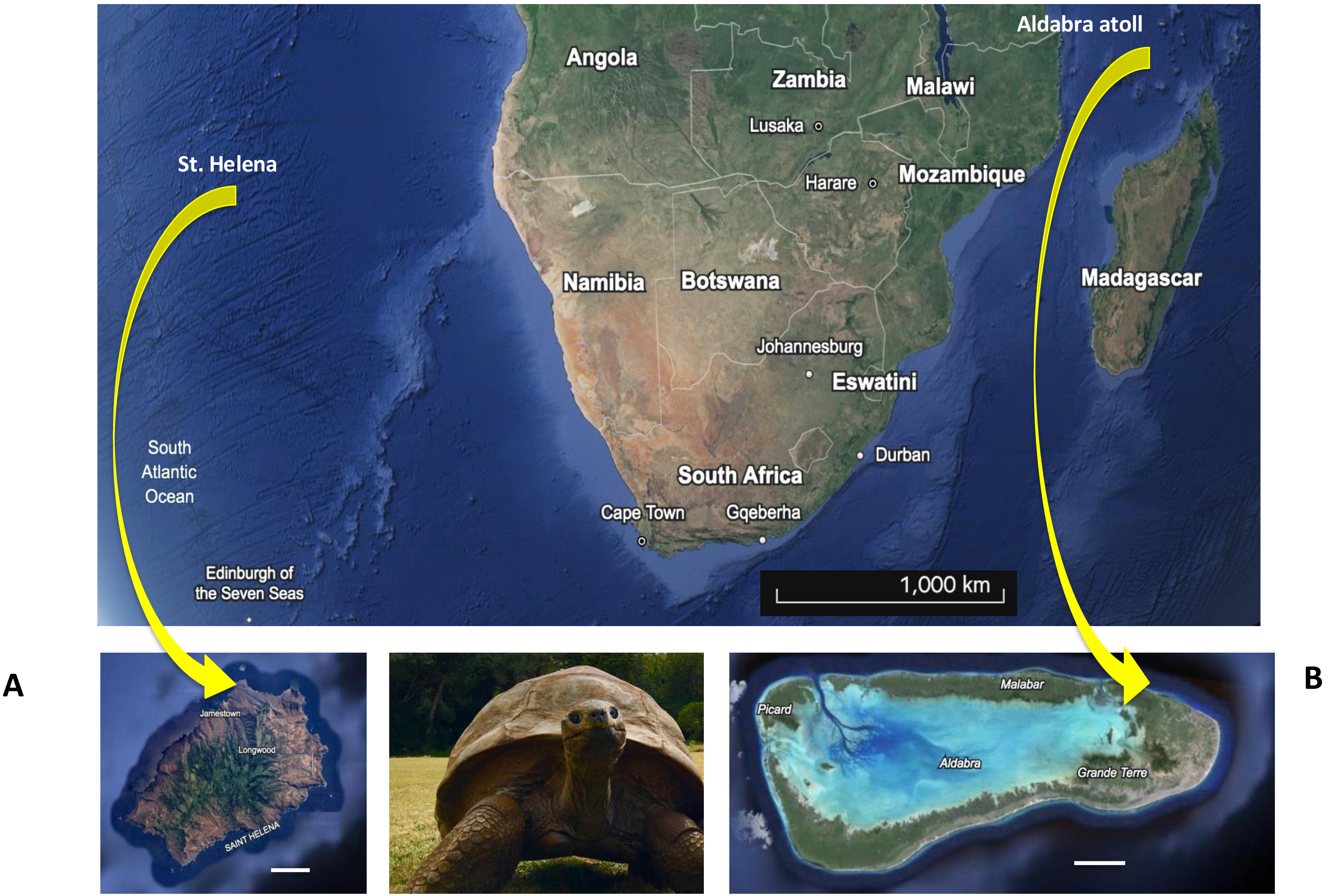

St. Helena Jonathan Aldabra atoll

Fig. S6. Geographic location of St. Helena and Aldabra Atoll.
A. Location of St. Helena in the South Atlantic Ocean. **B.** Location of the Aldabra Atoll in the Indian Ocean. Maps generated from Google Earth. Bar = 1000 km, White Bars = 3.2 km.

Fig. S7

A

Assembly Metrics

	Tank		Jonathan
**Metric**	**Contigs**	**Scaffolds**	**AlternateFasta**
Number	193	213	213
Total bases (bp)	2,413,091,811	2,413,097,311	2,412,858,454
N50 length (bp)	113,433,846	138,737,524	138,728,499
N50 Count	7	6	6
N90 length (bp)	24,900,843	24,936,114	24,934,970
N90 Count	26	20	20
N (%)	0	0	0
GC (%)	44	44	44

Jonathan's Annotation Metrics

Metric	Gene	CDS
Total	25,738	31,416
Annotated	16,466	20,877
Mean Length	29,671	550
Max Length	778,565	23,879
Median Length	12,054	382

B

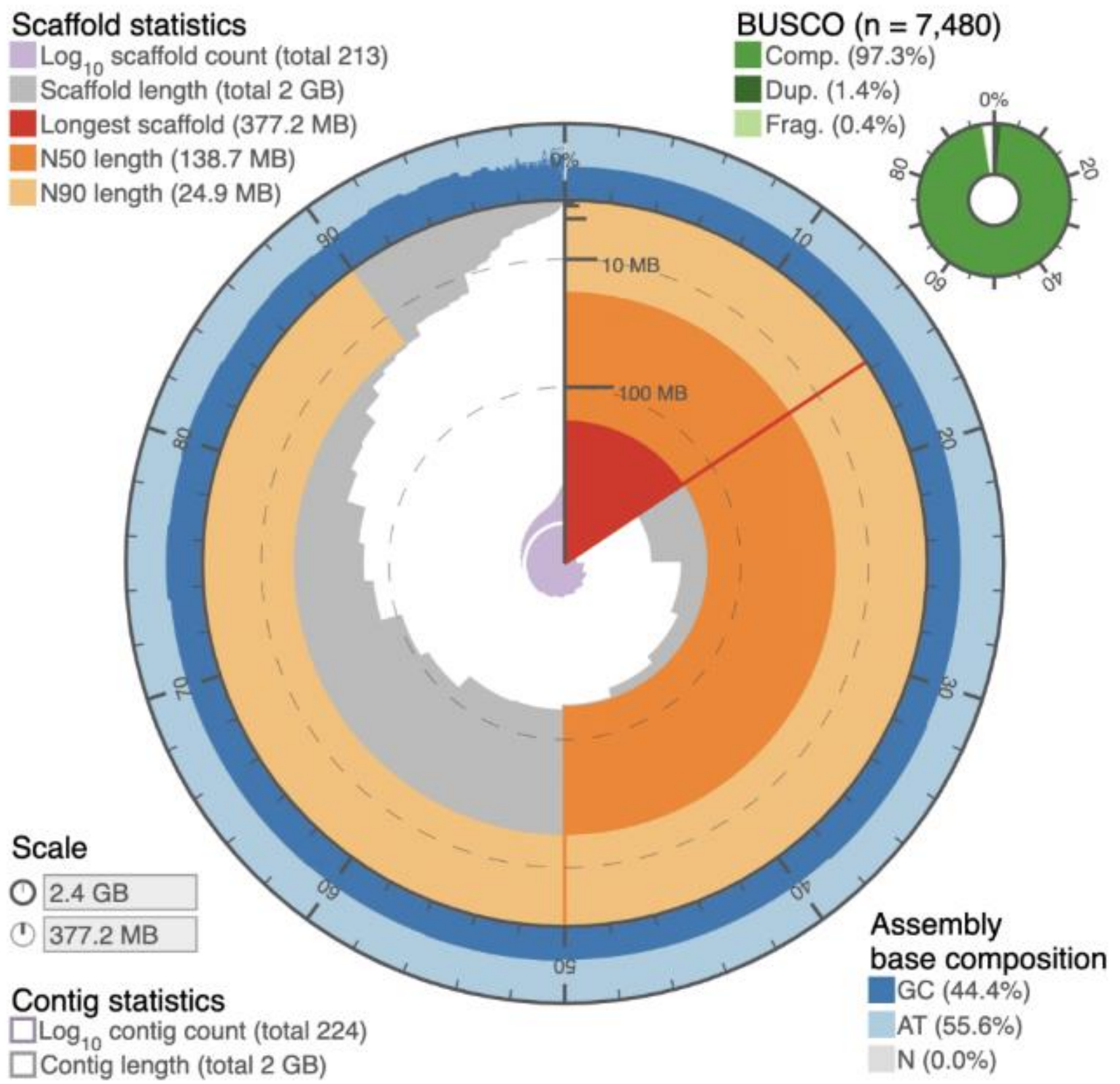

Fig. S7. The *de novo* sequencing and assembly of the reference genome of Tank and the consensus genome of Jonathan. **A**. Assembly statistics of the reference genome of Jonathan and Tank. **B**. Assembly metrics, sequence composition, and BUSCO completeness scores for the Sauropsida dataset and Tank. The number of annotated coding and non-coding genes in Tank and Jonathan's genomes.

Fig. S8

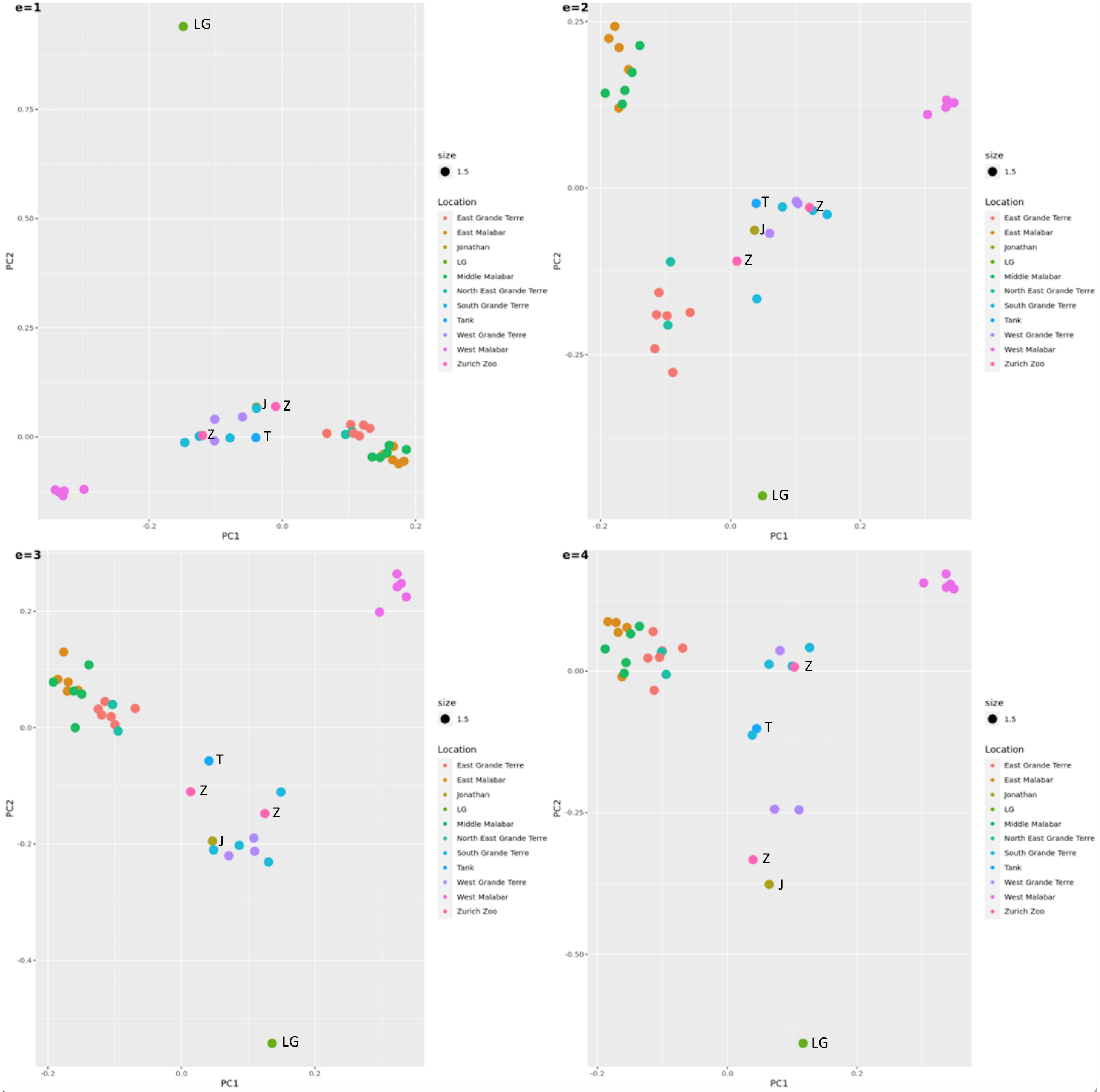
e=1
e=2
e=3
e=4
PC1
PC2
size
1.5
Location
East Grande Terre
East Malabar
Jonathan
LG
Middle Malabar
North East Grande Terre
South Grande Terre
Tank
West Grande Terre
West Malabar
Zurich Zoo
LG
J
Z
T

Fig. S8. **Lonesome George's location in principal component plot.** Principal component analysis plot of 32 *Aldabrachelys gigantea* previously plotted from the Islands of Malabar and Grande Terre of the Aldabra atoll. Included are the two Zurich Zoo Aldabras (Z), Tank (T), and Jonathan (J), and as a negative control, Lonesome George (LG). e =1-4.

Fig. S9

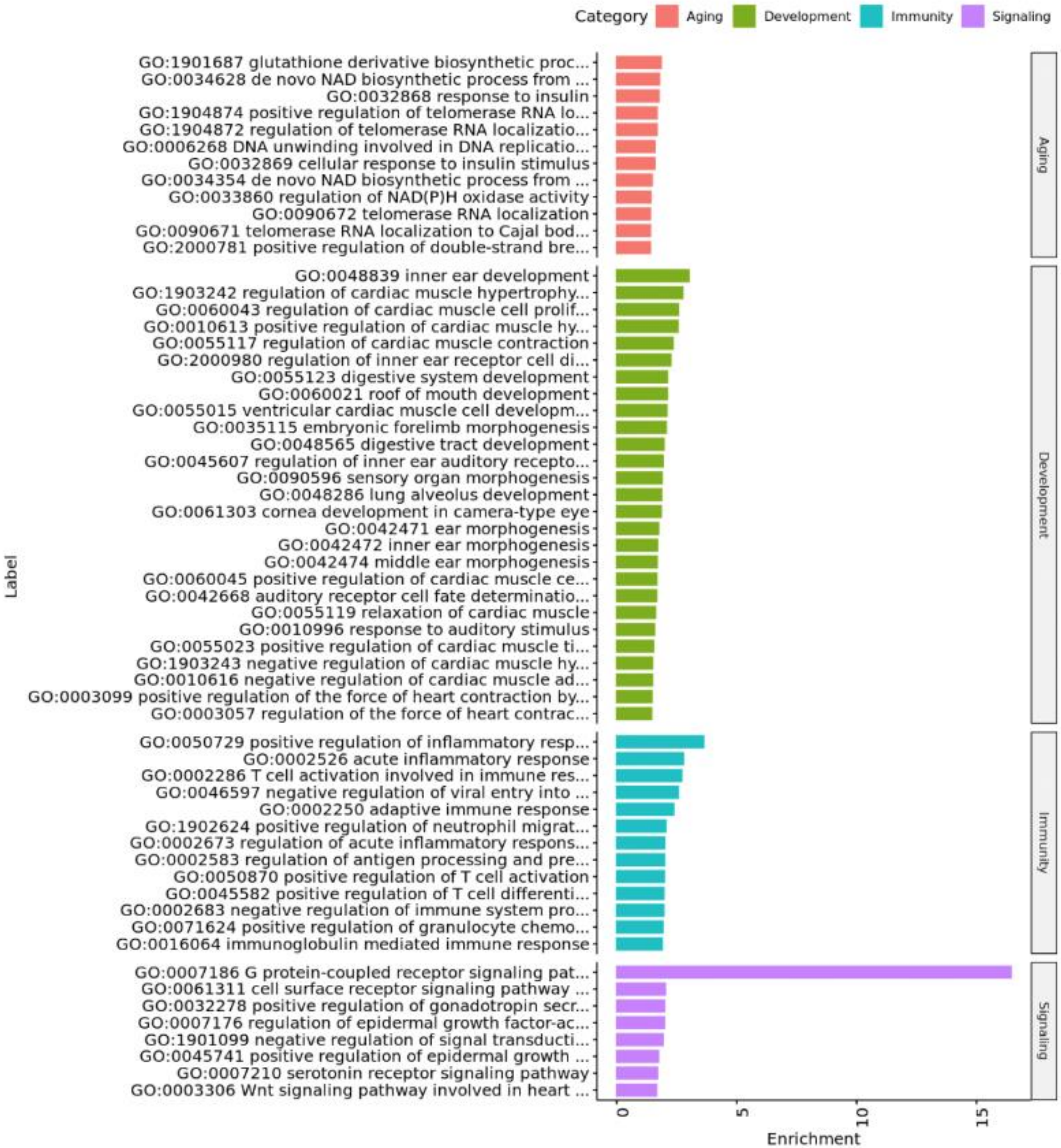
Category Aging Development Immunity Signaling
GO:1901687 glutathione derivative biosynthetic proc...
GO:0034628 de novo NAD biosynthetic process from ...
GO:0032868 response to insulin
GO:1904874 positive regulation of telomerase RNA lo...
GO:1904872 regulation of telomerase RNA localizatio...
GO:0006268 DNA unwinding involved in DNA replicatio...
GO:0032869 cellular response to insulin stimulus
GO:0034354 de novo NAD biosynthetic process from ...
GO:0033860 regulation of NAD(P)H oxidase activity
GO:0090672 telomerase RNA localization
GO:0090671 telomerase RNA localization to Cajal bod...
GO:2000781 positive regulation of double-strand bre...
GO:0048839 inner ear development
GO:1903242 regulation of cardiac muscle hypertrophy...
GO:0060043 regulation of cardiac muscle cell prolif...
GO:0010613 positive regulation of cardiac muscle hy...
GO:0055117 regulation of cardiac muscle contraction
GO:2000980 regulation of inner ear receptor cell di...
GO:0055123 digestive system development
GO:0060021 roof of mouth development
GO:0055015 ventricular cardiac muscle cell developm...
GO:0035115 embryonic forelimb morphogenesis
GO:0048565 digestive tract development
GO:0045607 regulation of inner ear auditory recepto...
GO:0090596 sensory organ morphogenesis
GO:0048286 lung alveolus development
GO:0061303 cornea development in camera-type eye
GO:0042471 ear morphogenesis
GO:0042472 inner ear morphogenesis
GO:0042474 middle ear morphogenesis
GO:0060045 positive regulation of cardiac muscle ce...
GO:0042668 auditory receptor cell fate determinatio...
GO:0055119 relaxation of cardiac muscle
GO:0010996 response to auditory stimulus
GO:0055023 positive regulation of cardiac muscle ti...
GO:1903243 negative regulation of cardiac muscle hy...
GO:0010616 negative regulation of cardiac muscle ad...
GO:0003099 positive regulation of the force of heart contraction by...
GO:0003057 regulation of the force of heart contrac...
GO:0050729 positive regulation of inflammatory resp...
GO:0002526 acute inflammatory response
GO:0002286 T cell activation involved in immune res...
GO:0046597 negative regulation of viral entry into ...
GO:0002250 adaptive immune response
GO:1902624 positive regulation of neutrophil migrat...
GO:0002673 regulation of acute inflammatory respons...
GO:0002583 regulation of antigen processing and pre...
GO:0050870 positive regulation of T cell activation
GO:0045582 positive regulation of T cell differenti...
GO:0002683 negative regulation of immune system pro...
GO:0071624 positive regulation of granulocyte chemo...
GO:0016064 immunoglobulin mediated immune response
GO:0007186 G protein-coupled receptor signaling pat...
GO:0061311 cell surface receptor signaling pathway ...
GO:0032278 positive regulation of gonadotropin secr...
GO:0007176 regulation of epidermal growth factor-ac...
GO:1901099 negative regulation of signal transducti...
GO:0045741 positive regulation of epidermal growth ...
GO:0007210 serotonin receptor signaling pathway
GO:0003306 Wnt signaling pathway involved in heart ...
Label
Aging
Development
Immunity
Signaling
0
5
10
15
Enrichment

Fig. S9. GO analysis of Jonathan's positively selected genes. Categories include aging, development, immunity, and signaling.

Fig. S10

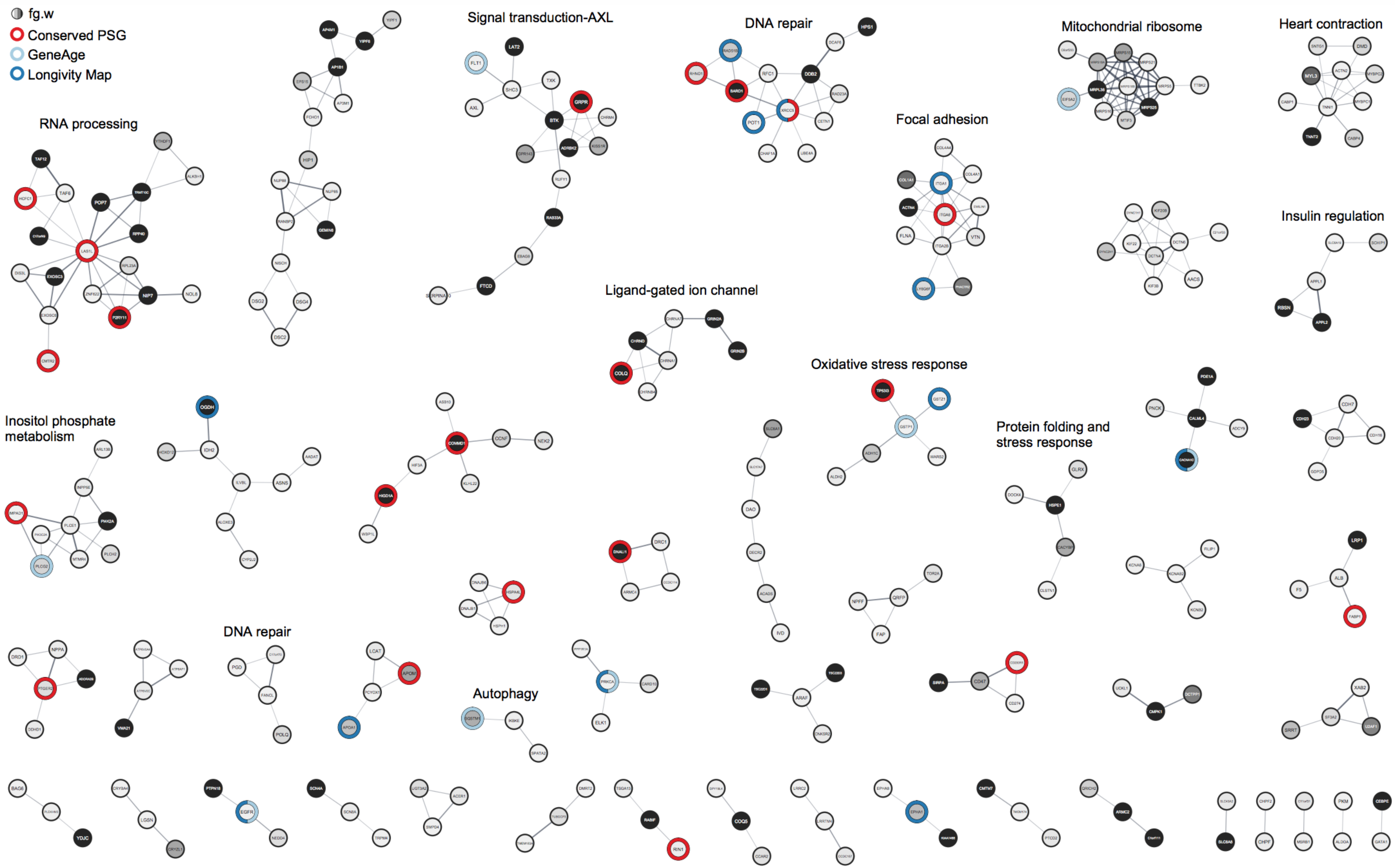
fg.w
Conserved PSG
GeneAge
Longivity Map
RNA processing
Signal transduction-AXL
DNA repair
Mitochondrial ribosome
Heart contraction
Focal adhesion
Insulin regulation
Ligand-gated ion channel
Oxidative stress response
Inositol phosphate metabolism
Protein folding and stress response
DNA repair
Autophagy

Fig. S10. STRING analysis of Jonathan's positively selected genes. Using the STRING algorithm, protein-protein connectivity networks were identified. Listed are networks of interest.

Fig. S11

XRCC6 (p.K556R, TORT = Jono, CABP = LG)

A

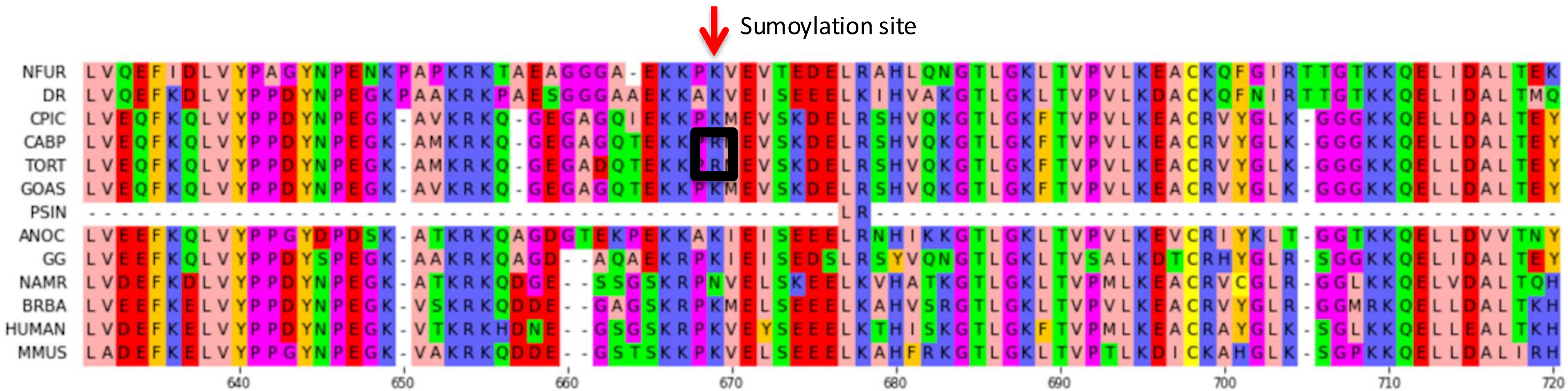

DCLRE1B (p.R498C, TORT = Jono, CABP = LG)

B

Relevant residues for TERF2 interaction

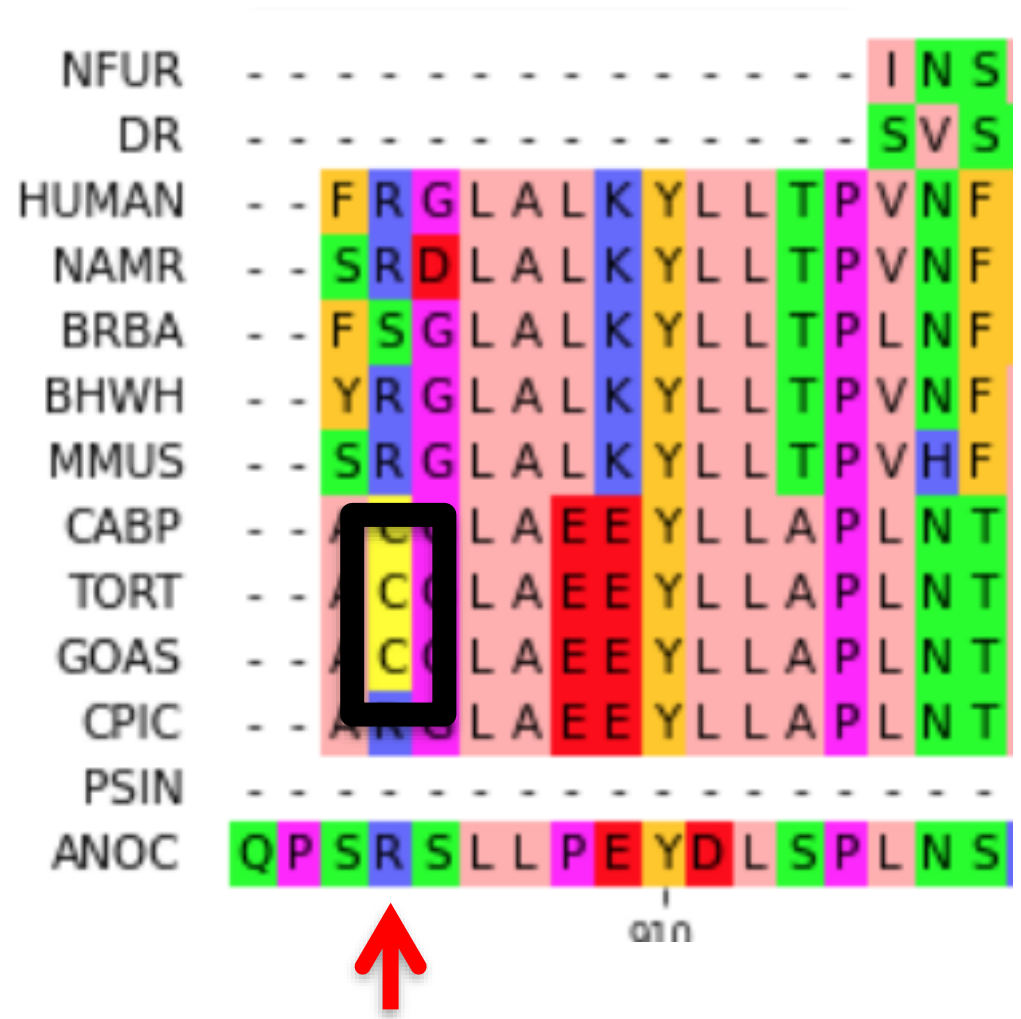

C

IGF1R (p.N724D, TORT = Jono)

Residues important for IGF1/2 interaction

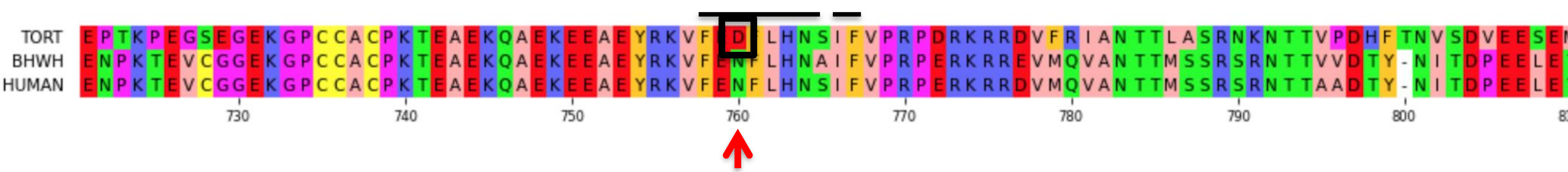

D

ALDH2 (p.M487T, TORT = Jono, CABP = LG)

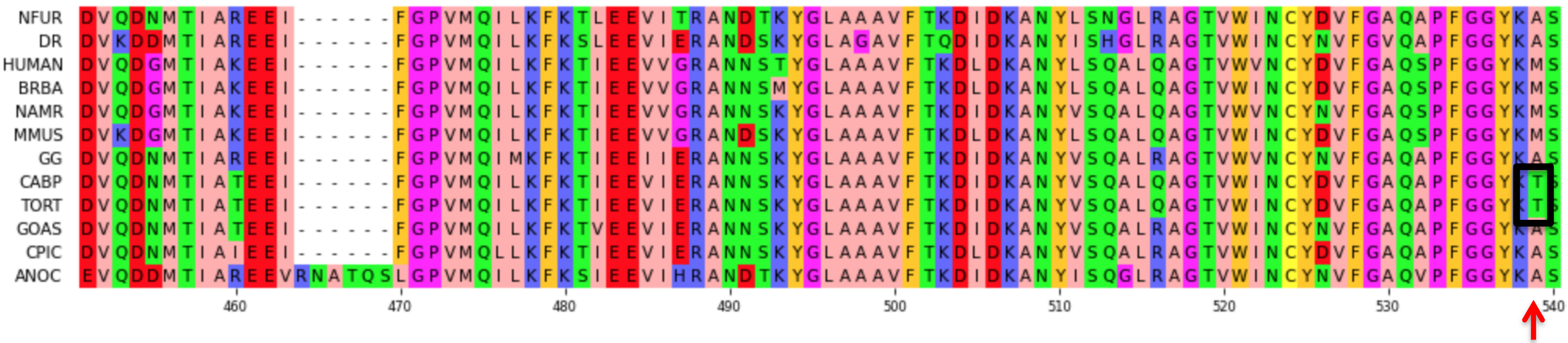

IGF2R (p.Y1758del,TORT = Jono, CABP = LG)

E

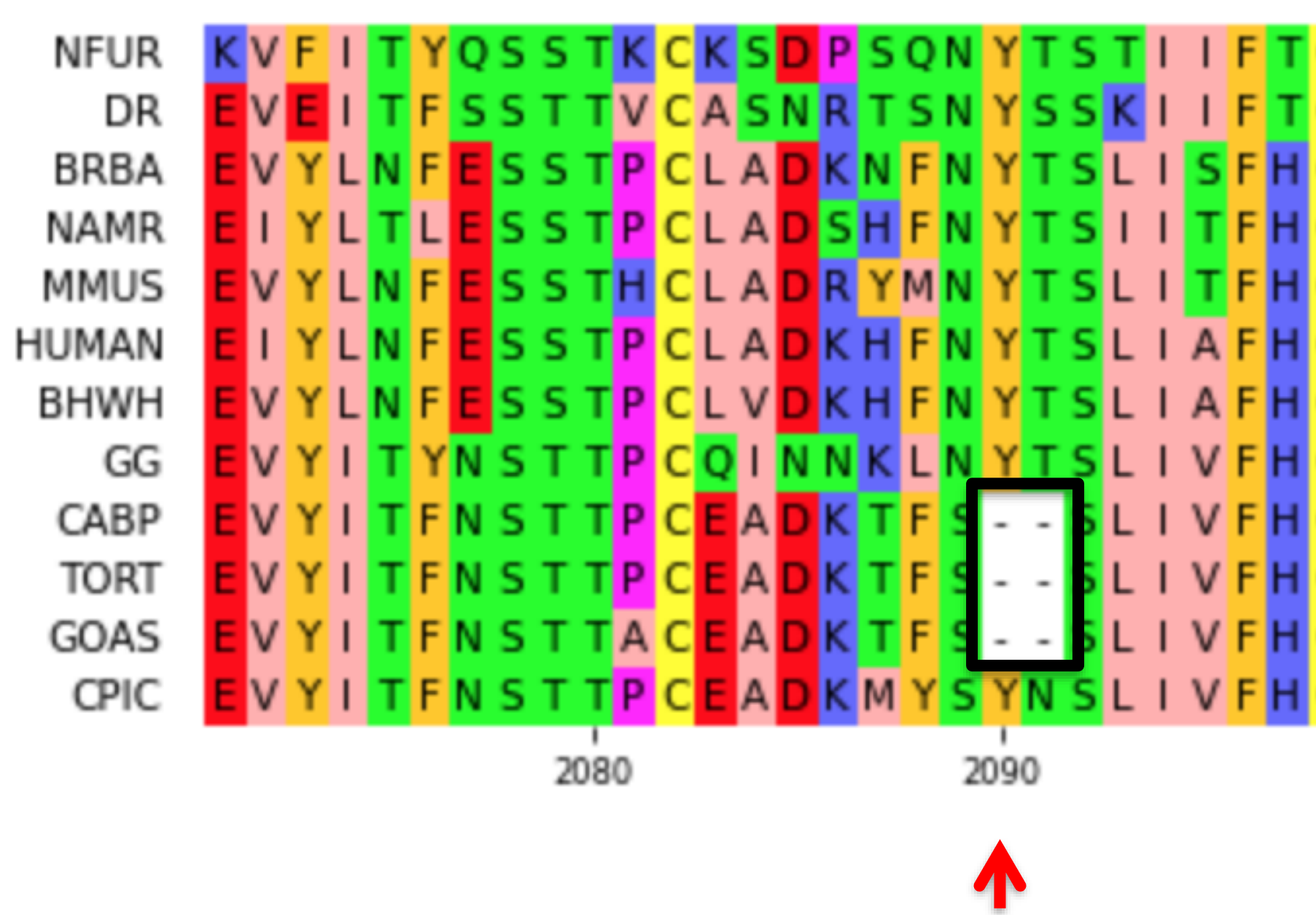

GSK3A (p.R272Q, TORT = Jono, CABP = LG)

F

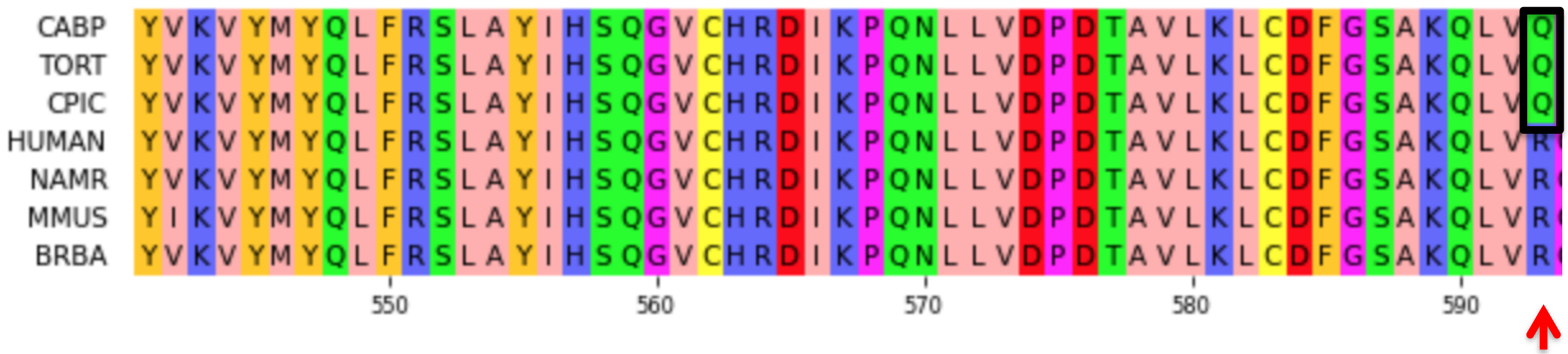

MIF (p.N111C, TORT = Jono, CABP = LG)

G

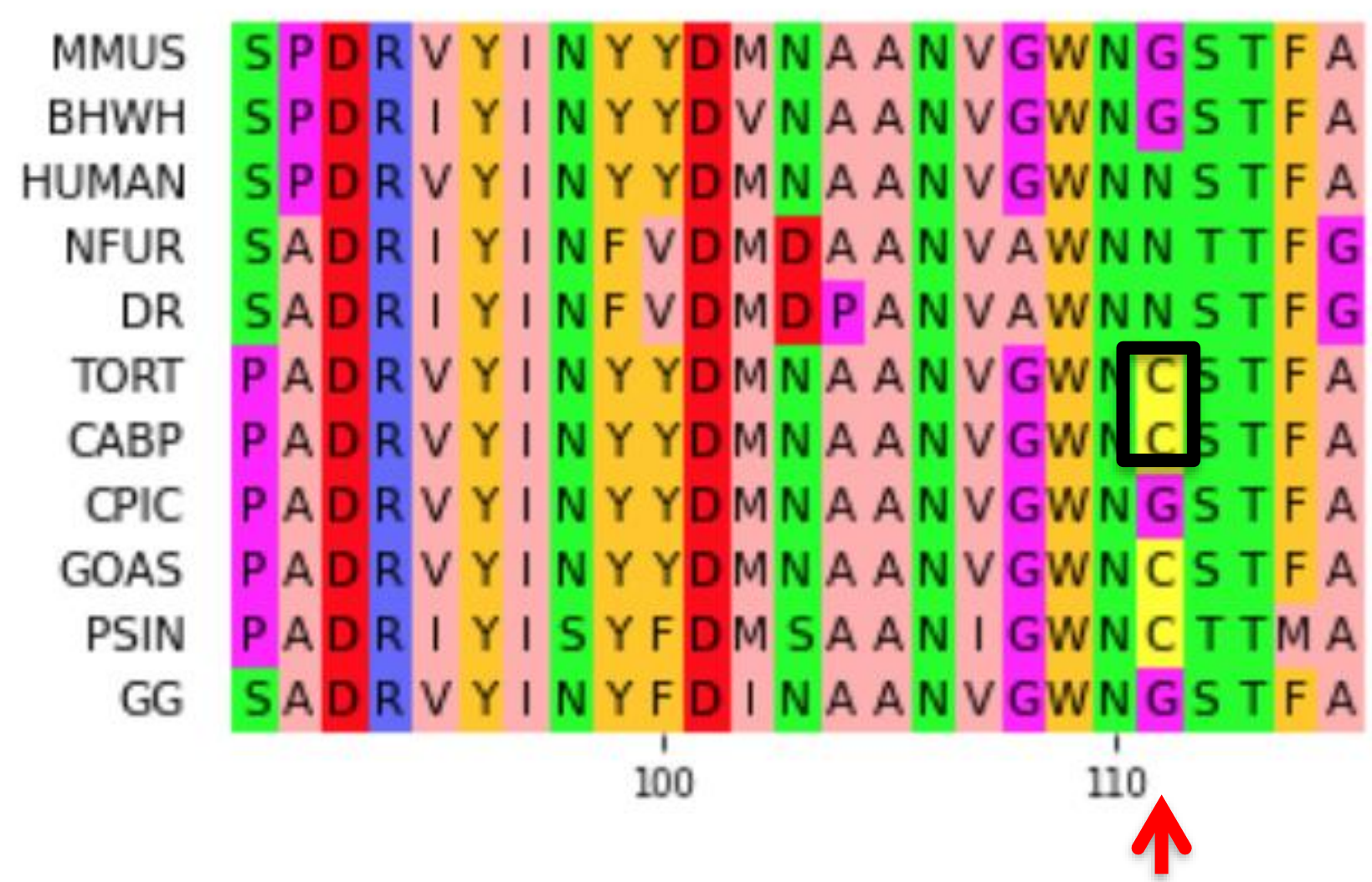

Fig. S11. Variants found in Lonesome George were also found in Jonathan. A. nsNSV in the gene *XRCC6* (K556R). **B.** nsNSV in *DCLRE1B* (R498C) **C.** nsNSV in *IGF1R* (N724D). **D.** nsNSV in *ALDH2* (M487T). **E.** nsNSV in *IGF2R* (Y1758del). **F.** nsNSV in *GSK3A* (R272Q). **G.** nsNSV in *MIF* (N111C). TORT, Jonathan; CABP, Lonesome George; GG, Gallus gallus; NAMR, Heterocephalus glaber (naked mole-rat); BRBA, Myotis brandtii (Brandts bat); ANOC, Anolis carolinensis; PSIN, Peiodiscus sinensis isolate Daiwa-1; MMUS, Mus musculus; NFUR, Nothobranchius fuzeri; DR, Danio rerio (zebrafish), CPIC, Chrysems picta bellii; GOAS, Gopherus agassizii; ALGO, The cilinger aldabra; BHWH, Balaena mysticetus (bowhead whale); RTNK, Tank.

Fig. S12

LAMTOR4

A

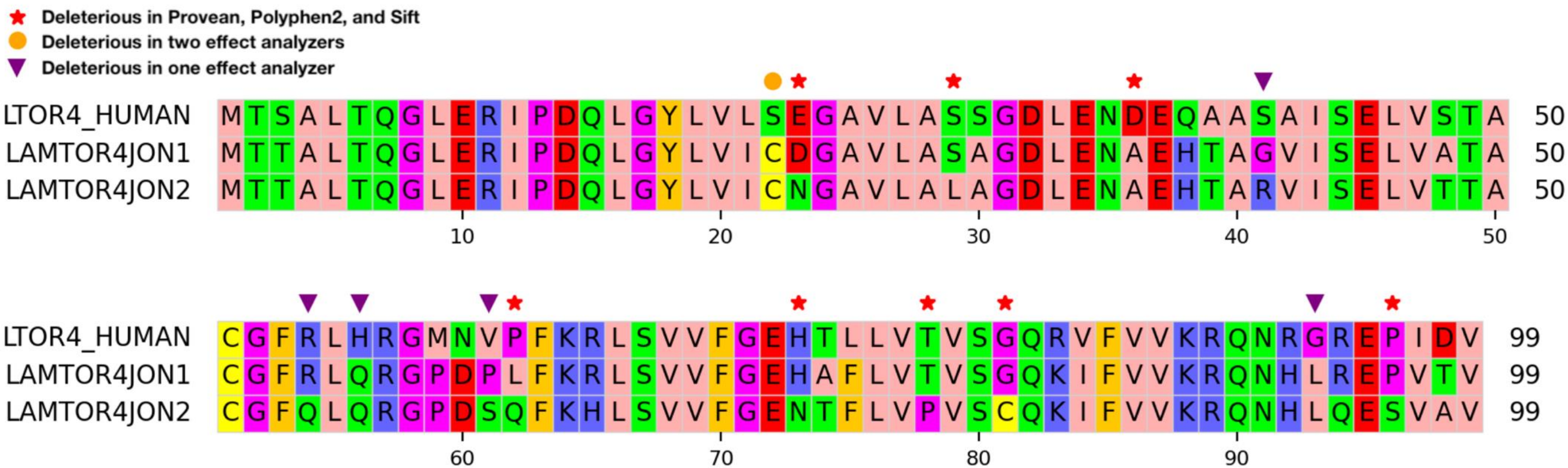

B

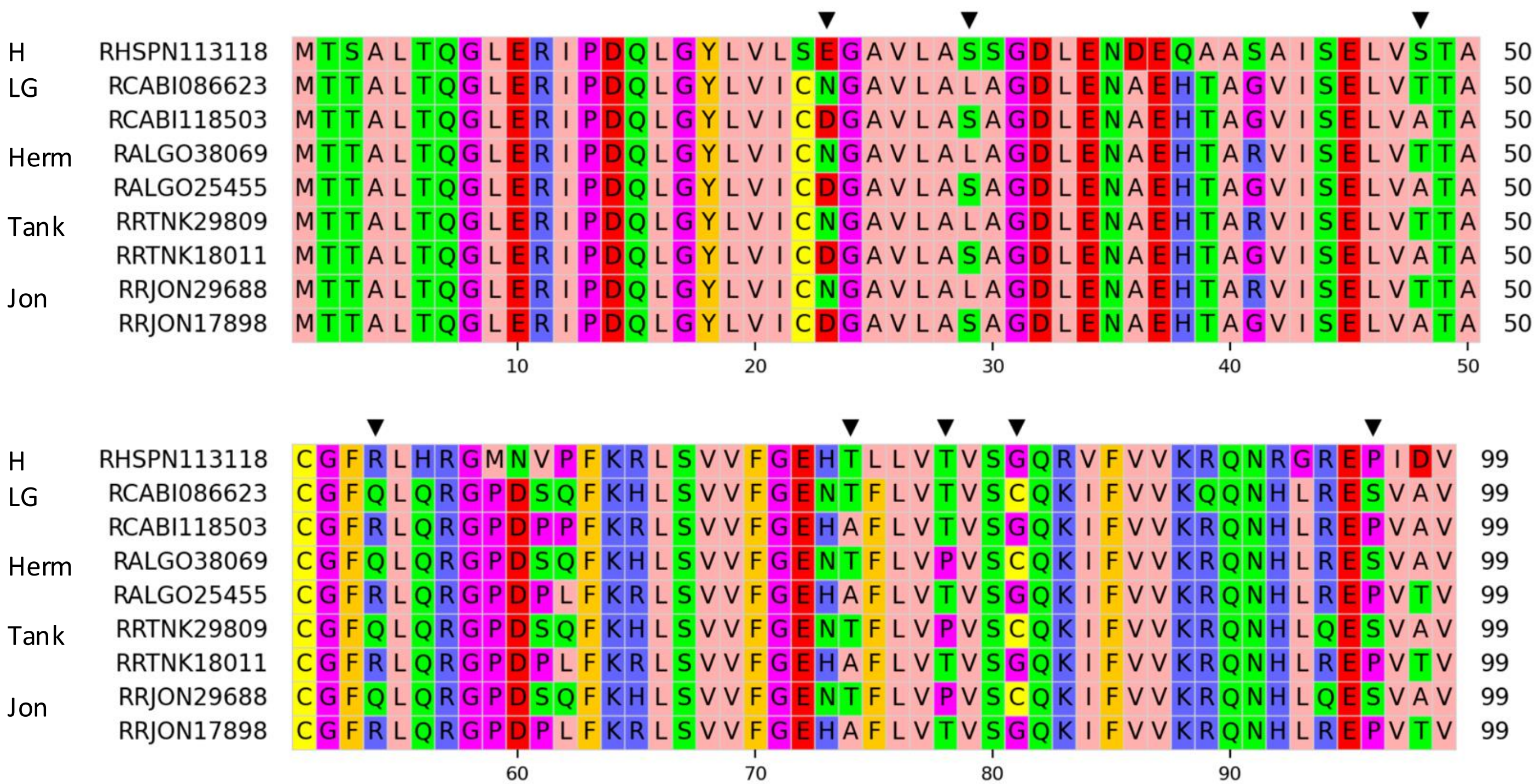

H RHSPN113118 MTSALTQGLERIPDQLGYLVLSEGAVLASSGDLENDEQAASAISELVSTA 50
LG RCABI086623 MTTALTQGLERIPDQLGYLVICNGAVLALAGDLENAEHTAGVISELVTTA 50
RCABI118503 MTTALTQGLERIPDQLGYLVICDGAVLASAGDLENAEHTAGVISELVATA 50
Herm RALGO38069 MTTALTQGLERIPDQLGYLVICNGAVLALAGDLENAEHTARVISELVTTA 50
RALGO25455 MTTALTQGLERIPDQLGYLVICDGAVLASAGDLENAEHTAGVISELVATA 50
Tank RRTNK29809 MTTALTQGLERIPDQLGYLVICNGAVLALAGDLENAEHTARVISELVTTA 50
RRTNK18011 MTTALTQGLERIPDQLGYLVICDGAVLASAGDLENAEHTAGVISELVATA 50
Jon RRJON29688 MTTALTQGLERIPDQLGYLVICNGAVLALAGDLENAEHTARVISELVTTA 50
RRJON17898 MTTALTQGLERIPDQLGYLVICDGAVLASAGDLENAEHTAGVISELVATA 50
10 20 30 40 50
H RHSPN113118 CGFRLHRGMNVPFKRLSVVFGEHTLLVTVSGQRVFVVKRQNRGREPIDV 99
LG RCABI086623 CGFQLQRGPDSQFKHLSVVFGENTFLVTVSCQKIFVVKQQNHLRESVAV 99
RCABI118503 CGFRLQRGPDPPFKRLSVVFGEHAFLVTVSGQKIFVVKRQNHLREPVAV 99
Herm RALGO38069 CGFQLQRGPDSQFKHLSVVFGENTFLVPVSCQKIFVVKRQNHLRESVAV 99
RALGO25455 CGFRLQRGPDPLFKRLSVVFGEHAFLVTVSGQKIFVVKRQNHLREPVTV 99
Tank RRTNK29809 CGFQLQRGPDSQFKHLSVVFGENTFLVPVSCQKIFVVKRQNHLQESVAV 99
RRTNK18011 CGFRLQRGPDPLFKRLSVVFGEHAFLVTVSGQKIFVVKRQNHLREPVTV 99
Jon RRJON29688 CGFQLQRGPDSQFKHLSVVFGENTFLVPVSCQKIFVVKRQNHLQESVAV 99
RRJON17898 CGFRLQRGPDPLFKRLSVVFGEHAFLVTVSGQKIFVVKRQNHLREPVTV 99
60 70 80 90

C

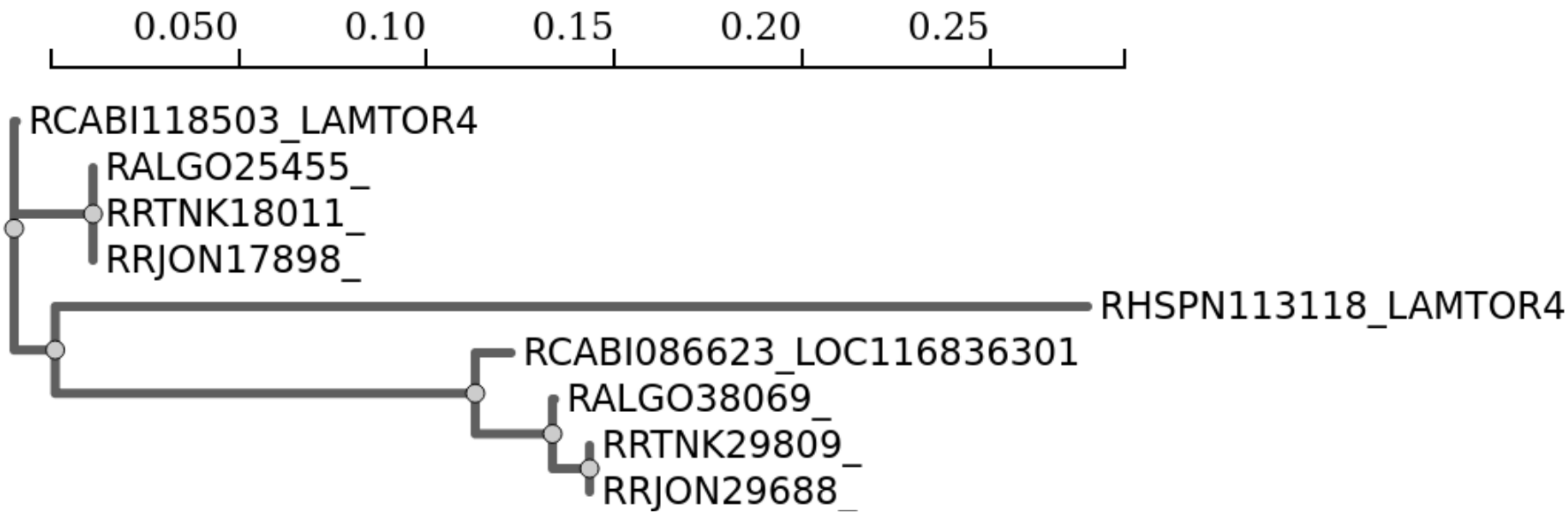

0.050
0.10
0.15
0.20
0.25
RCABI118503_LAMTOR4
RALGO25455_
RRTNK18011_
RRJON17898_
RHSPN113118_LAMTOR4
RCABI086623_LOC116836301
RALGO38069_
RRTNK29809_
RRJON29688_

Fig. S12. Expansion of the *LAMTOR4* gene in giant tortoises. A. Comparison of both copies of LAMTOR4 in Jonathan to a single copy in humans. LAMTOR4JON1, Jonathan LAMTOR4 copy 1; LAMTOR4JON2, Jonathan LAMTOR4 copy 2. nsNSVs in copies were evaluated by protein function algorithms, Provean, Polyphen2, and SIFT. **B.** Comparison of normal copy of LAMTOR4 between human (H), Lonesome George (LG), Hermania (Herm), Tank, and Jon (Jonathan). Comparison of copy #2 in the above tortoises to humans. **C.** Phylogenetic tree relationship between the LAMTOR4 copies (millions of years).

Fig. S13

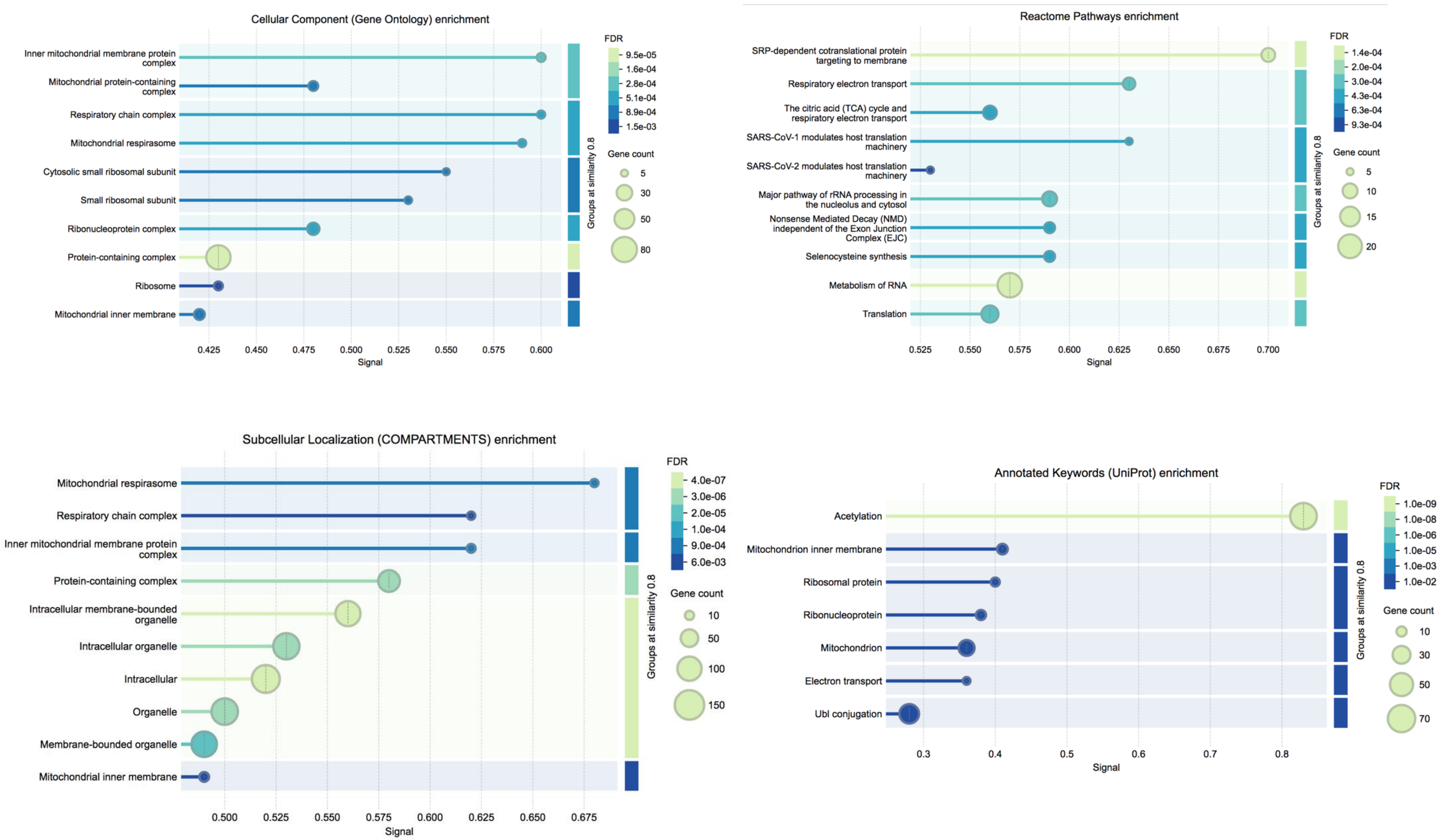

Cellular Component (Gene Ontology) enrichment
Inner mitochondrial membrane protein complex
Mitochondrial protein-containing complex
Respiratory chain complex
Mitochondrial respirasome
Cytosolic small ribosomal subunit
Small ribosomal subunit
Ribonucleoprotein complex
Protein-containing complex
Ribosome
Mitochondrial inner membrane
0.425 0.450 0.475 0.500 0.525 0.550 0.575 0.600
Signal
Groups at similarity 0.8
FDR
9.5e-05 1.6e-04 2.8e-04 5.1e-04 8.9e-04 1.5e-03
Gene count
5 30 50 80
Reactome Pathways enrichment
SRP-dependent cotranslational protein targeting to membrane
Respiratory electron transport
The citric acid (TCA) cycle and respiratory electron transport
SARS-CoV-1 modulates host translation machinery
SARS-CoV-2 modulates host translation machinery
Major pathway of rRNA processing in the nucleolus and cytosol
Nonsense Mediated Decay (NMD) independent of the Exon Junction Complex (EJC)
Selenocysteine synthesis
Metabolism of RNA
Translation
0.525 0.550 0.575 0.600 0.625 0.650 0.675 0.700
Signal
Groups at similarity 0.8
FDR
1.4e-04 2.0e-04 3.0e-04 4.3e-04 6.3e-04 9.3e-04
Gene count
5 10 15 20
Subcellular Localization (COMPARTMENTS) enrichment
Mitochondrial respirasome
Respiratory chain complex
Inner mitochondrial membrane protein complex
Protein-containing complex
Intracellular membrane-bounded organelle
Intracellular organelle
Intracellular
Organelle
Membrane-bounded organelle
Mitochondrial inner membrane
0.500 0.525 0.550 0.575 0.600 0.625 0.650 0.675
Signal
Groups at similarity 0.8
FDR
4.0e-07 3.0e-06 2.0e-05 1.0e-04 9.0e-04 6.0e-03
Gene count
10 50 100 150
Annotated Keywords (UniProt) enrichment
Acetylation
Mitochondrion inner membrane
Ribosomal protein
Ribonucleoprotein
Mitochondrion
Electron transport
Ubl conjugation
0.3 0.4 0.5 0.6 0.7 0.8
Signal
Groups at similarity 0.8
FDR
1.0e-09 1.0e-08 1.0e-06 1.0e-05 1.0e-03 1.0e-02
Gene count
10 30 50 70

Fig. S13. GO analysis of Jonathan's hypermethylated DMRs and elite gene promoters using the STRING database. A-B. The 218 genes whose promoters or coding regions contained hypermethylated DMRs were evaluated with Gene Ontology (GO) enrichment (A, "Biological Process"; B, "WikiPathways"). **C-D.** The 272 gene promoters that are "elite" in that they show low ME in Hatchling, Smalls, and Jonathan but increased ME in Toka and Poka were evaluated with GO. Genes involved in "protein-containing complex" or "intracellular membrane-bound organelle " were enriched for "Biological Process" (C and D, respectively).

Note: Each table is available as a supplementary Excel file.

Table. S1. Jonathan's and Tank's genome assembly metrics. Assembly metrics including repeat analysis of both Jonathan and Tank, metrics of Jonathan's mitochondrial genome, and the location of the samples used for the PCA plot.

Table. S2. Jonathan's positively selected genes (PSGs). Provean and SIFT analysis of each variant. Only genes with omega values greater than one are shown. Jonathan's PSGs shared with other long-lived species and with LongevityMap and GeneAge.

Table. S3. Unique nsNSVs found only in Jonathan. The non-synonymous variants detected in the studied gene set and the nsNSVs located within positively selected genes.

Table. S4. Evaluation of Jonathan's unique nsNSVs by protein language models. Protein Language models predictions (Tranception L no retrieval, ESM1v ensemble, Progen2 base, RITA XL, and VESPA1). Ratio A and Ratio B.

Table. S5. Genes that are found in more than one protein language model. The top genes with nsNSVs determined by the different protein language models from table S4 and their correspondence in LongevityMap, GeneAge, and CellAge.

Table. S6. Gene expansion in Jonathan. Tortoise-specific gene expansions. Comparing Jonathan, Lonesome George, and other tortoises.

Table. S7. Genes within Jonathan's hypermethylated DMRs, and which of these are PRC2 targets. Here we identify which gene promoters and exons are within Jonathan's DMRs and which have been identified as PRC2 targets.

Table. S8. Promoter methylation entropy for all five tortoises. Here we evaluate the promoter methylation entropy of a large gene set within Jonathan and those he shares with the other four tortoises, and identify a subset that remains low in Jonathan compared to Toka and Poka.

Table. S9. Insertions and deletions in Jonathan. Here we identify genes that are inserted or deleted in Jonathan and their position in both the human and Jonathan genomes.

REFERENCES

1. E. B. Kim, X. Fang, A. A. Fushan, Z. Huang, A. V. Lobanov, L. Han, S. M. Marino, X. Sun, A. A. Turanov, P. Yang, S. H. Yim, X. Zhao, M. V. Kasaikina, N. Stoletzki, C. Peng, P. Polak, Z. Xiong, A. Kiezun, Y. Zhu, Y. Chen, G. V. Kryukov, Q. Zhang, L. Peshkin, L. Yang, R. T. Bronson, R. Buffenstein, B. Wang, C. Han, Q. Li, L. Chen, W. Zhao, S. R. Sunyaev, T. J. Park, G. Zhang, J. Wang, V. N. Gladyshev, Genome sequencing reveals insights into physiology and longevity of the naked mole rat. *Nature* **479**, 223–227 (2011).

2. M. Keane, J. Semeiks, A. E. Webb, Y. I. Li, V. Quesada, T. Craig, L. B. Madsen, S. van Dam, D. Brawand, P. I. Marques, P. Michalak, L. Kang, J. Bhak, H.-S. Yim, N. V. Grishin, N. H. Nielsen, M. P. Heide-Jørgensen, E. M. Oziolor, C. W. Matson, G. M. Church, G. W. Stuart, J. C. Patton, J. C. George, R. Suydam, K. Larsen, C. López-Otín, M. J. O’Connell, J. W. Bickham, B. Thomsen, J. P. de Magalhães, Insights into the evolution of longevity from the bowhead whale genome. *Cell Rep.* **10**, 112–122 (2015).

3. I. Seim, X. Fang, Z. Xiong, A. V. Lobanov, Z. Huang, S. Ma, Y. Feng, A. A. Turanov, Y. Zhu, T. L. Lenz, M. V. Gerashchenko, D. Fan, S. Hee Yim, X. Yao, D. Jordan, Y. Xiong, Y. Ma, A. N. Lyapunov, G. Chen, O. I. Kulakova, Y. Sun, S.-G. Lee, R. T. Bronson, A. A. Moskalev, S. R. Sunyaev, G. Zhang, A. Krogh, J. Wang, V. N. Gladyshev, Genome analysis reveals insights into physiology and longevity of the Brandt’s bat *Myotis brandtii*. *Nat. Commun.* **4**, 2212 (2013).

4. D. R. Valenzano, B. A. Benayoun, P. P. Singh, E. Zhang, P. D. Etter, C.-K. Hu, M. Clément-Ziza, D. Willemsen, R. Cui, I. Harel, B. E. Machado, M.-C. Yee, S. C. Sharp, C. D. Bustamante, A. Beyer, E. A. Johnson, A. Brunet, The african turquoise killifish genome provides insights into evolution and genetic architecture of lifespan. *Cell* **163**, 1539–1554 (2015).

5. M. Pascual-Torner, D. Carrero, J. G. Pérez-Silva, D. Álvarez-Puente, D. Roiz-Valle, G. Bretones, D. Rodríguez, D. Maeso, E. Mateo-González, Y. Español, G. Mariño, J. L. Acuña, V. Quesada, C. López-Otín, Comparative genomics of mortal and immortal cnidarians unveils novel keys behind rejuvenation. *Proc. Natl. Acad. Sci. U.S.A.* **119**, e2118763119 (2022).

6. V. Quesada, S. Freitas-Rodríguez, J. Miller, J. G. Pérez-Silva, Z.-F. Jiang, W. Tapia, O. Santiago-Fernández, D. Campos-Iglesias, L. F. K. Kuderna, M. Quinzin, M. G. Álvarez, D. Carrero, L. B. Beheregaray, J. P. Gibbs, Y. Chiari, S. Glaberman, C. Ciofi, M. Araujo-Voces, P. Mayoral, J. R. Arango, I. Tamargo-Gómez, D. Roiz-Valle, M. Pascual-Torner, B. R. Evans, D. L. Edwards, R. C. Garrick, M. A. Russello, N. Poulakakis, S. J. Gaughran, D. O. Rueda, G. Bretones, T. Marquès-Bonet, K. P. White, A. Caccone, C. López-Otín, Giant tortoise genomes provide insights into longevity and age-related disease. *Nat. Ecol. Evol.* **3**, 87–95 (2019).

7. F. G. Çilingir, L. A'Bear, D. Hansen, L. R. Davis, N. Bunbury, A. Ozgul, D. Croll, C. Grossen, Chromosome-level genome assembly for the Aldabra giant tortoise enables insights into the genetic health of a threatened population. *GigaScience* **11**, giac090 (2022).

8. R. da Silva, D. A. Conde, A. Baudisch, F. Colchero, Slow and negligible senescence among testudines challenges evolutionary theories of senescence. *Science* **376**, 1466–1470 (2022).

9. P. Grubb, The growth, ecology, and population of structure of giant tortoises on Aldabra. *Philos. Trans. R. Soc. B* **260**, 327–372 (1971).

10. D. Bourn, M. J. Coe, Features of tortoise mortality and decomposition on Aldabra. *Philos. Trans. R. Soc. B* **286**, 189–193 (1979).

11. C. Kehlmaier, E. Graciá, J. R. Ali, P. D. Campbell, S. D. Chapman, V. Deepak, F. Ihlow, N.-E. Jalil, L. Pierre-Huyet, K. E. Samonds, M. Vences, U. Fritz, Ancient DNA elucidates the lost world of western Indian Ocean giant tortoises and reveals a new extinct species from Madagascar. *Sci. Adv.* **9**, eabq2574 (2023).

12. L. A. Turnbull, A. Ozgul, W. Accouche, R. Baxter, L. ChongSeng, J. C. Currie, N. Doak, D. M. Hansen, P. Pistorius, H. Richards, J. van de Crommenacker, R. von Brandis, F. Fleischer-Dogley, N. Bunbury, Persistence of distinctive morphotypes in the native range of the CITES-listed Aldabra giant tortoise. *Ecol. Evol.* **5**, 5499–5508 (2015).

13. D. Bourn, The size, structure and distribution of giant tortoise population of Aldabra. *Philos. Trans. R. Soc. Lond. B Biol. Sci.* **282**, 139–175 (1978).

14. F. G. Çilingir, D. Hansen, N. Bunbury, E. Postma, R. Baxter, L. Turnbull, A. Ozgul, C. Grossen, Low-coverage reduced representation sequencing reveals subtle within-island genetic structure in Aldabra giant tortoises. *Ecol. Evol.* **12**, e8739 (2022).

15. A. S. Cheke, R. Bourn, "Unequal struggle—How humans displaced the domiance of tortoises in island ecosystems" in *Western Indian Ocean Tortoises* (Siri Scientific, 2014), pp. 31–120.

16. G. Stelzer, N. Rosen, I. Plaschkes, S. Zimmerman, M. Twik, S. Fishilevich, T. I. Stein, R. Nudel, I. Lieder, Y. Mazor, S. Kaplan, D. Dahary, D. Warshawsky, Y. Guan-Golan, A. Kohn, N. Rappaport, M. Safran, D. Lancet, The genecards suite: From gene data mining to disease genome sequence analyses. *Curr. Protoc. Bioinformatics* **54**, 1.30.1–1.30.33 (2016).

17. C. Uggenti, A. Lepelley, M. Depp, A. P. Badrock, M. P. Rodero, M.-T. El-Daher, G. I. Rice, S. Dhir, A. P. Wheeler, A. Dhir, W. Albawardi, M.-L. Frémond, L. Seabra, J. Doig, N. Blair, M. J. Martin-Niclos, E. D. Mina, A. Rubio-Roldán, J. L. García-Pérez, D. Sproul, J. Rehwinkel, J. Hertzog, A. Boland-Auge, R. Olaso, J.-F. Deleuze, J. Baruteau, K. Brochard, J. Buckley, V. Cavallera, C. Cereda, L. M. H. De Waele, A. Dobbie, D. Doummar, F. Elmslie, M. Koch-Hogrebe, R. Kumar, K. Lamb, J. H. Livingston, A. Majumdar, C. M. Lorenço, S. Orcesi, S. Peudenier, K. Rostasy, C. A. Salmon, C. Scott, D. Tonduti, G. Touati, M. Valente, H. van der Linden, H. Van Esch, M. Vermelle, K. Webb, A. P. Jackson, M. A. M. Reijns, N. Gilbert, Y. J. Crow, cGAS-mediated induction of type I interferon due to inborn errors of histone pre-mRNA processing. *Nat. Genet.* **52**, 1364–1372 (2020).

18. L. Naesens, J. Nemegeer, F. Roelens, L. Vallaeys, M. Meuwissen, K. Janssens, P. Verloo, B. Ogunjimi, D. Hemelsoet, Program for Undiagnosed Rare Diseases (UD-PrOZA), L. Hoste, L. Roels, M. De Bruyne, E. De Baere, J. Van Dorpe, A. Dendooven, A. Sieben, G. I. Rice, T. Kerre, R. Beyaert, C. Uggenti, Y. J. Crow, S. J. Tavernier, J. Maelfait, F. Haerynck, Mutations in RNU7-1 weaken secondary RNA structure, induce MCP-1 and CXCL10 in CSF, and result in Aicardi-Goutières syndrome with severe end-organ involvement. *J. Clin. Immunol.* **42**, 962–974 (2022).

19. D. Lessel, B. Saha, F. Hisama, B. Kaymakamzade, G. Nurlu, Y. Gursoy-Özdemir, H. Thiele, P. Nürnberg, G. M. Martin, C. Kubisch, J. Oshima, Atypical Aicardi-Goutieres syndrome: Is the WRN locus a modifier? *Am. J. Med. Genet. A* **164**, 2510–2513 (2014).

20. A. Seluanov, C. Hine, J. Azpurua, M. Feigenson, M. Bozzella, Z. Mao, K. C. Catania, V. Gorbunova, Hypersensitivity to contact inhibition provides a clue to cancer resistance of naked mole-rat. *Proc. Natl. Acad. Sci. U.S.A.* **106**, 19352–19357 (2009).

21. X. Tian, J. Azpurua, C. Hine, A. Vaidya, M. Myakishev-Rempel, J. Ablaeva, Z. Mao, E. Nevo, V. Gorbunova, A. Seluanov, High-molecular-mass hyaluronan mediates the cancer resistance of the naked mole rat. *Nature* **499**, 346–349 (2013).

22. Z. Zhang, X. Tian, J. Y. Lu, K. Boit, J. Ablaeva, F. T. Zakusilo, S. Emmrich, D. Firsanov, E. Rydkina, S. A. Biashad, Q. Lu, A. Tyshkovskiy, V. N. Gladyshev, S. Horvath, A. Seluanov, V. Gorbunova, Increased hyaluronan by naked mole-rat HAS2 improves healthspan in mice. *Nature* **621**, 196–205 (2023).

23. N. Braidy, G. J. Guillemin, H. Mansour, T. Chan-Ling, A. Poljak, R. Grant, Age related changes in NAD+ metabolism oxidative stress and Sirt1 activity in wistar rats. *PLOS ONE* **6**, e19194 (2011).

24. D. Szklarczyk, R. Kirsch, M. Koutrouli, K. Nastou, F. Mehryary, R. Hachilif, A. L. Gable, T. Fang, N. T. Doncheva, S. Pyysalo, P. Bork, L. J. Jensen, C. von Mering, The STRING database in 2023: Protein-protein association networks and functional enrichment analyses for any sequenced genome of interest. *Nucleic Acids Res.* **51**, D638–D646 (2023).

25. M. Campolo, S. Oricco, P. Cavicchio, S. Piga, V. Ulivi, M. Poggi, R. Zanatta, M. Iannaccone, Echocardiographic evaluation of four giant Aldabra tortoises (*Aldabrachelys gigantea*). *Vet. Rec. Open* **6**, e000274 (2019).

26. P. Notin, M. Dias, J. Frazer, J. Marchena-Hurtada, A. Gomez, D. Marks, Y. Gal, Tranception: Protein fitness prediction with autoregressive transformers and inference-time retrieval. arXiv:2205.13760 [cs.LG] (2022).

27. J. Meier, R. Rao, R. Verkuil, J. Liu, T. Sercu, A. Rives, Language models enable zero-shot prediction of the effects of mutations on protein function. bioRxiv 450648 (2021). https://doi.org/10.1101/2021.07.09.450648.

28. E. Nijkamp, J. A. Ruffolo, E. N. Weinstein, N. Naik, A. Madani, ProGen2: Exploring the boundaries of protein language models. *Cell Syst.* **14**, 968–978.e3 (2023).

29. D. Hesslow, N. Zanichelli, P. Notin, I. Poli, D. Marks, RITA: A study on scaling up generative protein sequence models. arXiv:2205.05789 [q-bio.QM] (2022).

30. P. Notin, A. W. Kollasch, D. Ritter, L. van Niekerk, S. Paul, H. Spinner, N. Rollins, A. Shaw, R. Weitzman, J. Frazer, M. Dias, D. Franceschi, R. Orenbuch, Y. Gal, D. S. Marks, ProteinGym: Large-scale benchmarks for protein design and fitness prediction. bioRxiv 570727 (2023). https://doi.org/10.1101/2023.12.07.570727.

31. C. Marquet, M. Heinzinger, T. Olenyi, C. Dallago, K. Erckert, M. Bernhofer, D. Nechaev, B. Rost, Embeddings from protein language models predict conservation and variant effects. *Hum. Genet.* **141**, 1629–1647 (2022).

32. R. Tacutu, D. Thornton, E. Johnson, A. Budovsky, D. Barardo, T. Craig, E. Diana, G. Lehmann, D. Toren, J. Wang, V. E. Fraifeld, J. P. de Magalhães, Human Ageing Genomic Resources: New and updated databases. *Nucleic Acids Res.* **46**, D1083–D1090 (2018).

33. C. Rice, P. K. Shastrula, A. V. Kossenkov, R. Hills, D. M. Baird, L. C. Showe, T. Doukov, S. Janicki, E. Skordalakes, Structural and functional analysis of the human POT1-TPP1 telomeric complex. *Nat. Commun.* **8**, 14928 (2017).

34. C. Bettegowda, N. Agrawal, Y. Jiao, M. Sausen, L. D. Wood, R. H. Hruban, F. J. Rodriguez, D. P. Cahill, R. McLendon, G. Riggins, V. E. Velculescu, S. M. Oba-Shinjo, S. K. N. Marie, B. Vogelstein, D. Bigner, H. Yan, N. Papadopoulos, K. W. Kinzler, Mutations in CIC and FUBP1 contribute to human oligodendroglioma. *Science* **333**, 1453–1455 (2011).

35. T. H. Torigoe, D. C. Willcox, M. Shimabukuro, M. Higa, M. Gerschenson, A. Andrukhiv, M. Suzuki, B. J. Morris, R. Chen, G. S. Gojanovich, R. C. Allsopp, B. J. Willcox, Novel protective

effect of the FOXO3 longevity genotype on mechanisms of cellular aging in Okinawans. *NPJ Aging* **10**, 18 (2024).

36. J. Jumper, R. Evans, A. Pritzel, T. Green, M. Figurnov, O. Ronneberger, K. Tunyasuvunakool, R. Bates, A. Žídek, A. Potapenko, A. Bridgland, C. Meyer, S. A. A. Kohl, A. J. Ballard, A. Cowie, B. Romera-Paredes, S. Nikolov, R. Jain, J. Adler, T. Back, S. Petersen, D. Reiman, E. Clancy, M. Zielinski, M. Steinegger, M. Pacholska, T. Berghammer, S. Bodenstein, D. Silver, O. Vinyals, A. W. Senior, K. Kavukcuoglu, P. Kohli, D. Hassabis, Highly accurate protein structure prediction with AlphaFold. *Nature* **596**, 583–589 (2021).

37. UniProt Consortium, UniProt: The universal protein knowledgebase in 2023. *Nucleic Acids Res.* **51**, D523–D531 (2023).

38. M. E. G. de Araujo, A. Naschberger, B. G. Fürnrohr, T. Stasyk, T. Dunzendorfer-Matt, S. Lechner, S. Welti, L. Kremser, G. Shivalingaiah, M. Offterdinger, H. H. Lindner, L. A. Huber, K. Scheffzek, Crystal structure of the human lysosomal mTORC1 scaffold complex and its impact on signaling. *Science* **358**, 377–381 (2017).

39. M. Meerang, D. Ritz, S. Paliwal, Z. Garajova, M. Bosshard, N. Mailand, P. Janscak, U. Hübscher, H. Meyer, K. Ramadan, The ubiquitin-selective segregase VCP/p97 orchestrates the response to DNA double-strand breaks. *Nat. Cell Biol.* **13**, 1376–1382 (2011).

40. Y. Ovadya, T. Landsberger, H. Leins, E. Vadai, H. Gal, A. Biran, R. Yosef, A. Sagiv, A. Agrawal, A. Shapira, J. Windheim, M. Tsoory, R. Schirmbeck, I. Amit, H. Geiger, V. Krizhanovsky, Impaired immune surveillance accelerates accumulation of senescent cells and aging. *Nat. Commun.* **9**, 5435 (2018).

41. A. T. Lu, Z. Fei, A. Haghani, T. R. Robeck, J. A. Zoller, C. Z. Li, R. Lowe, Q. Yan, J. Zhang, H. Vu, J. Ablaeva, V. A. Acosta-Rodriguez, D. M. Adams, J. Almunia, A. Aloysius, R. Ardehali, A. Arneson, C. S. Baker, G. Banks, K. Belov, N. C. Bennett, P. Black, D. T. Blumstein, E. K. Bors, C. E. Breeze, R. T. Brooke, J. L. Brown, G. G. Carter, A. Caulton, J. M. Cavin, L. Chakrabarti, I. Chatzistamou, H. Chen, K. Cheng, P. Chiavellini, O. W. Choi, S. M. Clarke, L. N. Cooper, M. L. Cossette, J. Day, J. DeYoung, S. DiRocco, C. Dold, E. E. Ehmke, C. K.

Emmons, S. Emmrich, E. Erbay, C. Erlacher-Reid, C. G. Faulkes, S. H. Ferguson, C. J. Finno, J. E. Flower, J. M. Gaillard, E. Garde, L. Gerber, V. N. Gladyshev, V. Gorbunova, R. G. Goya, M. J. Grant, C. B. Green, E. N. Hales, M. B. Hanson, D. W. Hart, M. Haulena, K. Herrick, A. N. Hogan, C. J. Hogg, T. A. Hore, T. Huang, J. C. I. Belmonte, A. J. Jasinska, G. Jones, E. Jourdain, O. Kashpur, H. Katcher, E. Katsumata, V. Kaza, H. Kiaris, M. S. Kobor, P. Kordowitzki, W. R. Koski, M. Krützen, S. B. Kwon, B. Larison, S. G. Lee, M. Lehmann, J. F. Lemaitre, A. J. Levine, C. Li, X. Li, A. R. Lim, D. T. S. Lin, D. M. Lindemann, T. J. Little, N. Macoretta, D. Maddox, C. O. Matkin, J. A. Mattison, M. McClure, J. Mergl, J. J. Meudt, G. A. Montano, K. Mozhui, J. Munshi-South, A. Naderi, M. Nagy, P. Narayan, P. W. Nathanielsz, N. B. Nguyen, C. Niehrs, J. K. O'Brien, P. O'Tierney Ginn, D. T. Odom, A. G. Ophir, S. Osborn, E. A. Ostrander, K. M. Parsons, K. C. Paul, M. Pellegrini, K. J. Peters, A. B. Pedersen, J. L. Petersen, D. W. Pietersen, G. M. Pinho, J. Plassais, J. R. Poganik, N. A. Prado, P. Reddy, B. Rey, B. R. Ritz, J. Robbins, M. Rodriguez, J. Russell, E. Rydkina, L. L. Sailer, A. B. Salmon, A. Sanghavi, K. M. Schachtschneider, D. Schmitt, T. Schmitt, L. Schomacher, L. B. Schook, K. E. Sears, A. W. Seifert, A. Seluanov, A. B. A. Shafer, D. Shanmuganayagam, A. V. Shindyapina, M. Simmons, K. Singh, I. Sinha, J. Slone, R. G. Snell, E. Soltanmaohammadi, M. L. Spangler, M. C. Spriggs, L. Staggs, N. Stedman, K. J. Steinman, D. T. Stewart, V. J. Sugrue, B. Szladovits, J. S. Takahashi, M. Takasugi, E. C. Teeling, M. J. Thompson, B. Van Bonn, S. C. Vernes, D. Villar, H. V. Vinters, M. C. Wallingford, N. Wang, R. K. Wayne, G. S. Wilkinson, C. K. Williams, R. W. Williams, X. W. Yang, M. Yao, B. G. Young, B. Zhang, Z. Zhang, P. Zhao, Y. Zhao, W. Zhou, J. Zimmermann, J. Ernst, K. Raj, S. Horvath, Universal DNA methylation age across mammalian tissues. *Nat Aging* **3**, 1144–1166 (2023).

42. K. Seale, S. Horvath, A. Teschendorff, N. Eynon, S. Voisin, Making sense of the ageing methylome. *Nat. Rev. Genet.* **23**, 585–605 (2022).

43. G. Jenkinson, E. Pujadas, J. Goutsias, A. P. Feinberg, Potential energy landscapes identify the information-theoretic nature of the epigenome. *Nat. Genet.* **49**, 719–729 (2017).

44. G. Landan, N. M. Cohen, Z. Mukamel, A. Bar, A. Molchadsky, R. Brosh, S. Horn-Saban, D. A. Zalcenstein, N. Goldfinger, A. Zundelevich, E. N. Gal-Yam, V. Rotter, A. Tanay, Epigenetic

polymorphism and the stochastic formation of differentially methylated regions in normal and cancerous tissues. *Nat. Genet.* **44**, 1207–1214 (2012).

45. G. Hannum, J. Guinney, L. Zhao, L. Zhang, G. Hughes, S. Sadda, B. Klotzle, M. Bibikova, J.-B. Fan, Y. Gao, R. Deconde, M. Chen, I. Rajapakse, S. Friend, T. Ideker, K. Zhang, Genome-wide methylation profiles reveal quantitative views of human aging rates. *Mol. Cell* **49**, 359–367 (2013).

46. A. M. Tsankov, M. H. Wadsworth, V. Akopian, J. Charlton, S. J. Allon, A. Arczewska, B. E. Mead, R. S. Drake, Z. D. Smith, T. S. Mikkelsen, A. K. Shalek, A. Meissner, Loss of DNA methyltransferase activity in primed human ES cells triggers increased cell-cell variability and transcriptional repression. *Development* **146**, dev174722 (2019).

47. Y. Fang, Z. Ji, W. Zhou, J. Abante, M. A. Koldobskiy, H. Ji, A. P. Feinberg, DNA methylation entropy is associated with DNA sequence features and developmental epigenetic divergence. *Nucleic Acids Res.* **51**, 2046–2065 (2023).

48. H.-T. Wang, F.-H. Xiao, Z.-L. Gao, L.-Y. Guo, L.-Q. Yang, G.-H. Li, Q.-P. Kong, Methylation entropy landscape of Chinese long-lived individuals reveals lower epigenetic noise related to human healthy aging. *Aging Cell* **23**, e14163 (2024).

49. E. Rafikova, N. Nemirovich-Danchenko, A. Ogmen, A. Parfenenkova, A. Velikanova, S. Tikhonov, L. Peshkin, K. Rafikov, O. Spiridonova, Y. Belova, T. Glinin, A. Egorova, M. Batin, Open Genes—A new comprehensive database of human genes associated with aging and longevity. *Nucleic Acids Res.* **52**, D950–D962 (2024).

50. H. Heyn, N. Li, H. J. Ferreira, S. Moran, D. G. Pisano, A. Gomez, J. Diez, J. V. Sanchez-Mut, F. Setien, F. J. Carmona, A. A. Puca, S. Sayols, M. A. Pujana, J. Serra-Musach, I. Iglesias-Platas, F. Formiga, A. F. Fernandez, M. F. Fraga, S. C. Heath, A. Valencia, I. G. Gut, J. Wang, M. Esteller, Distinct DNA methylomes of newborns and centenarians. *Proc. Natl. Acad. Sci. U.S.A.* **109**, 10522–10527 (2012).

51. A. E. Teschendorff, U. Menon, A. Gentry-Maharaj, S. J. Ramus, D. J. Weisenberger, H. Shen, M. Campan, H. Noushmehr, C. G. Bell, A. P. Maxwell, D. A. Savage, E. Mueller-Holzner, C. Marth, G. Kocjan, S. A. Gayther, A. Jones, S. Beck, W. Wagner, P. W. Laird, I. J. Jacobs, M. Widschwendter, Age-dependent DNA methylation of genes that are suppressed in stem cells is a hallmark of cancer. *Genome Res.* **20**, 440–446 (2010).

52. K. Day, L. L. Waite, A. Thalacker-Mercer, A. West, M. M. Bamman, J. D. Brooks, R. M. Myers, D. Absher, Differential DNA methylation with age displays both common and dynamic features across human tissues that are influenced by CpG landscape. *Genome Biol.* **14**, R102 (2013).

53. V. K. Rakyan, T. A. Down, S. Maslau, T. Andrew, T.-P. Yang, H. Beyan, P. Whittaker, O. T. McCann, S. Finer, A. M. Valdes, R. D. Leslie, P. Deloukas, T. D. Spector, Human aging-associated DNA hypermethylation occurs preferentially at bivalent chromatin domains. *Genome Res.* **20**, 434–439 (2010).

54. J. T. Bell, A. A. Pai, J. K. Pickrell, D. J. Gaffney, R. Pique-Regi, J. F. Degner, Y. Gilad, J. K. Pritchard, DNA methylation patterns associate with genetic and gene expression variation in HapMap cell lines. *Genome Biol.* **12**, R10 (2011).

55. C. López-Otín, M. A. Blasco, L. Partridge, M. Serrano, G. Kroemer, The hallmarks of aging. *Cell* **153**, 1194–1217 (2013).

56. C. López-Otín, M. A. Blasco, L. Partridge, M. Serrano, G. Kroemer, Hallmarks of aging: An expanding universe. *Cell* **186**, 243–278 (2023).

57. G. Barja, S. Cadenas, C. Rojas, R. Pérez-Campo, M. López-Torres, Low mitochondrial free radical production per unit O_2 consumption can explain the simultaneous presence of high longevity and high aerobic metabolic rate in birds. *Free Radic. Res.* **21**, 317–327 (1994).

58. J. Gómez, N. Mota-Martorell, M. Jové, R. Pamplona, G. Barja, Mitochondrial ROS production, oxidative stress and aging within and between species: Evidences and recent advances on this aging effector. *Exp. Gerontol.* **174**, 112134 (2023).

59. A. K. Brunet-Rossinni, S. N. Austad, Ageing studies on bats: A review. *Biogerontology* **5**, 211–222 (2004).

60. E. Santos-Pujol, A. Noguera-Castells, M. Casado-Pelaez, C. A. García-Prieto, C. Vasallo, I. Campillo-Marcos, C. Quero-Dotor, E. Crespo-García, A. Bueno-Costa, F. Setién, G. Ferrer, V. Davalos, E. Mereu, R. Pluvinet, C. Arribas, C. de la Torre, F. Villavicencio, L. Sumoy, I. Granada, N. S. Coles, P. Acha, F. Solé, M. Mallo, C. Mata, S. Peregrina, T. Gabaldón, M. Llirós, M. Pujolassos, R. Carreras-Torres, A. Lluansí, L. J. García-Gil, X. Aldeguer, S. Samino, P. Torné, J. Ribalta, M. Guardiola, N. Amigó, O. Yanes, P. Martínez, R. Sánchez-Vázquez, M. A. Blasco, J. Oviedo, B. Lemos, J. Rius-Bonet, M. Torrubiano, M. Massip-Salcedo, K. A. Khidir, T. H. Cao, P. A. Quinn, D. J. L. Jones, S. Macip, E. Brigos-Barril, M. Moldes, F. Barteri, G. Muntané, H. Laayouni, A. Navarro, M. Esteller, The multiomics blueprint of the individual with the most extreme lifespan. *Cell Rep. Med.* **6**, 102368 (2025).

61. Z. Koch, A. Li, D. S. Evans, S. Cummings, T. Ideker, Somatic mutation as an explanation for epigenetic aging. *Nat. Aging* **5**, 709–719 (2025).

62. H. Cheng, G. T. Concepcion, X. Feng, H. Zhang, H. Li, Haplotype-resolved de novo assembly using phased assembly graphs with hifiasm. *Nat. Methods* **18**, 170–175 (2021).

63. J. Ghurye, M. Pop, S. Koren, D. Bickhart, C.-S. Chin, Scaffolding of long read assemblies using long range contact information. *BMC Genomics* **18**, 527 (2017).

64. M. Manni, M. R. Berkeley, M. Seppey, E. M. Zdobnov, BUSCO: Assessing genomic data quality and beyond. *Curr. Protoc.* **1**, e323 (2021).

65. E. M. Zdobnov, D. Kuznetsov, F. Tegenfeldt, M. Manni, M. Berkeley, E. V. Kriventseva, OrthoDB in 2020: Evolutionary and functional annotations of orthologs. *Nucleic Acids Res.* **49**, D389–D393 (2021).

66. H. Li, R. Durbin, Fast and accurate short read alignment with Burrows-Wheeler transform. *Bioinformatics* **25**, 1754–1760 (2009).

67. G. A. Van der Auwera, M. O. Carneiro, C. Hartl, R. Poplin, G. Del Angel, A. Levy-Moonshine, T. Jordan, K. Shakir, D. Roazen, J. Thibault, E. Banks, K. V. Garimella, D. Altshuler, S. Gabriel, M. A. DePristo, From FastQ data to high confidence variant calls: The Genome Analysis Toolkit best practices pipeline. *Curr. Protoc. Bioinformatics* **43**, 11.10.1–11.10.33 (2013).

68. T. S. Korneliussen, A. Albrechtsen, R. Nielsen, ANGSD: Analysis of next generation sequencing data. *BMC Bioinformatics* **15**, 356 (2014).

69. J. Meisner, A. Albrechtsen, Inferring population structure and admixture proportions in low-depth NGS data. *Genetics* **210**, 719–731 (2018).

70. J. M. Flynn, R. Hubley, C. Goubert, J. Rosen, A. G. Clark, C. Feschotte, A. F. Smit, RepeatModeler2 for automated genomic discovery of transposable element families. *Proc. Natl. Acad. Sci. U.S.A.* **117**, 9451–9457 (2020).

71. M. Tarailo-Graovac, N. Chen, Using RepeatMasker to identify repetitive elements in genomic sequences. *Curr. Protoc. Bioinformatics* **4**, Unit 4.10 (2009).

72. A. Rhie, S. A. McCarthy, O. Fedrigo, J. Damas, G. Formenti, S. Koren, M. Uliano-Silva, W. Chow, A. Fungtammasan, J. Kim, C. Lee, B. J. Ko, M. Chaisson, G. L. Gedman, L. J. Cantin, F. Thibaud-Nissen, L. Haggerty, I. Bista, M. Smith, B. Haase, J. Mountcastle, S. Winkler, S. Paez, J. Howard, S. C. Vernes, T. M. Lama, F. Grutzner, W. C. Warren, C. N. Balakrishnan, D. Burt, J. M. George, M. T. Biegler, D. Iorns, A. Digby, D. Eason, B. Robertson, T. Edwards, M. Wilkinson, G. Turner, A. Meyer, A. F. Kautt, P. Franchini, H. W. Detrich, H. Svardal, M. Wagner, G. J. P. Naylor, M. Pippel, M. Malinsky, M. Mooney, M. Simbirsky, B. T. Hannigan, T. Pesout, M. Houck, A. Misuraca, S. B. Kingan, R. Hall, Z. Kronenberg, I. Sović, C. Dunn, Z. Ning, A. Hastie, J. Lee, S. Selvaraj, R. E. Green, N. H. Putnam, I. Gut, J. Ghurye, E. Garrison, Y. Sims, J. Collins, S. Pelan, J. Torrance, A. Tracey, J. Wood, R. E. Dagnew, D. Guan, S. E. London, D. F. Clayton, C. V. Mello, S. R. Friedrich, P. V. Lovell, E. Osipova, F. O. Al-Ajli, S. Secomandi, H. Kim, C. Theofanopoulou, M. Hiller, Y. Zhou, R. S. Harris, K. D. Makova, P. Medvedev, J. Hoffman, P. Masterson, K. Clark, F. Martin, K. Howe, P. Flicek, B. P. Walenz, W. Kwak, H. Clawson, M. Diekhans, L. Nassar, B. Paten, R. H. S. Kraus, A. J. Crawford, M. T. P. Gilbert, G. Zhang, B. Venkatesh, R. W. Murphy, K.-P. Koepfli, B. Shapiro, W. E. Johnson, F. Di

Palma, T. Marques-Bonet, E. C. Teeling, T. Warnow, J. M. Graves, O. A. Ryder, D. Haussler, S. J. O'Brien, J. Korlach, H. A. Lewin, K. Howe, E. W. Myers, R. Durbin, A. M. Phillippy, E. D. Jarvis, Towards complete and error-free genome assemblies of all vertebrate species. *Nature* **592**, 737–746 (2021).

73. A. Dobin, C. A. Davis, F. Schlesinger, J. Drenkow, C. Zaleski, S. Jha, P. Batut, M. Chaisson, T. R. Gingeras, STAR: Ultrafast universal RNA-seq aligner. *Bioinformatics* **29**, 15–21 (2013).

74. T. Brůna, K. J. Hoff, A. Lomsadze, M. Stanke, M. Borodovsky, BRAKER2: Automatic eukaryotic genome annotation with GeneMark-EP+ and AUGUSTUS supported by a protein database. *NAR Genom. Bioinform.* **3**, lqaa108 (2021).

75. G. Gremme, V. Brendel, M. Sparks, S. Kurtz, Engineering a software tool for gene structure prediction in higher organisms. *Inf. Softw. Technol.* **47**, 965–978 (2008).

76. M. Stanke, R. Steinkamp, S. Waack, B. Morgenstern, AUGUSTUS: A web server for gene finding in eukaryotes. *Nucleic Acids Res.* **32**, W309–W312 (2004).

77. V. Kapatral, I. Anderson, N. Ivanova, G. Reznik, T. Los, A. Lykidis, A. Bhattacharyya, A. Bartman, W. Gardner, G. Grechkin, L. Zhu, O. Vasieva, L. Chu, Y. Kogan, O. Chaga, E. Goltsman, A. Bernal, N. Larsen, M. D'Souza, T. Walunas, G. Pusch, R. Haselkorn, M. Fonstein, N. Kyrpides, R. Overbeek, Genome sequence and analysis of the oral bacterium *Fusobacterium nucleatum* strain ATCC 25586. *J. Bacteriol.* **184**, 2005–2018 (2002).

78. R. Overbeek, N. Larsen, T. Walunas, M. D'Souza, G. Pusch, E. Selkov, K. Liolios, V. Joukov, D. Kaznadzey, I. Anderson, A. Bhattacharyya, H. Burd, W. Gardner, P. Hanke, V. Kapatral, N. Mikhailova, O. Vasieva, A. Osterman, V. Vonstein, M. Fonstein, N. Ivanova, N. Kyrpides, The ERGO genome analysis and discovery system. *Nucleic Acids Res.* **31**, 164–171 (2003).

79. B. Buchfink, K. Reuter, H.-G. Drost, Sensitive protein alignments at tree-of-life scale using DIAMOND. *Nat. Methods* **18**, 366–368 (2021).

80. A. Löytynoja, Phylogeny-aware alignment with PRANK. *Methods Mol. Biol.* **1079**, 155–170 (2014).

81. Z. Yang, PAML 4: Phylogenetic analysis by maximum likelihood. *Mol. Biol. Evol.* **24**, 1586–1591 (2007).

82. R Core Team, R: A language and environment for statistical computing, R Foundation for Statistical Computing (2020); www.R-project.org/.

83. A. Alexa, J. Rahnenfuhrer, topGO: Enrichment Analysis for Gene Ontology, version R package version 2.50.0 (2022).

84. E. Dolzhenko, A. D. Smith, Using beta-binomial regression for high-precision differential methylation analysis in multifactor whole-genome bisulfite sequencing experiments. *BMC Bioinformatics* **15**, 215 (2014).

85. D. Lee, B. Koo, J. Yang, S. Kim, Metheor: Ultrafast DNA methylation heterogeneity calculation from bisulfite read alignments. *PLOS Comput. Biol.* **19**, e1010946 (2023).

86. T. C. Terwilliger, D. Liebschner, T. I. Croll, C. J. Williams, A. J. McCoy, B. K. Poon, P. V. Afonine, R. D. Oeffner, J. S. Richardson, R. J. Read, P. D. Adams, AlphaFold predictions are valuable hypotheses and accelerate but do not replace experimental structure determination. *Nat. Methods* **21**, 110–116 (2024).

87. M. Steinegger, J. Söding, MMseqs2 enables sensitive protein sequence searching for the analysis of massive data sets. *Nat. Biotechnol.* **35**, 1026–1028 (2017).

88. M. Mirdita, M. Steinegger, J. Söding, MMseqs2 desktop and local web server app for fast, interactive sequence searches. *Bioinforma. Oxf. Engl.* **35**, 2856–2858 (2019).

89. M. Mirdita, K. Schütze, Y. Moriwaki, L. Heo, S. Ovchinnikov, M. Steinegger, ColabFold: Making protein folding accessible to all. *Nat. Methods* **19**, 679–682 (2022).

90. N. T. Doncheva, J. H. Morris, H. Holze, R. Kirsch, K. C. Nastou, Y. Cuesta-Astroz, T. Rattei, D. Szklarczyk, C. von Mering, L. J. Jensen, Cytoscape stringApp 2.0: Analysis and visualization of heterogeneous biological networks. *J. Proteome Res.* **22**, 637–646 (2023).

91. M. Utriainen, J. H. Morris, clusterMaker2: A major update to clusterMaker, a multi-algorithm clustering app for Cytoscape. *BMC Bioinformatics* **24**, 134 (2023).

92. M. Legeay, N. T. Doncheva, J. H. Morris, L. J. Jensen, Visualize omics data on networks with Omics Visualizer, a Cytoscape App. *F1000Res.* **9**, 157 (2020).

93. R. A. Nicholls, M. Fischer, S. McNicholas, G. N. Murshudov, Conformation-independent structural comparison of macromolecules with ProSMART. *Acta Crystallogr. D Biol. Crystallogr.* **70**, 2487–2499 (2014).

94. Museum of Comparative Zoology, History, Harvard University (2026); www.mcz.harvard.edu/herpetology-history.

95. A. Loveridge: Our tortoises. Part 1. The truth about Jonathan. *Wirebird III*. **88**, 704–706 (1962).

96. S. Kettle, "Meet Jonathan, St Helena's 182-year-old giant tortoise," *BBC News*, 13 March 2014; www.bbc.com/news/magazine-26543021.

97. S. Greenhill, "Still sprightly at 183, the oldest creature on Earth," *Daily Mail*, 8 January 2016; www.pressreader.com/uk/daily-mail/20160108/281526520041137.

98. R. Richardson, Early Postcards of St. Helena, 1900-1910, *The Postcard Album* (2026); www.tpa-project.info/.

99. Christie's, The Quentin Keynes Collection-Live Auction 6890 (2004); www.christies.com/en/lot/lot-4256031.

100. J. E. Moustakas-Verho, J. Cebra-Thomas, S. F. Gilbert, Patterning of the turtle shell. *Curr. Opin. Genet. Dev*. **45**, 124–131 (2017).

101. R. Floud, B. Harris, "Health, height, and welfare: Britain, 1700–1980" in *Health and Welfare during Industrialization* (University of Chicago Press, 1997), pp. 91–126; www.nber.org/system/files/chapters/c7429/c7429.pdf.

102. Eleif, Photomeasure (2026); www.eleif.net/photomeasure.

103. G. S. Wilkinson, D. M. Adams, A. Haghani, A. T. Lu, J. Zoller, C. E. Breeze, B. D. Arnold, H. C. Ball, G. G. Carter, L. N. Cooper, D. K. N. Dechmann, P. Devanna, N. J. Fasel, A. V. Galazyuk, L. Günther, E. Hurme, G. Jones, M. Knörnschild, E. Z. Lattenkamp, C. Z. Li, F. Mayer, J. A. Reinhardt, R. A. Medellin, M. Nagy, B. Pope, M. L. Power, R. D. Ransome, E. C. Teeling, S. C. Vernes, D. Zamora-Mejías, J. Zhang, P. A. Faure, L. J. Greville, L. G. Herrera M, J. J. Flores-Martínez, S. Horvath, DNA methylation predicts age and provides insight into exceptional longevity of bats. *Nat. Commun.* **12**, 1615 (2021).
